# Fcirc: A comprehensive pipeline for the exploration of fusion linear and circular RNAs

**DOI:** 10.1093/gigascience/giaa054

**Published:** 2020-05-29

**Authors:** Zhaoqing Cai, Hongzhang Xue, Yue Xu, Jens Köhler, Xiaojie Cheng, Yao Dai, Jie Zheng, Haiyun Wang

**Affiliations:** 1 School of Life Sciences and Technology, Tongji University, 1239 Siping Road, Shanghai 200092, China; 2 School of Life Sciences and Biotechnology, Shanghai Jiao Tong University, 800 Dongchuan Road, Shanghai 200240, China; 3 Department of Medical Oncology, Dana-Farber Cancer Institute, 450 Brookline Avenue, Boston, MA 02215, USA

**Keywords:** Fcirc, fusion linear RNA, fusion circRNA, performance benchmarks

## Abstract

**Background:**

In cancer cells, fusion genes can produce linear and chimeric fusion-circular RNAs (f-circRNAs), which are functional in gene expression regulation and implicated in malignant transformation, cancer progression, and therapeutic resistance. For specific cancers, proteins encoded by fusion transcripts have been identified as innovative therapeutic targets (e.g., *EML4-ALK*). Even though RNA sequencing (RNA-Seq) technologies combined with existing bioinformatics approaches have enabled researchers to systematically identify fusion transcripts, specifically detecting f-circRNAs in cells remains challenging owing to their general sparsity and low abundance in cancer cells but also owing to imperfect computational methods.

**Results:**

We developed the Python-based workflow “Fcirc” to identify fusion linear and f-circRNAs from RNA-Seq data with high specificity. We applied Fcirc to 3 different types of RNA-Seq data scenarios: (i) actual synthetic spike-in RNA-Seq data, (ii) simulated RNA-Seq data, and (iii) actual cancer cell–derived RNA-Seq data. Fcirc showed significant advantages over existing methods regarding both detection accuracy (i.e., precision, recall, F-measure) and computing performance (i.e., lower runtimes).

**Conclusion:**

Fcirc is a powerful and comprehensive Python-based pipeline to identify linear and circular RNA transcripts from known fusion events in RNA-Seq datasets with higher accuracy and shorter computing times compared with previously published algorithms. Fcirc empowers the research community to study the biology of fusion RNAs in cancer more effectively.

## Background

Various events such as gene mutations, gene rearrangements, and chromosomal fragile sites are able to induce the formation of fusion genes in the genome of cancer cells [1–6]. These fusion genes can generate linear or fusion circular RNAs (f-circRNAs)—the latter via back-splicing of exons. F-circRNAs are functional in gene expression regulation and are implicated in malignant transformation, cancer cell survival, and therapeutic resistance [7]. Apart from their relevance for cancer cell biology, f-circRNAs also serve as promising biomarker candidates in liquid biopsies owing to their increased stability relative to linear transcripts [8]. Furthermore, proteins encoded by fusion genes represent innovative therapeutic targets in some cancers, thus indicating that the still relatively young field of fusion RNA biology harbours a great potential for drug development. Crizotinib, for example, a tyrosine kinase inhibitor, was approved by the US Food and Drug Administration (FDA) in 2013 for the treatment of patients with non–small cell lung cancer (NSCLC) harbouring *EML4-ALK* rearrangements [9]. Therefore, accurate profiling of fusion linear and circular RNAs is of high scientific interest and provides the basis for functional studies in cancer. Although recent advances in high-throughput RNA-Seq data acquisition have enabled researchers to detect fusion transcripts [10–15] and circRNAs [16–18], the currently available tools for fusion detection still yield a high false discovery rate [19], and current bioinformatics methods cannot be used to identify the whole spectrum of f-circRNAs arising from a specific fusion gene [20].

Therefore, in the present study, we developed “Fcirc,” a comprehensive, accurate, and free-of-charge pipeline to analyse RNA-Seq data for linear and circular RNAs transcribed from fusion genes.

## Materials and Methods

### Datasets used in this study

#### Synthetic spike-in actual RNA-Seq data

To evaluate the performance of different tools for fusion RNA analysis, we took advantage of RNA-Seq data from a study performed by Tembe et al. [21]. In this study, equimolar amounts of 9 synthetic poly-adenylated gene fusion RNA transcripts were pooled and titrated into total RNA of COLO-829 melanoma cells at 10 different concentrations with 2 replicates for each sample: *EWSR1*-*ATF1, TMPRSS2*-*ETV1, EWSR1*-*FLI1, NTRK3*-*ETV6, CD74*-*ROS1, HOOK3*-*RET, EML4*-*ALK, AKAP9*-*BRAF*, and *BRD4*-*NUTM1*. The sequencing data (Illumina HiSeq 2500 system) were made available in FASTQ format in the SRA under accession number SRP043081 and allow researchers to validate novel algorithms for gene fusion detection in a comparative manner.

#### Simulated RNA-Seq data

The simulator art_illumina function in ART [22] was applied to simulate RNA-Seq data. We used the RNA-Seq reads from normal pulmonary microvascular endothelial cells in the NCBI SRA database SRR349695 [23] as background and plugged simulated fusion reads into the background. Two types of fusion reads were designed: (i) those derived from linear transcripts and (ii) those derived from pooled linear and circular transcripts. A total of 47 fusions (Suppl. Table 1) with high, median, and low frequency in cancers were randomly selected from the Catalogue of Somatic Mutations in Cancer (COSMIC) database [24]. The linear fusion reads were artificially generated on the basis of the breakpoint information by joining the upstream and downstream transcript fragments. Eight fusion circRNAs (Suppl. Fig. 1, Suppl. Table 2) were generated in accordance with previous reports [7, 8]. To simulate more linear than circular fusion transcripts at a gene locus, we plugged 2.5 times as many linear fusion reads into the background as circular fusion reads. Different sequencing coverages (20, 50, and 100×) were simulated each with 2 read lengths of 50 and 100 bp.

#### Actual cancer cell–derived RNA-Seq data

Actual cancer cell–derived RNA-Seq data for the identification of f-circRNA were obtained from the BioProject database (accession IDs PRJNA350335 and PRJNA315254). Whereas PRJNA350335 includes sequencing information on 9 lung cancer samples of H3122 cell line harbouring the *EML4*-*ALK* fusion gene [25], PRJNA315254 includes a total of 9 acute leukemia samples, among them NB4 (n = 3), THP1 (n = 1), and primary patient-derived (n = 5) cell lines harbouring the *PML*-*RARα* fusion gene [7].

### The Fcirc pipeline workflow

**Figure 1:**
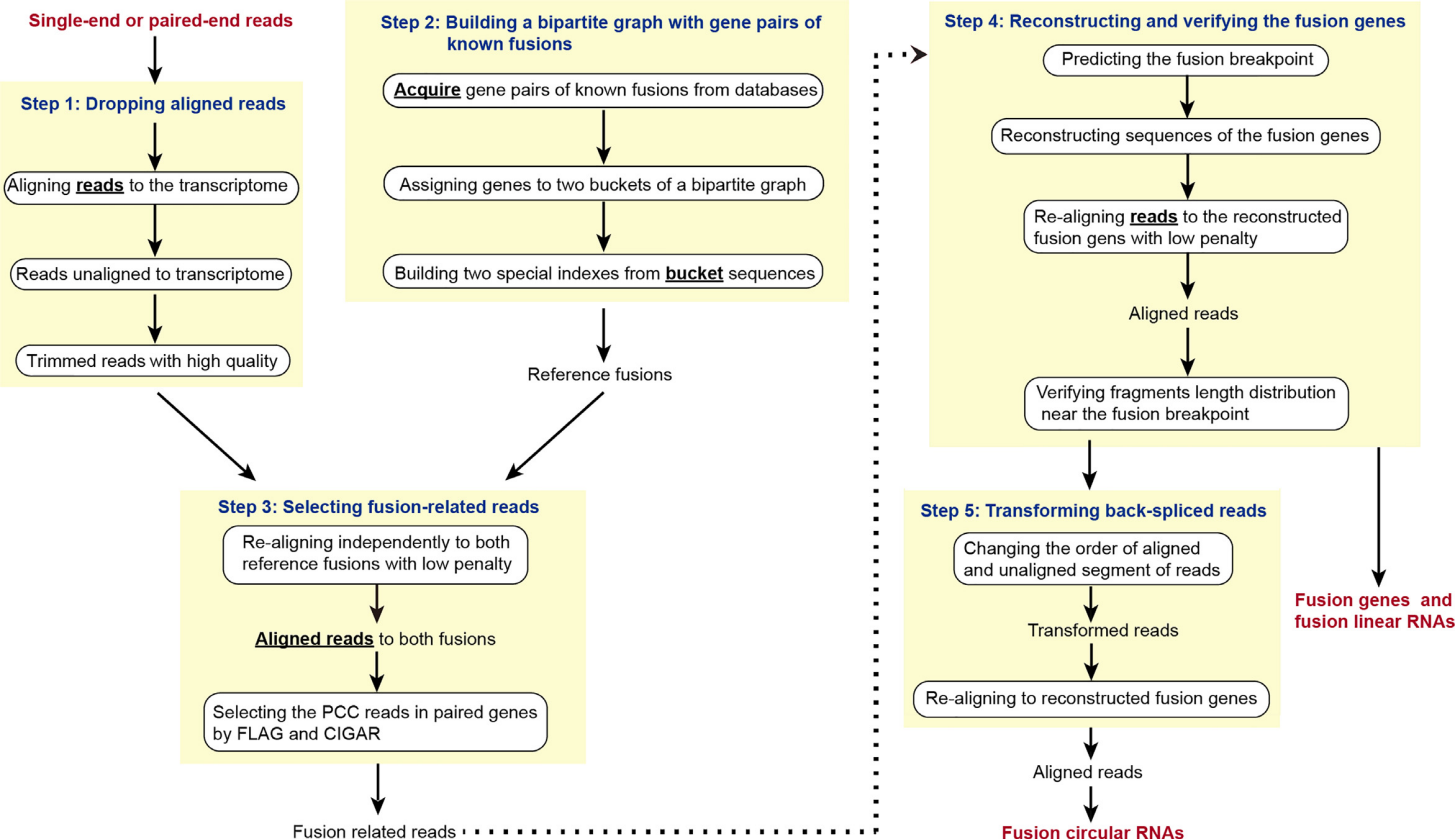
Fcirc pipeline workflow for exploring linear and circular RNAs of known fusions. Schematic depiction of the 5 main steps of the Fcirc workflow, which includes the dropping of aligned reads (step 1), the building of a bipartite graph of known fusion gene pairs (step 2), the selection of fusion-related reads (step 3), as well as the reconstruction and verification of fusion genes for linear (step 4) and fusion circular RNAs (step 5 includes transformation of back-spliced reads).

The “Fcirc” analysis pipeline includes 5 major steps (Fig. 1), and the baseline data input are single-end or paired-end RNA-Seq datasets in FASTQ format. Both raw and cleaned data are acceptable, e.g., after adapter cutting or poor-quality trimming.

#### Step 1: Dropping aligned reads

Reads were aligned to a reference transcriptome with HISAT2 [26] using default parameters. After the first alignment, the aligned reads were dropped by samtools [27] and unaligned reads were kept for further analysis. The unaligned reads were then selected according to FLAG values in SAM file and converted into a file in FASTQ format.

#### Step 2: Building of a bipartite graph of gene pairs of known fusions

Gene pairs of known fusion genes were manually curated from multiple databases, including COSMIC [24], ChimerDB [28], TicDB [29], FARE-CAFE [30], and FusionCancer [31], and the gene sequences of known gene fusions were downloaded from the Ensembl Genome Browser [32]. With this information, we built a “bipartite graph” (also called a “bigraph”) of known fusion gene-pairs. In a bipartite graph vertices (representing individual genes in our study) can be divided into 2 disjoint and independent sets U and V in a way that every edge (representing fusion events between individual genes in our study) connects a vertex in U to one in V. In our case it was possible to generate a bipartite graph because the genes involved in the fusion events did not form a ring of odd vertices. To reduce the computational complexity and time required to search for multiple gene-spanning reads, genes involved in the fusion event were divided into 2 independent sets according to the bipartite graph theory. For example, in the case of the fusion genes *EML4*-*ALK* and *NPM1*-*ALK, EML4* and *NPM1* were included in the same set (U) while *ALK* was included in the independent set (V).

#### Step 3: Selecting fusion-related reads

In the next step, the unaligned reads were independently re-aligned to 2 sets of fusion gene sequences with low penalty. We decreased the maximum and minimum penalty for soft-clipping (–sp 1, 1) and minimum alignment score (–score-min L, 0, -0.8). Other scoring parameters were set as default. After this re-alignment, reads with partial sequence alignment to fusion genes were selected. For single-end RNA-Seq data, reads without a FLAG value of 4 (-F 4) and for paired-end RNA-Seq data, reads with a FLAG value of 4, not 8, or 8, not 4 or 12 in the SAM file were selected (-f 4 -F 8 or -F 4 -f 8 or -f 12), respectively, ensuring that ≥1 read segment was aligned. Reads with paired chiastic clipping (PCC) signal were defined as fusion-related reads. For instance, if a segment of a read was simultaneously aligned to *EML4* with the same FLAG and CIGAR 40S60M values and to *ALK* with FLAG 4 and CIGAR 40M60S, this suggested that 1 segment of *EML4* and the rest from *ALK* were on the same strand.

#### Step 4: Reconstructing and verifying the fusion genes

In the next step, the fusion breakpoint was determined. Therefore, we assumed that fusion-related reads were more likely to cover the respective fusion breakpoint and inferred the exact location from the majority of junction-supported reads. Subsequently, the fusion gene sequence was reconstructed around this predicted fusion breakpoint and the alignment of reads was recalibrated by re-aligning reads to the reconstructed fusion gene with low penalty. To evaluate our assumption, that fusion-related reads uniformly covered the fusion breakpoint, they were split into 2 groups (left and right fragments) in relation to the respective breakpoint. Then, the Wilcoxon signed-rank test was used to evaluate the read distribution by comparing the length of the left and right fragments.

#### Step 5: Transforming back-spliced reads

Circular RNAs transcribed from fusion genes were detected by searching for back-spliced reads. To improve the alignment of back-spliced reads with the reconstructed fusion gene, we changed the order of aligned and unaligned segments of some back-spliced reads to transform back-spliced reads to forward-spliced reads. The transformed reads were then re-aligned to the reconstructed fusion gene to evaluate whether they were truly back-spliced. Those reads covering a back-spliced junction indicated that they were attributable to f-circRNA.

### Fcirc data output format

The resulting output format of Fcirc consists of tables of fusion linear transcripts and of f-circRNAs as well as SAM files for easier visualization of read distribution on the respective fusions.

### Performance benchmarking and evaluation criteria

Fcirc and 6 previously published fusion detection methods (Suppl. Table 3), including Arriba v1.1.0 [33], ChimeraScan v0.4.5 [14], FusionCatcher v1.00 [12], JAFFA v1.09 [15], STAR-Fusion v1.8.1 [13], and STAR-SEQR v0.6.7 [34], were applied to the synthetic spike-in actual RNA-Seq data, simulated data, and actual cancer cell–derived data. To accurately evaluate and compare these tools, we required (i) the number of fusion-supporting reads to be ≥3 and (ii) read-through transcripts to be removed, i.e., 2 genes located on the same chromosome <100,000 bp apart. The computational efficiency of each tool was evaluated by several benchmarking criteria including precision, recall, and *F*-measure, which were defined as follows: precision=TP/(TP+FP); recall=TP/(TP+FN); F=2*(precision*recall)/(precision+recall). TP, FP, and FN represent the true-positive, false-positive, and false-negative results, respectively. The *F*-measure simultaneously considers the effect of precision and recall. We also evaluated the number of supporting reads that were identified by the different methods and which reflect the ability to robustly detect the gene fusion. The final benchmark was the required computing time assuming a computational environment based on Ubuntu Linux with Intel Xeon E5-2620 v4 CPU at 2.10 GHz. Four CPU cores were used for each tool, and the running parameters for each tool are shown in Suppl. Table 4.

## Results

### Evaluation of gene fusions in actual and simulated RNA-Seq datasets from synthetic spike-in experiments

**Figure 2:**
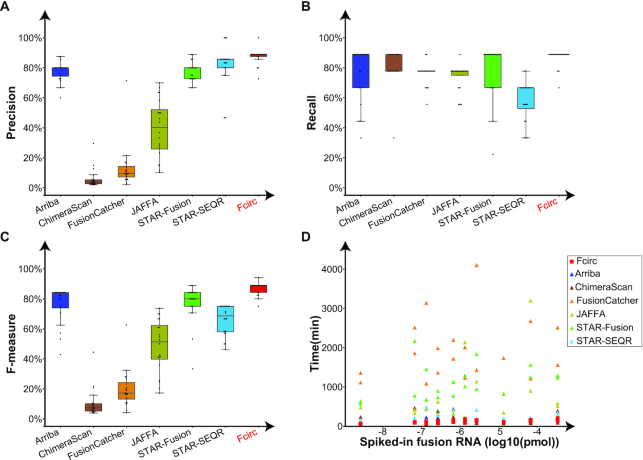
Performance comparison of different gene fusion detection tools in synthetic spike-in actual RNA-Seq data. Comparison of precision (**A**), recall (**B**), *F*-measure (**C**), and computing time (**D**) across 7 fusion detection tools, including Arriba, ChimeraScan, FusionCatcher, JAFFA, STAR-Fusion, STAR-SEQR, and Fcirc. In Fig. 2A-C, the black lines in the box (from top to bottom) represent upper quartile, median and lower quartile, respectively; the top and bottom black line represent upper extreme and lower extreme, respectively; the black dots represent data points.

To compare the performance parameters (i.e., precision, recall, and *F*-measure) of Fcirc with those of other methods, we took advantage of RNA-Seq data from spike-in experiments, which included 9 synthetic cancer-associated fusion genes (*EWSR1*-*ATF1, TMPRSS2*-*ETV1, EWSR1*-*FLI1, NTRK3*-*ETV6, CD74*-*ROS1, HOOK3*-*RET, EML4*-*ALK, AKAP9*-*BRAF*, and *BRD4*-*NUTM1*) [21]. Fcirc achieved not only the highest but also more consistent (small standard deviation [0.05]) precision (87.50%) compared to STAR-SEQR (81.90%) and Arriba (78.00%) (Fig. 2A, Suppl. Table 5-1). ChimeraScan (6.10%) and FusionCatcher (13.4%) exhibited low precision values, thus indicating a high rate of FP results for predictions with these methods. In addition, Fcirc achieved higher recall values (86.68%) than all other methods (ChimeraScan = 80.57%, STAR-Fusion = 78.90%, Arriba = 76.68%, FusionCatcher = 76.14%, JAFFA = 73.92%, and STAR-SEQR = 58.35%) (Fig. 2B, Suppl. Table 5-2) and greater *F*-measures (0.86), indicating a better performance for balancing precision and recall (Fig. 2C, Suppl. Table 6). Finally, Fcirc required less computing time than most of the other methods (Fig. 2D, Suppl. Table 7).

**Figure 3:**
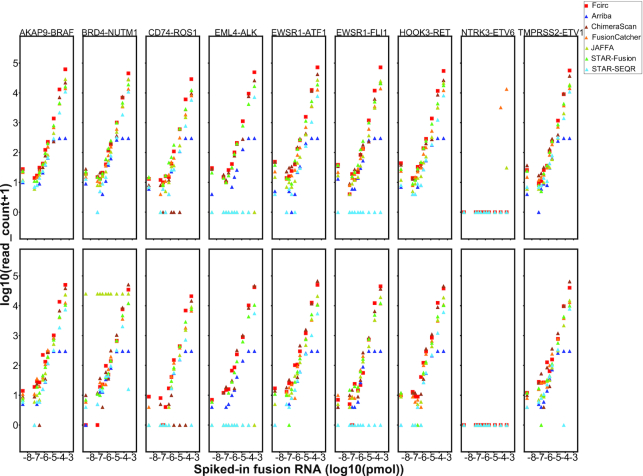
Identification of fusion-supporting reads with different gene fusion detection tools in synthetic spike-in actual RNA-Seq data. The abundance of fusion-supporting reads of 9 spiked-in synthetic fusion RNAs was determined by Arriba, ChimeraScan, FusionCatcher, JAFFA, STAR-Fusion, STAR-SEQR, and Fcirc in total RNA of the melanoma cell line COLO-829 (n = 2 replicates for each fusion gene).

In the next step, we calculated the number of fusion-supporting junctional reads for the different methods with respect to 10 different spike-in concentrations of the pooled synthetic gene fusion RNAs (n = 9, 2 replicates each). Fcirc (red squares) not only identified the highest number of supporting reads but also had a very high accuracy for different spike-in concentrations, indicated by the increasing number of identified supporting reads of a given gene fusion (Fig. 3). The NTRK3-ETV6 fusion RNA construct was basically undetectable by all applied methods.

**Figure 4:**
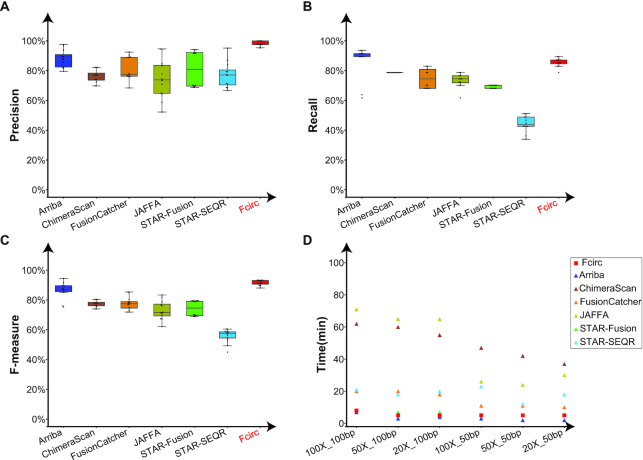
Performance comparison of different gene fusion detection tools in simulated RNA-Seq data. Comparison of precision (**A**), recall (**B**), *F*-measure (**C**), and computing time (**D**) across 7 fusion detection tools, including Arriba, ChimeraScan, FusionCatcher, JAFFA, STAR-Fusion, STAR-SEQR, and Fcirc. In Fig. 4A-C, the black lines in the box (from top to bottom) represent upper quartile, median and lower quartile, respectively; the top and bottom black line represent upper extreme and lower extreme, respectively; the black dots represent data points.

We also evaluated the performance of all 7 algorithms for the simulated paired-end data including both linear and pooled linear/circular transcripts. Again, Fcirc achieved higher and more consistent precision (98.02%) than the other methods, with a high recall (85.64%) that was only second to Arriba (86.36%) (Fig. 4A and B, Suppl. Table 8). Fcirc also generated the highest and most consistent *F*-measures (0.91) in all of the simulated data, followed by Arriba (0.86) (Fig. 4C, Suppl. Table 9), and both Fcirc and Arriba required less computing time compared with the other methods (Fig. 4D). For the single-end (Suppl. Fig. 2) and paired-end data analysis (Suppl. Table 10), Fcirc required computing times ∼5 minutes or less depending on the RNA-Seq data settings.

### Evaluation of Fcirc performance to detect f-circRNAs in simulated RNA-Seq data

**Figure 5:**
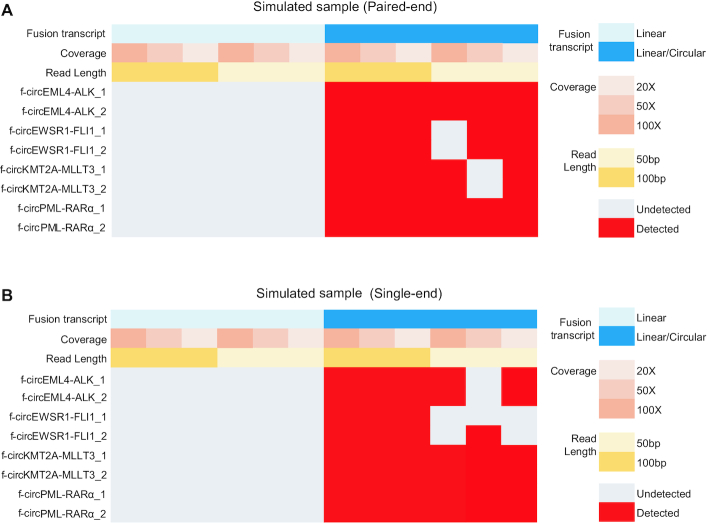
Identification of f-circRNAs in paired-end (A) and single-end (B) simulated RNA-Seq data. Fcirc was applied to detect f-circRNAs from 4 different fusion genes (*EML4*-*ALK, EWSR1*-*FLI1, KMT2A*-*MLLT3*, and *PML*-*RARα*) in simulated RNA-Seq datasets. Whereas the control dataset contains only linear fusion transcripts, the investigative dataset included pooled linear/circular fusion transcripts. Different sequencing coverages (20, 50, and 100×) and 2 read lengths (50 and 100 bp) were simulated.

**Figure 6.**
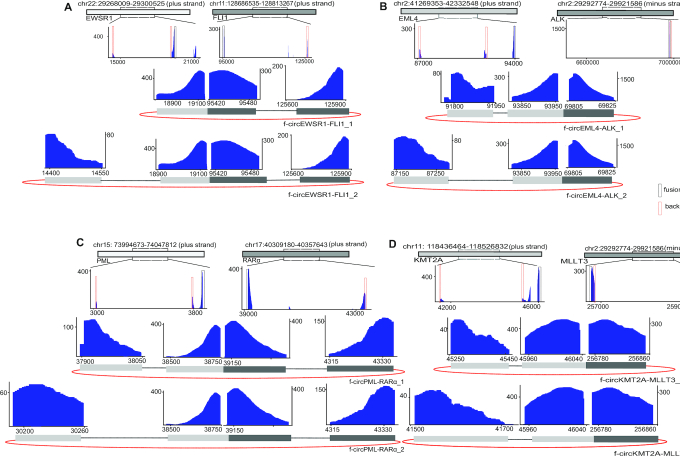
Visualization of f-circRNAs. The structure of fusion circular RNAs (n = 2 for each fusion gene) that were identified in the paired-end sample analysis with 100× coverage and a read length of 100 bp (Fig. 5A) is visualized for f-circ*EWSR1*-*FLI1* (**A**), f-circ*EML4-ALK* (**B**), f-circ*PML-RARα* (**C**), and f-circ*KMT2A*-*MLLT3* (**D**). The distribution of fusion-supporting reads on the fusion region (middle graph) and of f-circRNA-supporting reads on the back-spliced region (lower graph) are depicted.

To evaluate the ability of Fcirc to identify f-circRNA, we designed reads of 8 fusion circRNAs according to previous reports and plugged them into RNA-Seq data from normal pulmonary microvascular endothelial cells. We designed 2 types of RNA-Seq data: (i) a control dataset that contained only linear fusion transcripts and (ii) a dataset that contained pooled linear/circular fusion transcripts. Furthermore, single-end and paired-end RNA-Seq data, as well as different sequencing coverages (20, 50, 100×) and read lengths (50 and 100 bp), were simulated. In paired-end samples, Fcirc successfully detected all 8 types of f-circRNAs from RNA plugged with pooled linear/circular fusion transcripts, whereas—as expected—no f-circRNAs were detected when only the linear fusion transcripts were present (Fig. 5A). The Fcirc algorithm also showed high accuracy in simulated single-end samples (Fig. 5B). Overall, all f-circRNAs were identified in paired-end and single-end samples with a read length of 100 bp whereas more f-circRNAs were identified in the paired-end versus single-end samples with a read length of 50 bp and the same coverage. Eight f-circRNAs transcribed from 4 fusion genes that were identified in the paired-end sample dataset (100× coverage, 100 bp read length) are visualized in Fig. 6: (A) *EWSR1*-*FLI1*, (B) *EML4*-*ALK*, (C) *PML*-*RARα*, (D) *KMT2A*-*MLLT3*.

### Identification of f-circRNAs in actual RNA-Seq data

In the next step, we sought to identify f-circRNAs based on actual BioProject RNA-Seq data at the example of H3122 cells, an NSCLC cell line harbouring the *EML4*-*ALK* fusion (BioProject ID PRJNA350335) and of various acute leukemia samples with *PML*-*RARα* fusion gene (BioProject ID PRJNA315254). We applied the Fcirc algorithm to the PRJNA350335 dataset in order to detect linear and circular fusion transcripts. Fcirc successfully identified *EML4*-*ALK* fusions in all 9 H3122 samples at the specific previously reported fusion breakpoint (Suppl. Table 11; the number of supporting reads per cell line sample is indicated) [25]. We also successfully identified the previously reported *EML4*-*ALK* fusion-derived f-circRNA (Suppl. Table 12) [8, 35].

Next, we compared the performance of all 7 algorithms based on the PRJNA350335 dataset, which does not provide any information on the truly present fusion genes itself. Therefore, we defined fusions as TP results if they were detected by ≥4 tools with >10 supporting reads each, and then compared the performance of each method in detecting the presumably TP fusions. Comparable to the spike-in experiments, Fcirc (100%) achieved the highest precision compared to other methods (Suppl. Fig. 3A, Suppl. Table 13-1), whereas it lagged behind ChimeraScan (100%), STAR-Fusion (100%), and STAR-SEQR (100%) regarding the recall rate (Suppl. Fig. 3B, Suppl. Table 13-2). Fcirc also had higher *F*-measures (0.815) than most other tools, being second only to the STAR-SEQR algorithm (0.889) (Suppl. Fig. 3C, Suppl. Table 13-3). We detected the known *KMT2A*-*MLLT3* (*MLL*-*AF9*) and *PML*-*RARα* fusions in the BioProject dataset PRJNA315254 [7]. Supplemental Table 14 summarizes all fusion genes identified in datasets PRJNA350335 and PRJNA315254 and indicates the number of supporting reads for each individual fusion gene. Interestingly, different f-circRNA isoforms were detected for the *PML*-*RARα* fusion (Suppl. Table 12), with the total numbers of isoforms being dependent on the computational assumptions—e.g., 18 and 8 isoforms were detected in NB4 cells when the cut-off of read count numbers supporting f-circRNAs was changed to 1 or 2 (RNA-Seq data sample ID SRR3239817), respectively.

## Discussion

Fusion linear and circular transcripts (f-circRNA) are RNAs that are derived from rearranged genome translocations [1–6]. Even though the precise role of many of these RNAs remains elusive, it becomes increasingly evident that some of them are functional in gene expression regulation and therefore implicated in malignant transformation, cancer cell survival, and therapeutic resistance [7]. The example of crizotinib, an FDA-approved tyrosine kinase inhibitor for the treatment of *EML4-ALK*–rearranged NSCLC, shows that proteins encoded by fusion transcripts can also be harnessed as innovative drug targets [9]. This emphasizes the need for methods to accurately determine linear and f-circRNA profiles within cancer cells. Currently, numerous RNA-Seq datasets are publicly available that can be used to predict linear and f-circRNAs. However, it remains a significant challenge to detect specifically f-circRNA transcripts owing to their low frequency and low expression abundance within cancer cells. Furthermore, RNA-Seq data in general are hindered by heavy background noise, thus increasing the rate of FP results.

Therefore, we developed the Python-based pipeline “Fcirc” to overcome these drawbacks and to enable researchers to accurately identify and quantify linear and circular (f-circRNAs) fusion transcripts from RNA-Seq data. Fcirc differs from other published fusion detection tools such as Arriba [33], ChimeraScan [14], JAFFA [15], FusionCatcher [12], STAR-Fusion [13], and STAR-SEQR [34] by the fact that it requires information on already known gene fusions as reference to build the bipartite graph of gene pairs (Step 2 of the algorithm). Hence, the Fcirc algorithm—despite coming at the cost of losing the ability to identify new fusion genes—detects RNAs from known fusion events with higher specificity and lower FP rate. Fcirc accounts for the limitation of depending on known fusions by regularly updating information on newly emerging fusion genes from multiple databases (COSMIC, ChimerDB, TicDB, FARE-CAFÉ, FusionCancer). Users furthermore have the option to add their own fusion gene pairs of interest at their convenience.

In a benchmarking effort, we compared the performance of Fcirc with the 6 above-mentioned fusion detection tools (tool characteristics are summarized in Suppl. Table 3) on the basis of 3 different RNA-Seq data scenarios: (i) actual RNA-Seq data from synthetic spike-in experiments [21], (ii) simulated RNA-Seq data, and (iii) actual cancer cell–derived RNA-Seq data. The analyses in these 3 scenarios showed that Fcirc offers higher precision compared to all other algorithms (Figs 2A and 4A, Suppl. Fig. 3A), very high recall qualities (Figs 2B and 4B, Suppl. Fig. 3B), and *F*-measures (Figs 2C and 4C, Suppl. Fig. 3C), but also high numbers of fusion-supporting reads (Fig. 3) as well as reduced computing times (Figs 2D and 4D, Suppl. Fig. 2). Especially for the actual RNA-Seq dataset with synthetic fusion RNA spike-in (RNA-Seq Scenario 1), the problem of high FP rates became evident for some of the other tools because only 6.1% and 13.4% of fusion transcripts predicted by ChimeraScan and FusionCatcher were TPs, respectively, whereas Fcirc achieved a TP rate of 87.5% (Fig. 2A, Suppl. Table 5). Contrariwise, the recall rate was slightly lower for Fcirc compared with the other tools for the actual cancer cell–derived RNA-Seq datasets (RNA-Seq Scenario 3), which is likely due to presence of unknown fusions.

Fcirc furthermore detected f-circRNAs with high reliability and accuracy in simulated datasets with paired-end (Fig. 5A) and single-end samples (Fig. 5B) under different coverage and read length conditions as well as in actual cancer cell–derived RNA-Seq datasets (e.g., *EML4*-*ALK* and *PML*-*RARα*) (Suppl. Fig. 1). These results confirm previous reports on gene fusion events in multiple NSCLC and acute leukemia cell lines that were used for our analysis [7, 8, 35]. Interestingly, Fcirc identified ∼10 different f-circRNA transcripts for the *PML-RARα* fusion gene in NB4 leukemia cells (depending on the computational assumptions), which warrants further investigation and biological characterization (Suppl. Table 12).

In conclusion, our study provides an insightful comparison of different fusion detection tools and suggests Fcirc as a powerful tool to detect linear and circular RNA transcripts of known fusion genes with high specificity in RNA-Seq datasets. Fcirc's reduced computing time will expedite the analysis of very large data sets and therefore improve our future understanding of the impact of gene fusion–related transcripts on cancer biology.

## Availability of Supporting Source Code and Requirements

Project name: Fcirc: A Comprehensive Pipeline for the Exploration of Fusion, Linear and Circular RNAs

Project home page: https://github.com/WangHYLab/fcirc

Operating system(s): Ubuntu 16.04/18.04, MacOS

Programming language: Python

Other program requirements: hisat2, samtools, numpy, scipy, pysam

License: Massachusetts Institute of Technology (MIT, Cambridge, MA, USA)

Bio.tools id: biotools: Fcirc (https://bio.tools/Fcirc)

RRID:SCR_018090

## Availability of Supporting Data and Materials

Synthetic spike-in real RNA-Seq data were obtained from the SRA under the accession number SRP043081 [21]. Actual RNA-Seq data were obtained from the BioProject with accession IDs PRJNA350335 and PRJNA315254. Simulated RNA-Seq data were generated as described in the Methods section, and reference information of fusion transcripts and of f-circRNAs are presented in Supplementary Tables 2 and 3. Other data further supporting this work are openly available in the *GigaScience* repository, GigaDB [36].

## Additional Files

**Supplemental Figure S1**. Eight types of f-circRNAs from 4 fusion genes (*EML4*-*ALK, EWSR1*-*FLI1, KMT2A*-*MLLT3*, and *PML*-*RARα*) designed for simulated RNA-Seq datasets.

**Supplemental Figure S2**. Computing times of Fcirc in simulated single-end RNA-Seq data.

**Supplemental Figure S3**. Performance comparison of different gene fusion detection tools in actual RNA-Seq data (BioProject ID PRJNA350335).

**Supplemental Table S1**. Artificially designed fusion transcripts in simulated data (genome version: hg38).

**Supplemental Table S2**. Artificially designed f-circRNAs in simulated data (genome version: hg38).

**Supplemental Table S3**. Characteristics of fusion transcript detection tools.

**Supplemental Table S4**. Summary of running parameters of 7 fusion detection tools.

**Supplemental Table S5**. Precision and recall for synthetic RNA-Seq data.

**Supplemental Table S6**. *F*-measure for synthetic RNA-Seq data.

**Supplemental Table S7**. Computing time for synthetic data.

**Supplemental Table S8**. Precision and recall for simulated RNA-Seq data.

**Supplemental Table S9**. *F*-measure for simulated RNA-Seq data.

**Supplemental Table S10**. Computing time for paired-end simulated RNA-Seq data.

**Supplemental Table S11**. *EML4*-*ALK* fusions identified in actual cancer cell–derived RNA-Seq data (BioProject ID PRJNA350335).

**Supplemental Table S12**. F-circRNAs identified by Fcirc in actual cancer cell–derived RNA-Seq data.

**Supplemental Table S13**. Precision, recall, and *F*-measure for actual cancer cell–derived RNAseq data (BioProject ID PRJNA350335).

**Supplemental Table S14**. Fusion genes identified in actual cancer cell–derived RNA-Seq data (BioProject IDs PRJNA350335 and PRJNA315254).

giaa054_GIGA-D-19-00383_Original_Submission

giaa054_GIGA-D-19-00383_Revision_1

giaa054_GIGA-D-19-00383_Revision_2

giaa054_Response_to_Reviewer_Comments_Revision_1

giaa054_Response_to_Reviewer_Comments_Original_Submission

giaa054_Reviewer_1_Report_Original_Submission Wilfried Haerty -- 12/1/2019 Reviewed

giaa054_Reviewer_1_Report_Revision_1 Wilfried Haerty -- 3/9/2020 Reviewed

giaa054_Reviewer_2_Report_Original_Submission Pieter-Jan Volders -- 12/3/2019 Reviewed

giaa054_Supplemental_Files

## Abbreviations

bp: base pairs; circRNA: circular RNA; COSMIC: Catalogue of Somatic Mutations in Cancer; f-circRNA: fusion-circular ribonucleic acid; FDA: Food and Drug Administration; FN: false negative; FP: false positive; NCBI: National Center for Biotechnology Information; NSCLC: non–small cell lung cancer; PCC: paired chiastic clipping; RNA-Seq: RNA sequencing; SAM: Sequence Alignment/Map format; SRA: Sequence Read Archive; TP: true positive.

## Competing Interests

The authors declare that they have no competing interests.

## Authors’ Contributions

H.W. conceived the hypothesis. Z.C., H.X., Y.X., X.C., Y.D., and J.Z. designed and performed the pipeline workflow and the analyses. H.W., J.K., H.X., and Z.C. interpreted the results and wrote the manuscript.

## Funding

This work was supported by grants from the National Natural Science Foundation of China (31771469 and 31571363 to H.W.), a grant from the National Key Research and Development Program (2017YFC0908500 to H.W.), and a Mildred-Scheel postdoctoral fellowship from the German Cancer Aid Foundation(70111755 to J.K.).
