# Supplementary material for: Fcirc: A comprehensive pipeline for the exploration of fusion linear and circular RNAs
Source: Gigascience. 2020 May 29;9(6):giaa054. doi: 10.1093/gigascience/giaa054 (PMC7259471; doi:10.1093/gigascience/giaa054)
Supplement: giaa054_GIGA-D-19-00383_Original_Submission [file giaa054_giga-d-19-00383_original_submission.pdf]

GigaScience

Fcirc: A Comprehensive Pipeline for Exploration of Fusion Linear and Circular RNAs

--Manuscript Draft--

**Manuscript Number:**	GIGA-D-19-00383
**Full Title:**	Fcirc: A Comprehensive Pipeline for Exploration of Fusion Linear and Circular RNAs
**Article Type:**	Research
**Funding Information:**	National Key Research and Development Program (2017YFC0908500) — Dr. Haiyun Wang<br>National Natural Science Foundation of China (31771469, 31571363, 8157302) — Dr. Haiyun Wang
**Abstract:**	Background<br>Fusion transcripts, as chimeric ribonucleic acids (RNAs) encoded by fusion genes, play an important role in cancer onset and progression, with a molecular therapeutic target for specific cancers. Differing from linear-fusion transcripts, fusion circular RNAs (f-circRNAs) are special circular RNAs produced by fusion genes, which have recently been reported to have an oncogenic role in cancers. RNA sequencing (RNA-Seq) technologies along with existing bioinformatics approaches have enabled researchers to systematically identify fusion transcripts. However, finding f-circRNA in cells introduces a significant challenge for researchers due to the rare occurrence of f-circRNAs. The computational methods to specially identify f-circRNAs have not yet been fully explored.<br>Results<br>Here we have developed a python workflow, Fcirc, a newly comprehensive solution to effectively identify fusion transcripts and f-circRNAs from RNA-Seq data. Fcirc was applied in 3 types of RNA-Seq data sets, including synthetic spike-in real RNA-Seq data, simulated RNA-Seq data and real RNA-Seq data. Fcirc exhibited a significant advantage compared to existing methods in both accuracy and computing performance, empowering us to detect and understand the transcripts and circRNAs of fusion genes in cancers.<br>Conclusion<br>Fcirc is a newly comprehensive solution to effectively identify the fusion transcripts and f-circRNAs from RNA-Seq data, and helps to unravel the new f-circRNAs for the further investigation.
**Corresponding Author:**	Haiyun Wang, Ph.D<br>Tongji University<br>Shanghai, Shanghai CHINA
**Corresponding Author Secondary Information:**	
**Corresponding Author's Institution:**	Tongji University
**Corresponding Author's Secondary Institution:**	
**First Author:**	Zhaoqing Cai
**First Author Secondary Information:**	
**Order of Authors:**	Zhaoqing Cai<br>Hongzhang Xue<br>Yue Xu<br>Xiaojie Cheng<br>Yao Dai<br>Jie Zheng<br>Haiyun Wang, Ph.D

Powered by Editorial Manager® and ProduXion Manager® from Aries Systems Corporation

Order of Authors Secondary Information:	
Additional Information:	
Question	Response
Are you submitting this manuscript to a special series or article collection?	No
**Experimental design and statistics**<br><br>Full details of the experimental design and statistical methods used should be given in the Methods section, as detailed in our Minimum Standards Reporting Checklist. Information essential to interpreting the data presented should be made available in the figure legends.<br><br>Have you included all the information requested in your manuscript?	Yes
**Resources**<br><br>A description of all resources used, including antibodies, cell lines, animals and software tools, with enough information to allow them to be uniquely identified, should be included in the Methods section. Authors are strongly encouraged to cite Research Resource Identifiers (RRIDs) for antibodies, model organisms and tools, where possible.<br><br>Have you included the information requested as detailed in our Minimum Standards Reporting Checklist?	Yes
**Availability of data and materials**<br><br>All datasets and code on which the conclusions of the paper rely must be either included in your submission or deposited in publicly available repositories (where available and ethically appropriate), referencing such data using a unique identifier in the references and in the "Availability of Data and Materials" section of your manuscript.	Yes

Have you have met the above requirement as detailed in our Minimum Standards Reporting Checklist?	

Manuscript Click here to download Manuscript main_final.docx

Fcirc: A Comprehensive Pipeline for Exploration of Fusion Linear and Circular RNAs

Zhaoqing Cai[1,#], Hongzhang Xue[2,1#], Yue Xu[1], Xiaojie Cheng[1], Yao Dai[1], Jie Zheng[1], Haiyun Wang[1,*]

[1]*School of Life Sciences and Technology, Tongji University, Shanghai 200092, China*

[2]*School of Life Sciences and Biotechnology, Shanghai Jiao Tong University, Shanghai 200240, China*

E-mail: 1731473@tongji.edu.cn (Cai Z), xuehzh95@foxmail.com(Xue H), 1731490@tongji.edu.cn(Xu Y), siyecaodelvlanzi@163.com(Cheng X), daiyao0808@sina.com(Dai Y), 1931524@tongji.edu.cn (Zheng J), wanghaiyun@tongji.edu.cn(Wang H)

[#] Equal contribution.

[*] Corresponding author.

Abstract

Background

Fusion transcripts, as chimeric ribonucleic acids (RNAs) encoded by fusion genes, play an important role in cancer onset and progression, with a molecular therapeutic target for specific cancers. Differing from linear-fusion transcripts, fusion circular RNAs (f-circRNAs) are special circular RNAs produced by fusion genes, which have recently been reported to have an oncogenic role in cancers. RNA sequencing (RNA-Seq) technologies along with existing bioinformatics approaches have enabled researchers to systematically identify fusion transcripts. However, finding f-circRNA in cells introduces a significant challenge for researchers due to the rare occurrence of f-circRNAs. The computational methods to specially identify f-circRNAs have not yet been fully explored.

Results

Here we have developed a python workflow, Fcirc, a newly comprehensive solution to effectively identify fusion transcripts and f-circRNAs from RNA-Seq data. Fcirc was applied in 3 types of RNA-Seq data sets, including synthetic spike-in real RNA-Seq data, simulated RNA-Seq data and real RNA-Seq data. Fcirc exhibited a significant advantage compared to existing methods in both accuracy and computing performance, empowering us to detect and understand the transcripts and circRNAs of fusion genes in cancers.

Conclusion

Fcirc is a newly comprehensive solution to effectively identify the fusion transcripts

and f-circRNAs from RNA-Seq data, and helps to unravel the new f-circRNAs for the further investigation.

Keywords: Fcirc; Fusion linear RNA; Fusion circRNA; Performance benchmarks

Background

In the cancer genome, the presence of a large number of harmful gene mutations, gene rearrangements and chromosomal fragile sites leads to the occurrence of fusion genes [1-6]. Fusion circular RNAs (f-circRNAs) are special circular RNAs (circRNAs) produced by fusion genes that help cellular transformation, promote cell viability, confer therapeutic resistance to cells, and have tumour-promoting properties [7]. In addition, f-circRNAs also have the potential to be novel liquid biopsy biomarkers [8]. Advances in high-throughput RNA-Seq data enable the detection of many fusions [9-14], as well as circRNAs [15-17]. However, novel fusion detection tools yield a high false discovery rate [18], and current bioinformatics methods cannot be used to fully identify f-circRNAs [19].

Here we present Fcirc (Fig 1, see methods), a comprehensive workflow that explores linear RNAs and circRNAs of fusions. Differing from recently published fusion detection methods such as ChimeraScan [13], JAFFA [14], FusionCatcher [11] and STAR-Fusion [12], Fcirc preferentially detects RNAs coming from known fusion events, resulting in competing computing speed and reduced false positives. Moreover, Fcirc is able to detect the circular transcripts of fusion genes. Therefore, Fcirc is a

no-cost solution to effectively utilize public RNA-Seq data to quickly identify fusion transcripts and f-circRNAs.

Materials and methods

The pipeline of Fcirc

The pipeline of Fcirc executes analysis by following five major steps (Fig 1). As input, Fcirc requires single-end or paired-end RNA-Seq data in FASTQ format. Either raw data or clean data (e.g. cutting the adapter and trimming poor-quality) is acceptable.

1) Dropping the aligned reads

When reads are aligned to a reference transcriptome with HISAT2 [20], the scoring parameters need to be strict to make reads with perfect matches and align few mismatches. We increased the maximum and minimum penalty for mismatch (--mp 12, 4), the maximum and minimum read gap open and extension penalty (--rdg 10, 6), the maximum and minimum reference gap opening and extension penalty (--rfg 10, 6), and change the soft matching parameter (--no-softclip). After the first alignment was completed, the aligned reads were dropped by samtools [21] and the unaligned reads were kept for further analysis. For single-end RNA-Seq data, reads with a FLAG value of 4 in the Sequence Alignment / Map format (SAM) file were selected (-f 4) and converted into files in FASTQ format. For paired-end RNA-Seq data, reads without a FLAG value of 2 in the SAM file were selected (-F 2), meaning that either segment of a read was unaligned. Then, they were converted into a file in FASTQ format.

2) Building a bipartite graph of gene pairs of known fusions

Gene pairs of known fusions were manually curated from multiple databases, including COSMIC [22], ChimerDB [23], TicDB [24], FARE-CAFE [25] and FusionCancer [26]. A bipartite graph (or bigraph) is a graph whose vertices can be divided into two disjoint and independent sets **U** and **V** such that every edge connects a vertex in **U** to one in **V**. Genes were vertices and fusion events were edges. The genes involved in the fusion event did not form a ring of odd vertices, so a bipartite graph of known fusion gene-pairs could be built. To reduce the computational and time complexity of searching reads that span multiple genes, genes involved in the fusion event were divided into two independent sets according to the bipartite graph. For example, *EML4-ALK* and *NPM1-ALK* have been reported to be fusion genes so that *EML4* and *NPM1* were in the same set while *ALK* was in the rest of the data set. The gene sequences of known fusions were downloaded from the database ensemble [27].

3) Selecting fusion-related reads

The unaligned reads in the first step were re-aligned to two sets of fusion gene sequences with low penalty, independently. We decreased the maximum and minimum penalty for soft-clipping (--sp 1, 1) and minimum alignment score (--score-min L, 0, -0.8). Other scoring parameters were set as the default. After this re-alignment, reads with part of sequences aligned to fusion genes were selected. For single-end RNA-Seq data, reads without a FLAG value of 4 in the SAM file were selected (-F 4). For paired-end RNA-Seq data, reads with a FLAG value of 4, not 8, or

8, not 4 or 12 in the SAM file were selected (-f 4 -F 8 or -F 4 -f 8 or -f 12), which means that at least one segment of a read were aligned. The reads with paired chiastic clipping (PCC) signal were defined as fusion-related reads. For instance, if a segment of a read was aligned to *EML4* with the same FLAG and CIGAR 40S60M values while it was aligned to *ALK* with FLAG 4 and CIGAR 40M60S, it suggested that one part of a segment was from *EML4* and the rest was from *ALK* in the same strand.

4) Reconstructing and verifying the fusion genes

Next, the fusion-related reads determined the fusion breakpoint. On the assumption that fusion-related reads were more likely to cover the fusion breakpoint, we preferred the junction covered by the most fusion-related reads as the fusion breakpoint, which is simply inferred by the majority of junction-supported reads. Then, the sequences of the fusion gene were reconstructed by this predicted fusion breakpoint.

After the reconstruction of the fusion gene, the alignment of reads was recalibrated by re-aligning reads to the reconstructed fusion gene with a low penalty. We assumed that the fusion-related reads uniformly covered the fusion breakpoint. To evaluate if the fusion-related reads uniformly covered the fusion breakpoint, the fusion-related reads were split into two fragments: the left fragments and right fragments around the breakpoint. The Wilcoxon Sign Rank Test was used to evaluate the distribution of reads, by comparing the length of the left fragments with one of the right fragments. Only a breakpoint with the same distribution of read length distribution at its flanking region was determined as the true breakpoint for the fusion genes.

5) Transforming back-spliced reads

The circular RNAs of fusion genes were detected by searching for back-spliced reads. To improve alignment between back-spliced reads with the reconstructed fusion gene, we changed the order of aligned and unaligned segments of some back-spliced reads to transform back-spliced reads to forward-spliced reads. The transformed reads were re-aligned to the reconstructed fusion gene to evaluate whether they were truly back-spliced. Those reads covering a back-spliced junction indicated that they were from f-circRNA.

As output, Fcirc provides tables of fusion transcripts and f-circRNAs. To help visualize the reads with their distribution on fusion, Fcirc also provides the fusion-related reads in SAM format.

Performance benchmarks and evaluation criteria

Fcirc and four currently published fusion detection methods, including ChimeraScan v0.4.5 [13], FusionCatcher v1.0.0 [11], JAFFA v1.0.9 [14] and STAR-Fusion v1.5.0 [12], were applied to the synthetic spike-in real RNA-Seq data and simulated RNA-Seq data. To accurately evaluate these tools, we defined that 1) the number of fusion-supporting reads must be at least 3, and 2) read-through transcripts (two genes located on the same chromosome less than 100,000 bp apart) are removed. The computational efficiency of the tools was evaluated by using several criteria. The primary benchmark was precision, recall and F-measure.

$$precision = TP/(TP + FP)$$

$$recall = TP/(TP + FN)$$

$$F - measure = precision * recall * 2/(precison + recall)$$

Where TP, FP and FN represent the true positives, false positives and false negatives, respectively. The F-measure simultaneously considers the effect of precision and recall.

We also evaluated the number of supporting reads identified by the different tools, that reflected the ability to robustly detect the fusion. The last benchmark was the computing time of the different tools.

Datasets

Synthetic spike-in real RNA-Seq data

The synthetic spike-in RNA-Seq data consisted of 9 synthetic poly-adenylated RNA transcripts that corresponded to reported cancer fusion genes, including *EWSR1-ATF1*, *TMPRSS2-ETV1*, *EWSR1-FLI1*, *NTRK3-ETV6*, *CD74-ROS1*, *HOOK3-RET*, *EML4-ALK*, *AKAP9-BRAF*, and *BRD4-NUTM1* [28]. These synthetic gene fusion RNA constructs were spiked at 10 different concentrations into total RNA, with 2 replicates for each RNA-Seq sample. In total, there were 20 RNA-Seq datasets included in these data.

Simulated RNA-Seq data

The simulator art_illumina function in ART [29] was applied to generate simulated RNA-Seq data. Using the RNA-Seq reads in normal pulmonary microvascular endothelial cells from the National Center for Biotechnology Information (NCBI) Sequence Read Archive (SRA) database SRR349695 [30] as the background, we plugged the simulated fusion reads into the background reads. Two types of fusion

reads were designed, with the first one from the linear transcripts and the second one from the linear and circular pooled transcripts. A total of 47 fusions (Supplemental_Tab_S10) were selected from the Catalogue Of Somatic Mutations In Cancer (COSMIC) database [22], and the linear fusion reads were artificially generated based on the breakpoint information by joining the upstream transcript fragment and downstream transcript fragment. Eight fusion circRNAs (Supplemental_Fig_S2, Supplemental_Tab_S11) were generated according to previous reports of f-circRNAs [7, 31].

To simulate more linear fusion transcripts than circular transcripts at a gene locus, the amount of linear fusion reads plugged into the background reads was 2.5 times as many as that of circular fusion reads. Different sequencing coverage including 20X, 50X, 100X, each with two read lengths of 50 bp and 100 bp were designed in the simulated data.

Real data

Real data for f-circRNA identification were obtained from the BioProject with accession numbers PRJNA350335 and PRJNA315254. The data set PRJNA350335 includes 9 cell lines of H3122 harbouring the *EML4-ALK* fusion gene [32]. The data set PRJNA315254 includes 9 acute leukaemia samples with 3 NB4 cell lines, 2 THP1 cell lines and 4 primary patients harbouring the *PML-RARα* fusion gene [7].

Results

Evaluation of gene fusions in synthetic spike-in real and simulated RNA-Seq data

In synthetic RNA-Seq data, 9 synthetic cancer-associated fusion genes, including *EWSR1-ATF1*, *TMPRSS2-ETV1*, *EWSR1-FLI1*, *NTRK3-ETV6*, *CD74-ROS1*, *HOOK3-RET*, *EML4-ALK*, *AKAP9-BRAF*, and *BRD4-NUTM1*, were spiked-in (see methods). Compared with the other tools, Fcirc (Fig 2A, Supplemental_Tab_S1) achieved the highest precision (73.50%), followed by JAFFA (63.25%) and STAR-Fusion (47.28%). ChimeraScan (15.54%) and FusionCatcher (13.47%) obtained low precision values, suggesting a high risk of false positives for their predictions. In addition, FusionCatcher (87.78%), Fcirc (86.11%), and STAR-Fusion (77.22%) achieved higher recall than JAFFA (62.78%) and ChimeraScan (52.22%) (Fig 2B, Supplemental_Tab_S1). Fcirc had greater F-measure values (0.78), indicating its better performance for balancing precision and recall **(**Fig 2C, Supplemental_Tab_S2). In addition, Fcirc (red squares) required the least computing time with approximately 2-4 hours, followed by STAR-Fusion (Fig 2D, Supplemental_Tab_S3). The computational environment was based on Ubuntu Linux with Intel Xeon E5-2620 v4 CPU@ 2.10GHz, and four CPU cores were used for each tool.

There were 10 different concentrations for 9 types of synthetic gene fusion RNA, with 2 replicates for each RNA-Seq dataset. We calculated the number of fusion-supporting reads, the junction reads for identifying gene fusions, for the different tools. The results (Fig 3) showed that Fcirc (red squares) identified the

highest number of supporting reads under different concentrations, at which the synthetic gene fusion RNA constructs were spiked into total RNA. Moreover, with increasing concentrations, the number of identified supporting reads of gene fusions grew rapidly.

We also evaluated the performance of five tools in the simulated paired-end data (see methods). In these data, Fcirc (Fig 4A, B, Supplemental_Tab_S4) achieved higher precision (93.99%) and recall (90.60%) than the other tools. Similarly, with real data, although ChimeraScan achieved higher precision (95.76%), its recall rates were considerably lower (48.05%). The highest F-measures (Fig 4C, Supplemental_Tab_S5) were generated by Fcirc (0.92) in all of simulated data, followed by FusionCatcher (0.81). Fcirc still exhibited distinct advantages of computing time in simulated paired-end data (Fig 4D, Supplemental_Tab_S6). Among these tools, JAFFA required the longest computing time for nearly all the data. Furthermore, under fixed coverage, the increased read length prolonged the running time for ChimeraScan, FusionCatcher and STAR-Fusion, but not for Fcirc (Supplemental_Tab_S6). As for computing time in single-end data, the computing time of Fcirc was in less than 5 minutes (Supplemental_Fig_S1).

Evaluation of f-circRNAs in simulated RNA-Seq data

To evaluate the ability of the tools to identify f-circRNA, we designed reads of 8 fusion circRNAs according to previous reports and plugged them into RNA-Seq data from normal pulmonary microvascular endothelial cells (see methods). We designed two types of RNA-Seq data, the first one was a control, containing only the linear

fusion transcripts, and the second one contained the linear/circular fusion transcripts. Different sequencing coverage and read lengths were considered in the simulated data. In simulated paired-end samples (Fig 5A), Fcirc successfully detected 8 types of f-circRNAs from RNA-Seq data containing linear/circular fusion transcripts. As expected, no f-circRNAs were detected from the RNA-Seq data only containing linear fusion transcripts. Fcirc also worked well in simulated single-end samples (Fig 5B). Moreover, all f-circRNAs were identified when the read length was 100 bp, both in paired-end samples and single-end samples. More f-circRNAs were identified in the paired-end samples than in single-end samples when the read length was 50 bp under the same coverage. Eight types of f-circRNAs from 4 fusion genes (*EML4-ALK*, *EWSR1-FLI1*, *KMT2A-MLLT3*, *PML-RARα*) identified in the paired-end sample with 100X coverage and read length of 100 bp were visualized (Fig 6).

Identification of f-circRNAs in real data

Next, we identified f-circRNAs based on real data obtained from the BioProject with accession numbers PRJNA350335 and PRJNA315254. PRJNA350335 includes 9 cell lines of H3122 harbouring the *EML4-ALK* fusion gene, and PRJNA315254 includes 9 acute leukaemia samples with 3 NB4 cell lines, 2 THP1 cell lines and 4 primary patients harbouring the *PML-RARα* fusion gene.

In the data set PRJNA350335, we applied Fcirc to detect the linear and circular fusion transcripts. Fcirc successfully identified *EML4-ALK* fusion at a specific fusion breakpoint in 9 H3122 cell lines with the amount of supporting reads (Supplemental_Tab_S7), which is consistent with the previous report [32] and what

STAR-Fusion and ChimeraScan identified. In addition to the capability of identifying fusion linear transcripts such as many fusion detection tools, Fcirc is newly designed to detect f-circRNA as well by detecting the back-spliced reads in a fusion gene. As we expected, we successfully identified the previously reported f-circRNA *EML4-ALK* [8, 33] (Supplemental_Tab_S8).

In the data set PRJNA315254, we detected the *KMT2A-MLLT3* (*MLL-AF9*) fusion and *PML-RARα* fusion with an amount of supporting reads (Supplemental_Tab_S9), which was consistent with a previous study [7]. Interestingly, the different types of f-circRNA isoforms for the *PML-RARα* fusion were detected in this dataset (Supplemental_Tab_S8). When the cutoff of f-circRNA-supporting reads counts were 1 or 2, 18 or 8 f-circRNA isoforms from the PML-RARα fusion were respectively detected in the RNA-Seq data of the NB4 cell line SRR3239817. PML-RARα f-circRNAs have been reported in the study of Guarnerio et al [7]. All fusion genes identified in the data sets PRJNA350335 and PRJNA315254 are shown in Supplemental_Tab_S9.

Discussion

Fusion linear and circular transcripts can be involved in a variety of cancerous transformations, indicating their potential diagnostic and therapeutic implications. Currently, a large amount of RNA-Seq data is widely available, making it feasible to efficiently take advantage of these data for f-circRNA predictions. It is a significant challenge to find f-circRNA in cells due to its rare occurrence. Here, we have

developed a python workflow, Fcirc, a newly comprehensive solution to effectively identify the fusion transcripts and f-circRNAs from public RNA-Seq data. In this study, we compared the performance of Fcirc with four fusion detection tools in synthetic spike-in real and simulated data sets. Fcirc achieved advantages in precision, recall, fusion-supporting reads number and computing time. Our results provide an insightful comparison of different fusion detection tools and indicate that Fcirc is a reliable tool for fusion detection. Moreover, Fcirc successfully detected all f-circRNAs in simulated data and the f-circRNAs (*EML4-ALK*, *PML-RARα*) reported by previous studies[7, 8, 33] in real data. In the human promyelocytic leukaemia cell line NB4, approximately 10 f-circRNA transcripts were identified, which is worth further investigation.

Our pipeline exhibits significant advantages compared to existing methods. RNA-Seq data suffers from heavy background noise leading to unexpected false positive detection. For example, in the synthetic data set, only 15.54% of the fusion transcripts predicted by ChimeraScan and 13.47% of the fusion transcripts predicted by FusionCatcher are true positives. Regarding this issue, Fcirc uses known fusions as a reference to build a bipartite graph of gene pairs, dramatically decreasing false positives. Moreover, this also greatly reduces the computing time, and Fcirc computed in the simulated RNA-Seq data in minutes, significantly expediting performance speed and outperforming the current tools.

Though at the cost of losing the ability to identify new fusion genes not reported by fusion gene databases, our method updates known fusion genes collected from the

multiple databases in a timely manner, and the user can add their own fusion genes by using optional input. In conclusion, Fcirc will help us better detect and understand the transcripts and circRNAs of fusion genes in cancers.

Availability of Supporting Source Code and Requirements

Project name: Fcirc: A Comprehensive Pipeline for Exploration of Fusion, Linear and Circular RNAs

Project home page: https://github.com/WangHYLab/fcirc

Operating system(s): Ubuntu 16.04/18.04, MacOS

Programming language: Python

Other requirements: hisat2, samtools, numpy, scipy, pysam

License: MIT

Availability of Supporting Data and Materials

Synthetic spike-in real RNA-Seq data was obtained from the Short Read Archive under accession number SRP043081. Real data was obtained from the BioProject with accession numbers PRJNA350335 and PRJNA315254. Simulated RNA-Seq data was obtained by the method in Method section, and reference information of fusion transcripts and f-circRNAs were showed in Additional files section.

Abbreviations

RNA: ribonucleic acid; RNA-Seq: ribonucleic acid sequencing; f-circRNA: fusion-circular ribonucleic acid; circRNA: circular ribonucleic acid; SAM: Sequence Alignment/Map format; PCC: paired chiastic clipping; TP: true positive; FP: false positive; FN: false negative; SRA: Sequence Read Archive; NCBI: National Center for Biotechnology Information; COSMIC: Catalogue Of Somatic Mutations In Cancer

Competing interests

The authors declare no competing interests.

Authors' contributions

HW conceived the hypothesis. ZC, HX, XC, YD, and JZ designed and performed the pipeline and analysis. HW, HX and ZC wrote the manuscript.

Acknowledgements

This work was supported by grants from the National Natural Science Foundation of China (31771469, 31571363, and 81573023 to HW), and a grant from the National Key Research and Development Program (2017YFC0908500 to HW).

References

1. Lebeau MM,Rowley JD. Cancer biology: Heritable fragile sites in cancer. Nature. 1984;308

5960:607-8.

2. Stratton MR, Campbell PJ,Futreal PA. The cancer genome. Nature. 2009;458 7239:719-24.

3. Huebner K. Molecular biology: DNA fragility put into context. Nature. 2011;470 7332:46-7.

4. Coquelle A, Toledo F, Stern S, Bieth A,Debatisse M. A New Role for Hypoxia in Tumor Progression: Induction of Fragile Site Triggering Genomic Rearrangements and Formation of Complex DMs and HSRs. Molecular Cell. 1998;2 2:259-65.

5. Novo FJ,Vizmanos JL. Chromosome translocations in cancer: computational evidence for the random generation of double-strand breaks. Trends in Genetics. 2006;22 4:193-6.

6. Imielinski M,Ladanyi M. Fusion oncogenes—genetic musical chairs. Science. 2018;361 6405:848-9.

7. Guarnerio J, Bezzi M, Jeong JC, Paffenholz SV, Berry K, Naldini MM, et al. Oncogenic Role of Fusion-circRNAs Derived from Cancer-Associated Chromosomal Translocations. Cell. 2016;165 2:289-302. doi:10.1016/j.cell.2016.03.020.

8. Tan S, Gou Q, Pu W, Guo C, Yang Y, Wu K, et al. Circular RNA F-circEA produced from EML4-ALK fusion gene as a novel liquid biopsy biomarker for non-small cell lung cancer. Cell research. 2018;28 6:693-5. doi:10.1038/s41422-018-0033-7.

9. Wang K, Singh D, Zeng Z, Coleman SJ, Huang Y, Savich GL, et al. MapSplice: accurate mapping of RNA-seq reads for splice junction discovery. Nucleic Acids Res. 2010;38 18:e178. doi:10.1093/nar/gkq622.

10. Kim D,Salzberg SL. TopHat-Fusion: an algorithm for discovery of novel fusion transcripts. Genome Biol. 2011;12 8:R72. doi:10.1186/gb-2011-12-8-r72.

11. Nicorici D, Şatalan M, Edgren H, Kangaspeska S, Murumägi A, Kallioniemi O, et al. FusionCatcher – a tool for finding somatic fusion genes in paired-end RNA-sequencing data. bioRxiv. 2014:011650. doi:10.1101/011650.

12. Haas BJ, Dobin A, Stransky N, Li B, Yang X, Tickle T, et al. STAR-Fusion: Fast and Accurate Fusion Transcript Detection from RNA-Seq. bioRxiv. 2017:120295. doi:10.1101/120295.

13. Iyer MK, Chinnaiyan AM,Maher CA. ChimeraScan: a tool for identifying chimeric transcription in sequencing data. Bioinformatics. 2011;27 20:2903-4. doi:10.1093/bioinformatics/btr467.

14. Davidson NM, Majewski IJ,Oshlack A. JAFFA: High sensitivity transcriptome-focused fusion gene detection. Genome Med. 2015;7 1:43. doi:10.1186/s13073-015-0167-x.

15. Gao Y, Wang J,Zhao F. CIRI: an efficient and unbiased algorithm for de novo circular RNA identification. Genome Biol. 2015;16:4. doi:10.1186/s13059-014-0571-3.

16. Szabo L, Morey R, Palpant NJ, Wang PL, Afari N, Jiang C, et al. Statistically based splicing detection reveals neural enrichment and tissue-specific induction of circular RNA during human fetal development. Genome Biol. 2015;16:126. doi:10.1186/s13059-015-0690-5.

17. Song X, Zhang N, Han P, Moon BS, Lai RK, Wang K, et al. Circular RNA profile in gliomas revealed by identification tool UROBORUS. Nucleic Acids Res. 2016;44 9:e87. doi:10.1093/nar/gkw075.

18. Kumar S, Vo AD, Qin F,Li H. Comparative assessment of methods for the fusion transcripts detection from RNA-Seq data. Sci Rep. 2016;6:21597. doi:10.1038/srep21597.

19. Zeng X, Lin W, Guo M,Zou Q. A comprehensive overview and evaluation of circular RNA detection tools. PLOS Computational Biology. 2017;13 6:e1005420. doi:10.1371/journal.pcbi.1005420.

20. Kim D, Langmead B,Salzberg SL. HISAT: a fast spliced aligner with low memory requirements. Nature Methods. 2015;12:357. doi:10.1038/nmeth.3317.

21. Li H, Handsaker B, Wysoker A, Fennell T, Ruan J, Homer N, et al. The Sequence Alignment/Map format and SAMtools. Bioinformatics. 2009;25 16:2078-9. doi:10.1093/bioinformatics/btp352.

22. Forbes SA, Beare D, Boutselakis H, Bamford S, Bindal N, Tate J, et al. COSMIC: somatic cancer genetics at high-resolution. Nucleic Acids Res. 2017;45 D1:D777-d83. doi:10.1093/nar/gkw1121.

23. Lee M, Lee K, Yu N, Jang I, Choi I, Kim P, et al. ChimerDB 3.0: an enhanced database for fusion genes from cancer transcriptome and literature data mining. Nucleic Acids Research. 2017;45 Database issue:D784-D9.

24. Novo FJ, Mendíbil IOD,Vizmanos JL. TICdb: a collection of gene-mapped translocation breakpoints in cancer. Bmc Genomics. 2007;8 1:33.

25. Korla PK, Cheng J, Huang CH, Tsai JJ, Liu YH, Kurubanjerdjit N, et al. FARE-CAFE: a database of functional and regulatory elements of cancer-associated fusion events. Database (Oxford). 2015;2015 doi:10.1093/database/bav086.

26. Wang Y, Wu N, Liu J, Wu Z,Dong D. FusionCancer: a database of cancer fusion genes derived from RNA-seq data. Diagnostic Pathology. 2015;10 1:131.

27. Zerbino DR, Achuthan P, Akanni W, Amode MR, Barrell D, Bhai J, et al. Ensembl 2018. Nucleic Acids Research. 2018;46 Database issue:D754.

28. Tembe WD, Pond SJ, Legendre C, Chuang HY, Liang WS, Kim NE, et al. Open-access synthetic spike-in mRNA-seq data for cancer gene fusions. BMC Genomics. 2014;15:824. doi:10.1186/1471-2164-15-824.

29. Huang W, Li L, Myers JR,Marth GT. ART: a next-generation sequencing read simulator. Bioinformatics. 2012;28 4:593-4. doi:10.1093/bioinformatics/btr708.

30. Zhang LQ, Cheranova D, Gibson M, Ding S, Heruth DP, Fang D, et al. RNA-seq reveals novel transcriptome of genes and their isoforms in human pulmonary microvascular endothelial cells treated with thrombin. PloS one. 2012;7 2:e31229. doi:10.1371/journal.pone.0031229.

31. Tan S, Gou Q, Pu W, Guo C, Yang Y, Wu K, et al. Circular RNA F-circEA produced from EML4-ALK fusion gene as a novel liquid biopsy biomarker for non-small cell lung cancer. Cell Research. 2018;28 6:693-5. doi:10.1038/s41422-018-0033-7.

32. Rusan M, Li K, Li Y, Christensen CL, Abraham BJ, Kwiatkowski N, et al. Suppression of Adaptive Responses to Targeted Cancer Therapy by Transcriptional Repression. Cancer Discov. 2018;8 1:59-73. doi:10.1158/2159-8290.cd-17-0461.

33. Tan S, Sun D, Pu W, Gou Q, Guo C, Gong Y, et al. Circular RNA F-circEA-2a derived from EML4-ALK fusion gene promotes cell migration and invasion in non-small cell lung cancer. Mol Cancer. 2018;17 1:138. doi:10.1186/s12943-018-0887-9.

Figure legends

Figure 1. Fcirc pipeline for exploring linear and circular RNAs of known fusions

Five main steps of Fcirc, including dropping aligned reads, building a bipartite graph of gene pairs of known fusions, selecting fusion-related reads, reconstructing and verifying the fusion genes and transforming back-spliced reads.

Figure 2. Performance comparison of the different gene fusion detection tools in synthetic spike-in real RNA-Seq data

Comparison of precision (**A**), recall (**B**), F-measure (**C**) and computing time (**D**) across five fusion detection tools, including ChimeraScan (khaki triangles), FusionCatcher (green triangles), JAFFA (blue triangles), STAR-Fusion (purple triangles) and Fcirc (red squares).

Figure 3. Fusion-supporting reads identified by the different gene fusion detection tools in synthetic spike-in real RNA-Seq data

The amount of fusion-supporting reads in 9 fusions (*AKAP9-BRAF*, *BRD4-NUTM1*, *CD74-ROS1*, *EML4-ALK*, *EWSR1-ATF1*, *EWSR1-FLI1*, *HOOK3-RET*, *NTRK3-ETV6*, and *TMPRSS2-ETV1*) identified by ChimeraScan, FusionCatcher, JAFFA, STAR-Fusion and Fcirc in synthetic spike-in real RNA-Seq data. Each fusion has two replicates.

Figure 4. Performance comparison of the different gene fusion detection tools in simulated RNA-Seq data

Comparison of precision (**A**), recall (**B**), F-measure(**C**), and computing time (**D**) across five fusion detection tools, including ChimeraScan (khaki triangles), FusionCatcher (green triangles), JAFFA (blue triangles), STAR-Fusion (purple triangles) and Fcirc (red squares).

Figure 5. The identification of f-circRNAs in paired-end (A) and single-end (B) simulated RNA-Seq data

Eight types of f-circRNAs from 4 fusion genes (*EML4-ALK*, *EWSR1-FLI1*, *KMT2A-MLLT3*, and *PML-RARα*) were included in simulated RNA-Seq data. Two types of RNA-Seq data, with the first one as a control only containing the linear fusion transcripts and the second one containing the linear/circular fusion transcripts, were designed. Different sequencing coverage including 20X, 50X, 100X, each with two read length of 50bp and 100bp, were designed.

Figure 6. Visualization of f-circRNAs

f-circ*EWSR1-FLI1*(**A**), f-circ*EML4-ALK*(**B**), f-circ*PML-RARα*(**C**), f-circ*KMT2A-MLLT3* (**D**), identified from simulated RNA-Seq data. These f-circRNAs were identified in the paired-end samples with 100X coverage and read length of 100 bp. For each fusion gene, there are 2 f-circRNAs were detected. The distribution of fusion-supporting reads and f-circRNA-supporting reads are shown on the fusion region and back-spliced region respectively.

Additional files

Supplemental_Fig_S1. Computing time of Fcirc in simulated single-end RNA-Seq data.

Supplemental_Fig_S2. Eight types of f-circRNAs from 4 fusion genes (*EML4-ALK, EWSR1-FLI1, KMT2A-MLLT3,* and *PML-RARα*) designed in the simulated RNA-Seq data.

Supplemental_Tab_S1. Precision and recall for synthetic RNA-Seq data.

Supplemental_Tab_S2. F-measure for synthetic RNA-Seq data.

Supplemental_Tab_S3. Computing time for synthetic data.

Supplemental_Tab_S4. Precision and recall for simulated RNA-Seq data.

Supplemental_Tab_S5. F-measure for simulated RNA-Seq data.

Supplemental_Tab_S6. Computing time of paired-end simulated data.

Supplemental_Tab_S7. *EML4-ALK* fusions identified in real data PRJNA350335.

Supplemental_Tab_S8. F-circRNAs identified by Fcirc in real data.

Supplemental_Tab_S9. Fusion genes identified in real data PRJNA350335 and PRJNA315254.

Supplemental_Tab_S10. Artificially designed fusion transcripts in simulated data (genome version: hg38).

Supplemental_Tab_S11. Artificially designed f-circRNAs in simulated data (genome version: hg38).

Fig 1.

Click here to download Figure Fig 1. Fcirc pipeline.pdf

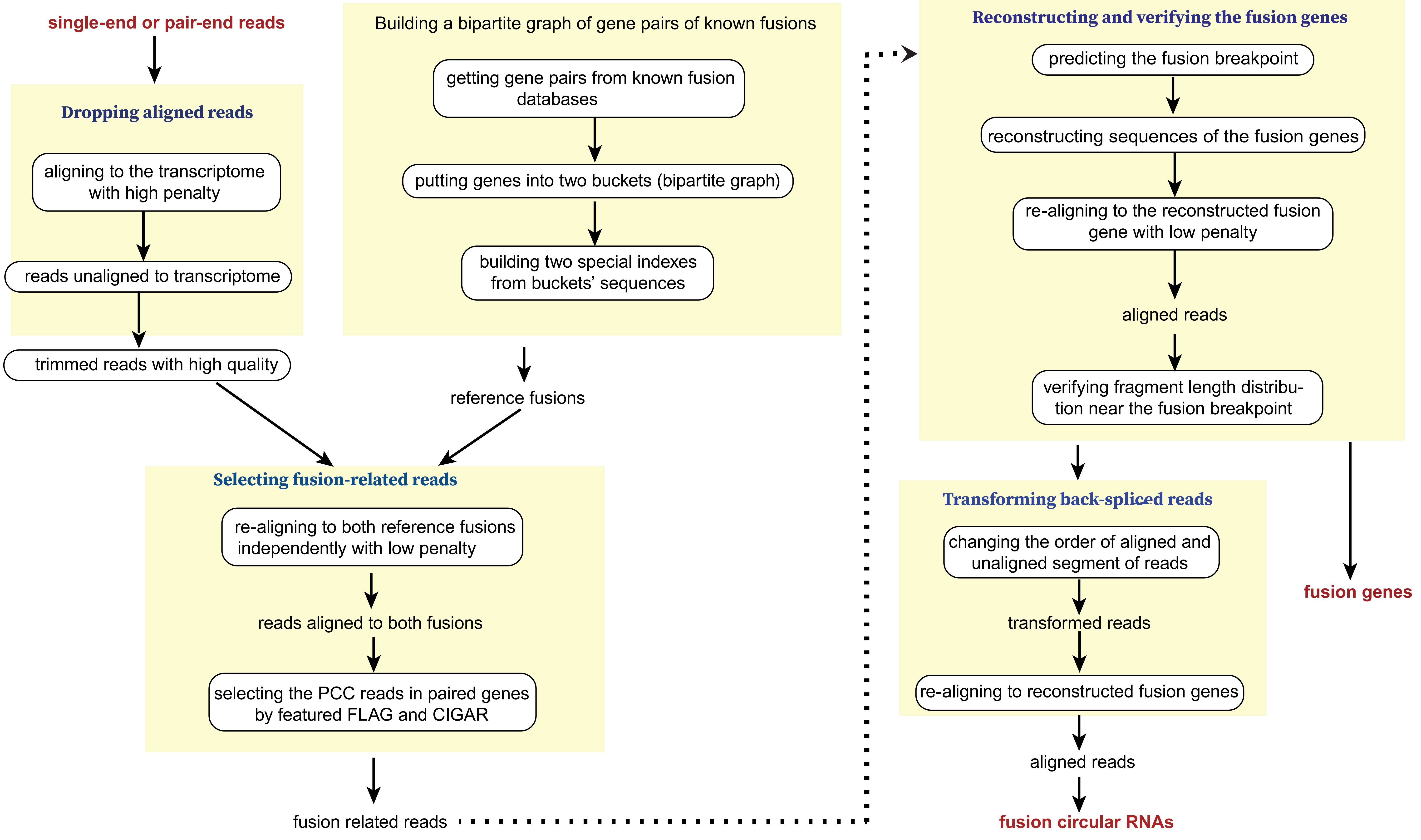

Fig. 2

Click here to download Figure Fig 2. Evaluation in Synthetic RNA-Seq data.pdf

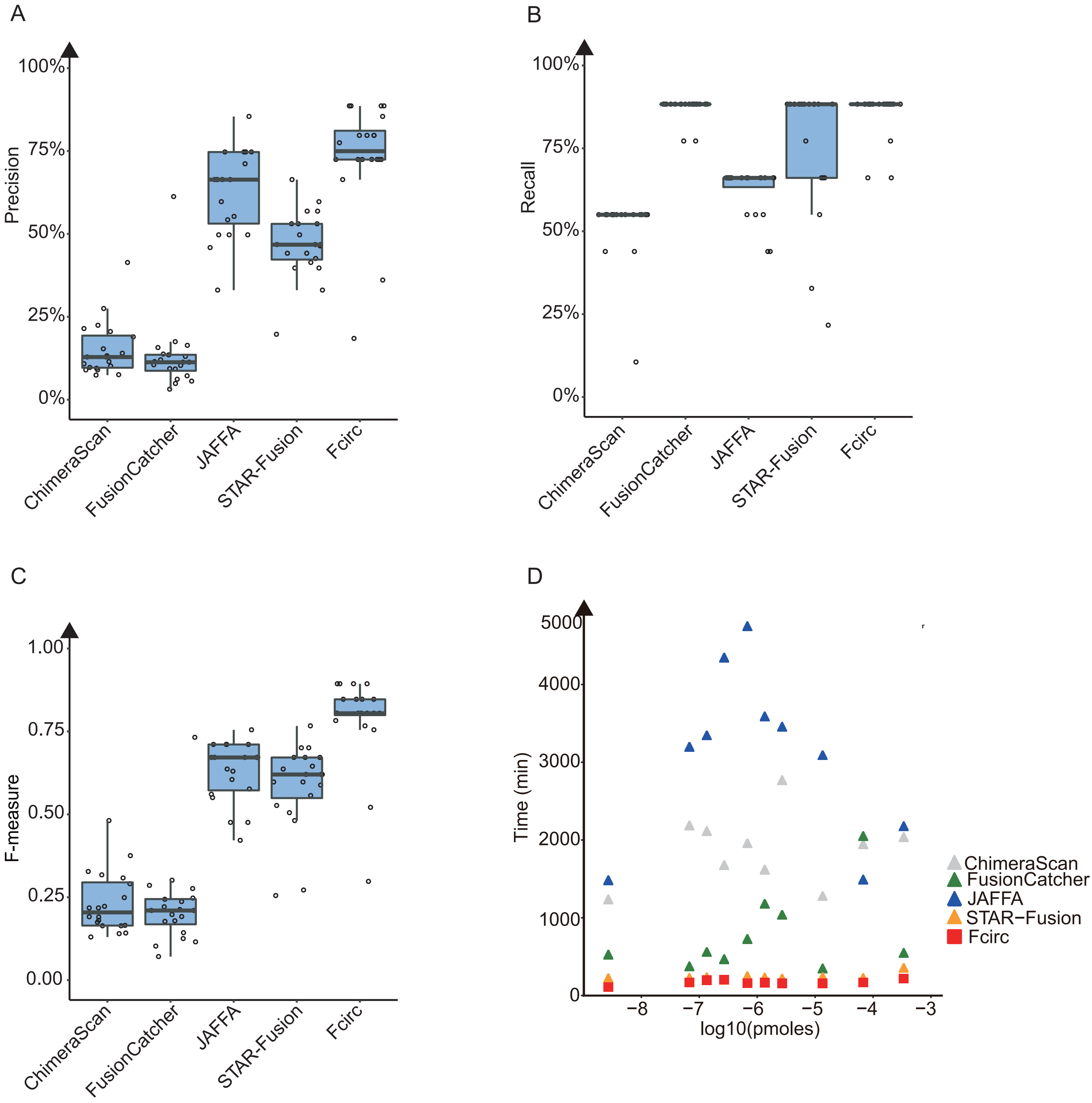

Figure
Fig. 3

Click here to download Figure Fig 3. Supporting read identification in real data.pdf

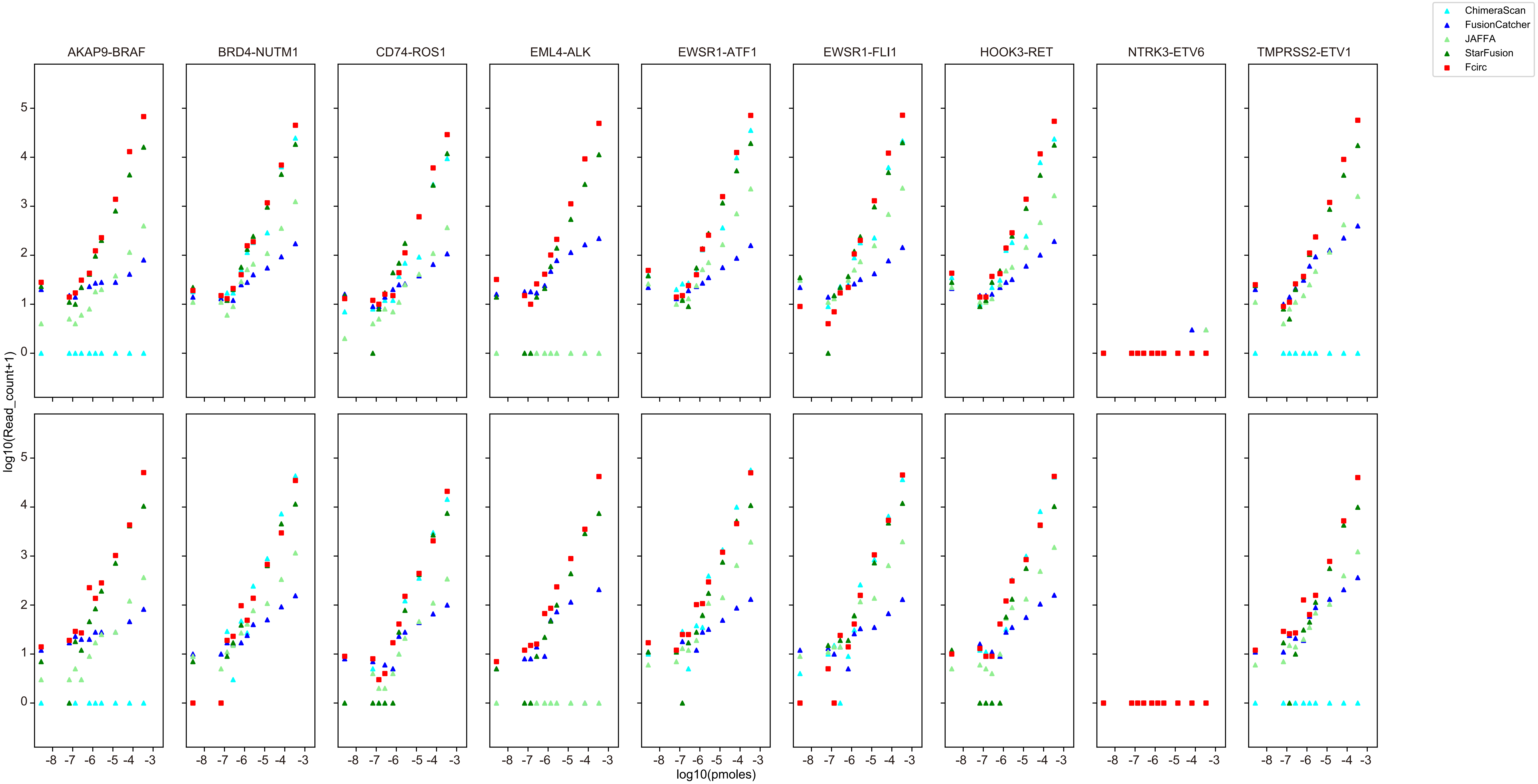

Fig. 4

Click here to download Figure Fig 4. Evaluation in simulated RNA-Seq data.pdf

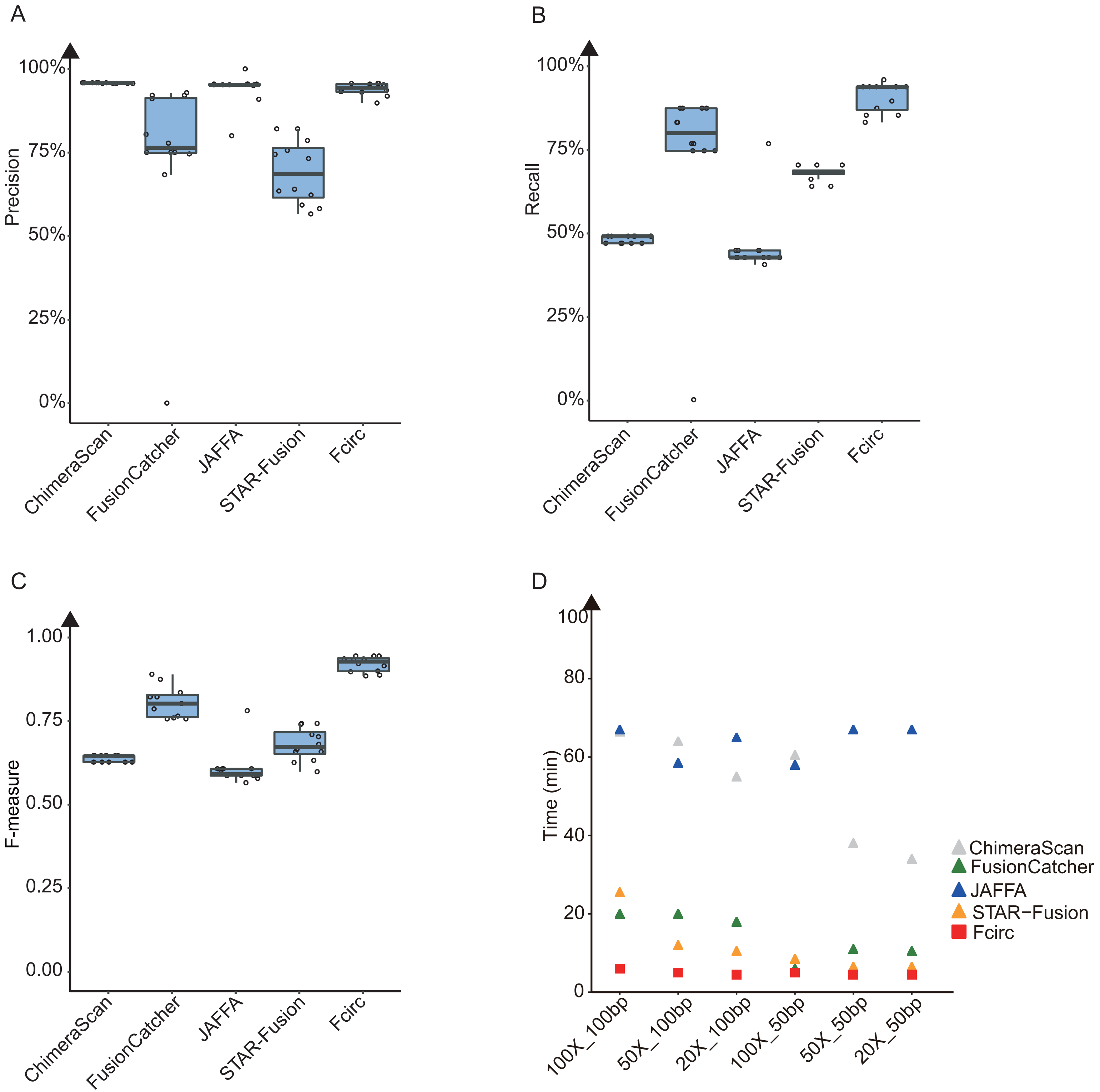

Fig. 5

Click here to download Figure Fig 5. Heatmap of detected f-circRNAs in simulated data.pdf

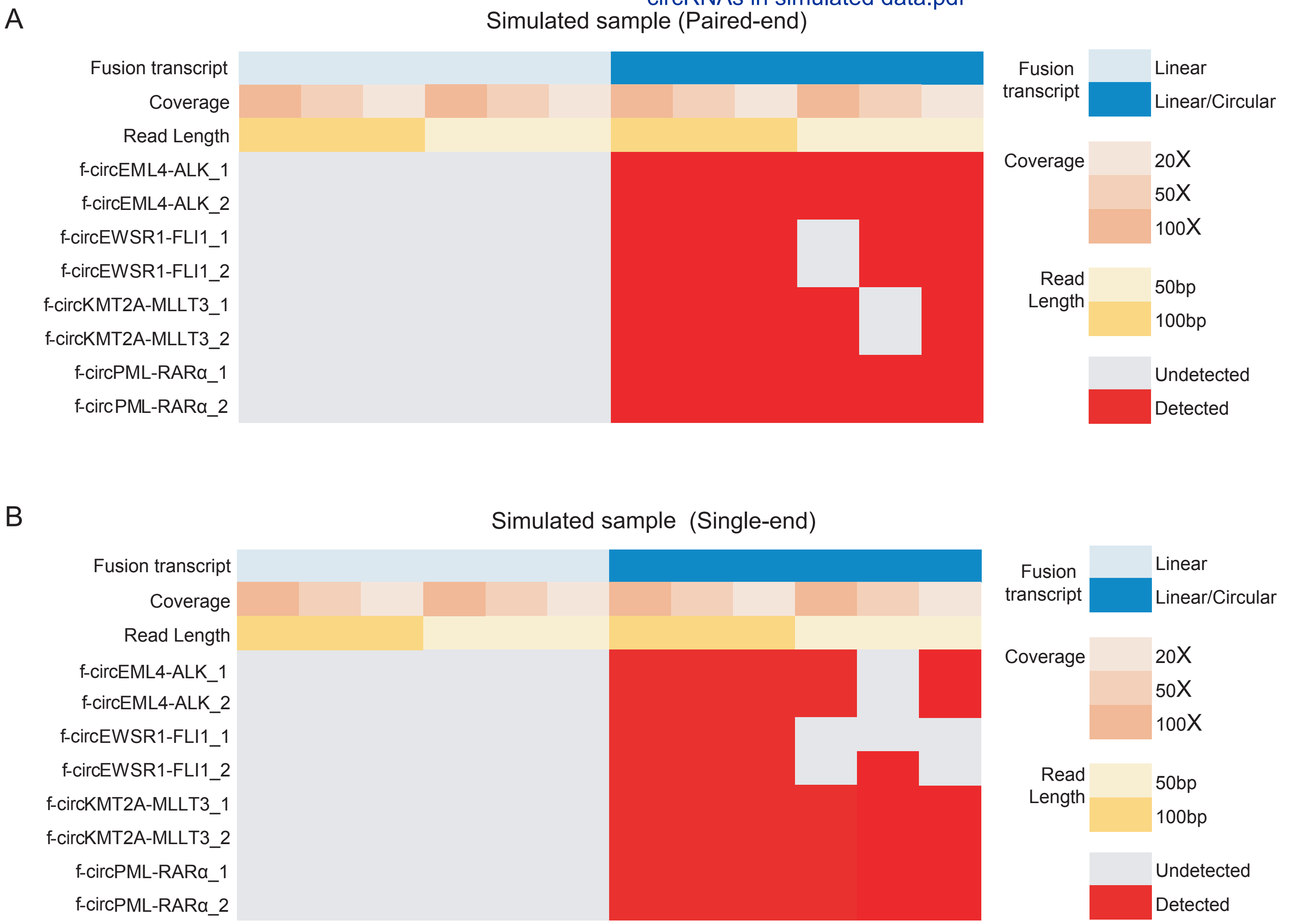

Click here to download Figure Fig 6. Simulated Dataset Four f-circRNAs.pdf

Fig. 6

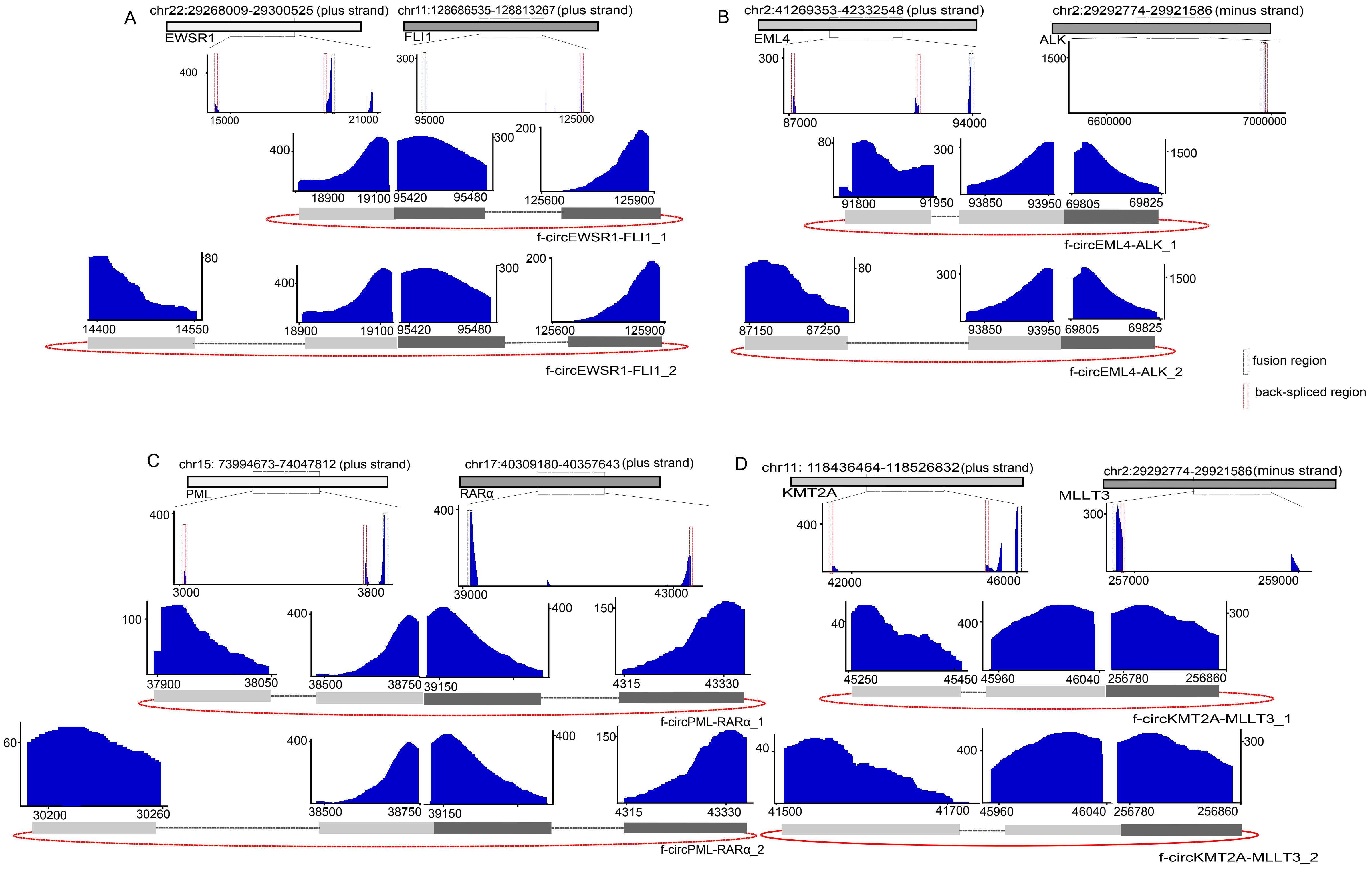

Click here to access/download
Supplementary Material
Supplemental_Fig_S1.pdf

Click here to access/download
Supplementary Material
Supplemental_Fig_S2.pdf

Click here to access/download
Supplementary Material
Supplemental_Tab_S1.xlsx

Click here to access/download
Supplementary Material
Supplemental_Tab_S10.xlsx

Click here to access/download
Supplementary Material
Supplemental_Tab_S11.xlsx

Click here to access/download
Supplementary Material
Supplemental_Tab_S2.xlsx

Click here to access/download
Supplementary Material
Supplemental_Tab_S3.xlsx

Click here to access/download
Supplementary Material
Supplemental_Tab_S4.xlsx

Click here to access/download
Supplementary Material
Supplemental_Tab_S5.xlsx

Click here to access/download
Supplementary Material
Supplemental_Tab_S6.xlsx

Click here to access/download
Supplementary Material
Supplemental_Tab_S7.xlsx

Click here to access/download
Supplementary Material
Supplemental_Tab_S8 _1.xlsx

Click here to access/download
Supplementary Material
Supplemental_Tab_S9.xlsx
