# Supplementary material for: Fcirc: A comprehensive pipeline for the exploration of fusion linear and circular RNAs
Source: Gigascience. 2020 May 29;9(6):giaa054. doi: 10.1093/gigascience/giaa054 (PMC7259471; doi:10.1093/gigascience/giaa054)
Supplement: giaa054_GIGA-D-19-00383_Revision_1 [file giaa054_giga-d-19-00383_revision_1.pdf]

GigaScience

Fcirc: A Comprehensive Pipeline for Exploration of Fusion Linear and Circular RNAs

--Manuscript Draft--

**Manuscript Number:**	GIGA-D-19-00383R1
**Full Title:**	Fcirc: A Comprehensive Pipeline for Exploration of Fusion Linear and Circular RNAs
**Article Type:**	Research
**Funding Information:**	National Natural Science Foundation of China (31771469, 31571363) — Dr. Haiyun Wang<br>National Key Research and Development Program (2017YFC0908500) — Dr. Haiyun Wang
**Abstract:**	Abstract<br>Background<br>Fusion transcripts, as chimeric ribonucleic acids (RNAs) encoded by fusion genes, play an important role in cancer onset and progression, with a molecular therapeutic target for specific cancers. Differing from linear-fusion transcripts, fusion circular RNAs (f-circRNAs) are special circular RNAs produced by fusion genes, which have recently been reported to have an oncogenic role in cancers. RNA sequencing (RNA-Seq) technologies along with existing bioinformatics approaches have enabled researchers to systematically identify fusion transcripts. However, finding f-circRNA in cells introduces a significant challenge for researchers due to the rare occurrence of f-circRNAs. Computational methods to specially identify f-circRNAs have not yet been fully explored.<br>Results<br>Here we have developed a python workflow, Fcirc, a newly comprehensive solution to effectively identify fusion transcripts and f-circRNAs from RNA-Seq data. Fcirc was applied in 3 types of RNA-Seq data sets, including synthetic spike-in real RNA-Seq data, simulated RNA-Seq data and real RNA-Seq data. Fcirc exhibited a significant advantage compared to existing methods in both accuracy and computing performance, empowering us to detect and understand the transcripts and circRNAs of fusion genes in cancers.<br>Conclusion<br>Fcirc is a newly comprehensive solution to effectively identify fusion transcripts and f-circRNAs from RNA-Seq data, and helps to unravel the new f-circRNAs for the further investigation.
**Corresponding Author:**	Haiyun Wang, Ph.D<br>Tongji University<br>Shanghai, Shanghai CHINA
**Corresponding Author Secondary Information:**	
**Corresponding Author's Institution:**	Tongji University
**Corresponding Author's Secondary Institution:**	
**First Author:**	Zhaoqing Cai
**First Author Secondary Information:**	
**Order of Authors:**	Zhaoqing Cai<br>Hongzhang Xue<br>Yue Xu<br>Xiaojie Cheng<br>Yao Dai<br>Jie Zheng

Powered by Editorial Manager® and ProduXion Manager® from Aries Systems Corporation

	Haiyun Wang, Ph.D
**Order of Authors Secondary Information:**	
**Response to Reviewers:**	GIGA-D-19-00383<br>Title: Fcirc: A Comprehensive Pipeline for Exploration of Fusion Linear and Circular RNAs<br><br>EDITORS' COMMENTS TO THE AUTHOR<br>Your manuscript "Fcirc: A Comprehensive Pipeline for Exploration of Fusion Linear and Circular RNAs" (GIGA-D-19-00383) has been assessed by our reviewers. Although it is of interest, we are unable to consider it for publication in its current form. The reviewers have raised a number of points which we believe would improve the manuscript and may allow a revised version to be published in GigaScience.<br><br>GigaScience has a strong focus on reproducibility and reuse, and a reviewer has noticed that some of the software used in the comparison are no longer supported - please acknowledge e some of the limitations (no identification of novel events), use of no longer supported software as well as older versions which might affect the outcomes of some of the comparisons. You must also provide better details, such as parameters to run the different pipelines. Furthermore, please also expand the bench marking to include other tools - to clearly demonstrate the advantage of using Fcirc. More clarification on scoring parameters and how the "synthetic spike-in real RNA-Seq data" was generated, must be provided.<br><br>Their reports, together with any other comments, are below. Please also take a moment to check our website at https://www.editorialmanager.com/giga/ for any additional comments that were saved as attachments.<br><br>In addition, please register any new software application in the bio.tools and SciCrunch.org databases to receive RRID (Research Resource Identification Initiative ID) and biotoolsID identifiers, and include these in your manuscript. This will facilitate tracking, reproducibility and re-use of your tool.<br><br>If you are able to fully address these points, we would encourage you to submit a revised manuscript to GigaScience. Once you have made the necessary corrections, please submit online at: https://www.editorialmanager.com/giga/<br><br>If you have forgotten your username or password please use the "Send Login Details" link to get your login information. For security reasons, your password will be reset.<br><br>Please include a point-by-point within the 'Response to Reviewers' box in the submission system. Please ensure you describe additional experiments that were carried out and include a detailed rebuttal of any criticisms or requested revisions that you disagreed with. Please also ensure that your revised manuscript conforms to the journal style, which can be found in the Instructions for Authors on the journal homepage. If the data and code has been modified in the revision process please be sure to update the public versions of this too.<br><br>The due date for submitting the revised version of your article is 02 Mar 2020.<br><br>I look forward to receiving your revised manuscript soon.<br><br>Best wishes,<br>Nicole Nogoy, Ph.D GigaScience www.gigasciencejournal.com<br><br>Reply: We sincerely appreciate the editor's comments. In our revised manuscript, we have used the latest version of STAR-Fusion, JAFFA, FusionCatcher to re-evaluate the performance of these tools. And two new tools, Arriba and STAR-SEQR, which were reported to have better performance compared with other 20 tools according to the study of Hass et al [1], were also applied for comparison. The detailed parameters used to run these tools are shown in Supple.Tab_S1, S7. In addition, regarding the impacts of scoring parameters on false positives as well as the detailed information of synthetic spike-in real RNA-Seq data, we have made point-to-point reply to reviewers and made the corresponding revisions in the revised manuscript. In addition, we've

registered our software in the bio.tools (biotools:Fcirc) and SciCrunch.org databases (SCR_018089) and included the information in our revised manuscript. Furthermore, we've expanded the benchmarking to real data and clearly demonstrated the advantage of using Fcirc. All of the change in our revised manuscript has been marked in red. We hope you will judge our paper of sufficient interest to merit consideration in GigaScience.

REVIEWERS' COMMENTS TO THE AUTHOR
Reviewer #1: The manuscript by Cai et al describes a new pipeline to annotate fusion transcripts and fusion circular RNAs. The manuscript is overall well written and the tool described of interest especially as several of the tools for gene fusion discovery currently available have relative high false positive rate.

One of the limitation of the application is that it relies on providing known fusions for the detection and does not offer the possibility to explore potential novel events, in doing so the authors limit the use of their pipeline.

Reply: We appreciate the comments and concerns of Reviewer 1. Regarding the limitation of our method, in our revised manuscript we've included two newly developed fusion detection tools, Arriba (v1.1.0) and STAR-SEQR (v0.6.7), which were reported to have better performance compared with other 20 tools according to the study of Hass et al [1], to evaluate the performance of Fcirc (Fig.2, Fig4, Supplemental_Fig_S3).

Some of the software used in the comparison are not maintained (Chimera-Scan - last commit in 2012). Additionally other software (STAR-Fusion, JAFFA) have more recent versions including potential performance improvements (STAR-FUSION), the authors should be careful in drawing conclusions in the comparisons, and acknowledge those potential issues. Additionally the authors should provide the parameters used to run the different pipelines.

Reply: In our revised manuscript, we used the latest version of STAR-Fusion (v1.8.1), JAFFA (v1.09), FusionCatcher (v1.00) to re-evaluate the performance of these tools. In addition, we added two newly developed fusion detection tools, Arriba (v1.1.0) and STAR-SEQR (v0.6.7). According to the study of Haas et al., by comparing 20 fusion detection tools they found that Arriba (v1.1.0) and STAR-SEQR (v0.6.7) gained better performance [1]. So we included these two tools in our revised manuscript. Overall, Fcirc exhibited an advantage compared to these 6 methods in prediction performance and computing time both for synthetic spike-in real RNA-Seq data (Fig.2) and simulated RNA-Seq data (Fig.4).

To make it clear for the readers to understand the parameters of the different fusion detection tool, we made a table to provide the detailed information (Supplemental_Tab_S1) of tools and parameters that were used to run these tools (Supplemental_Tab_S7).

The authors selected 47 fusions from COSMIC, the criteria for selection need to be provided.

Reply:We randomly selected 47 fusions, with high, median and low occurrence frequency in cancers, from the Catalogue Of Somatic Mutations In Cancer (COSMIC) database. We've added the selection criteria in Materials and Methods section of our revised manuscript.

Reviewer #2: In the present manuscript, Zhaoqing Cai and colleagues describe the development of Fcirc, a computational pipeline to detect fusion linear and circular RNAs. The concept of detection fusion transcripts in RNA sequencing data is not new, yet the approach the authors have taken by only looking at known fusion genes drastically increases the performance and decreases the number of false positives. In addition, the tool is also capable of finding fusion circular RNAs. As the authors have indicated, the approach does effectively limit the capability of the tool to find novel fusion events outside of annotated fusion genes.

Major comments

Powered by Editorial Manager® and ProduXion Manager® from Aries Systems Corporation

It is unclear how useful this tool will be given that it can only detect fusion events in known fusion genes and the limited benchmarking on real data is not really convincing. I would suggest expanding this part of the benchmarking and also include the other tools here to clearly show the advantage of Fcirc.

Reply: We understand the concern of Reviewer 2. In our revised manuscript, we've added two newly developed fusion detection tools, Arriba (v1.1.0) and STAR-SEQR (v0.6.7), that were reported to have better performance compared with other 20 tools according to the study of Hass et al.[1], on real data and simulated data evaluation.

Furthermore, we expanded the benchmarking on real data. Differing from synthetic spike-in real data, which was generated for comparative assessment and collaborative development of novel gene fusions detection algorithms, this real data was mainly used to detect f-circRNAs and didn't provide which fusions were truly present in cell lines. To expand the benchmarking on real data, in an alternative way we defined fusions that were detected by at least 4 tools with supporting reads more than 10 as true positives, and then compared the performance of Fcirc with other 6 tools.

Regarding synthetic spike-in real data, we've made the reply in the section below and added the detailed description of this data to Materials and Methods section.

Overall, Fcirc exhibited an advantage compared to other 6 tools in prediction performance both for synthetic spike-in real RNA-Seq data (Fig.2) and simulated RNA-Seq data (Fig.4). As for real data, Fcirc had greater F-measure values, with an excellent precision and a bit decreased recall (Supplemental_Fig_S3).

Page 4, line 76 The authors claim that the "scoring parameters need to be strict to make reads with perfect matches and align few mismatches". But since the unmapped reads are used to find fusion genes, wouldn't making the parameters stricter results in more unmapped reads and thus higher change of false positives?

Reply: We thank the reviewer for this nice suggestion. To make sure how the parameters affect the results, we applied two scoring parameters: our previous stricter one and looser one (by default). When the stricter one was applied, indeed, more unmapped reads and fusion genes were called as shown in the table. That results in the decreased precision and F-measure values as what the reviewer concerned on(Supplemental_Tab _S15). So in our revised manuscript, we applied the default looser parameters to detect fusions.

Page 8 It is unclear how the "synthetic spike-in real RNA-Seq data" was generated. How are the synthetic RNA transcripts created (technology/company)? What RNA sample was used as a basis and what are the concentrations of the sample and the spike-in? What library prep. and sequencer were used? Ideally this data should be deposited in a public resource.

Reply: We sincerely appreciate the comments of Reviewer 2 in this point. In our revised manuscript, we've added the detailed description of this data to Materials and Methods section.

Synthetic spike-in real RNA-Seq data [2] are from a study by Tembe W et al. They generated this publicly available gene-fusion RNA-seq data, with an aim to comparative assessment and collaborative development of novel gene fusions detection algorithms. In this study, they have generated nine synthetic poly-adenylated RNA transcripts that correspond to previously reported oncogenic gene fusions, including EWSR1-ATF1, TMPRSS2-ETV1, EWSR1-FLI1, NTRK3-ETV6, CD74-ROS1, HOOK3-RET, EML4-ALK, AKAP9-BRAF, and BRD4-NUTM1. Equimolar amounts of all nine synthetic gene fusion RNAs were pooled together and this pool was titrated into total RNA from the melanoma cell line COLO-829 at ten different abundances. Libraries were prepared for sequencing using the Illumina TruSeq Stranded mRNA LT Sample Preparation Kit and sequenced on an Illumina HiSeq 2500 (2×101 cycles). The data is available in FASTQ format from the Short Read Archive under accession number SRP043081.

Powered by Editorial Manager® and ProduXion Manager® from Aries Systems Corporation

	Minor comments and typos<br><br>Page 1, line 33 The computational methods … -> Computational methods<br>Reply: We've made the correction.<br><br>Page 4, line 81 change -> changed<br>Reply: We've made the correction.<br><br>Page 10, Figure 2D Why do the authors assume that the runtime would increase with the amount (pmol) of spike-in? Would it not be a function of the number of reads generated in the sequencing? It would actually make more sense to see an effect of the spike-in amount on the precision and recall (figure 2A-C).<br>Reply: Here we don't assume that the runtime increases with the amount of spike in. Actually there is no obvious correlation between the runtime and the amount of spike-in (Fig.2D). I agree with the reviewer that it would make sense to see that prediction performance increases with the amount of spike-in. Because we use fusion-supporting reads to detect fusion genes, and the number of fusion-supporting reads apparently increases with the amount of spike-in (Fig.3).<br><br>Page 14, line 287 the fusion transcript -> fusion transcripts<br>Reply: We've made the correction.<br><br>Figure 2,3, elsewhere The correct abbreviation for picomoles is pmol.<br>Reply: We've made the correction.<br><br>Reference<br>1.Haas BJ, Dobin A, Li B, Stransky N, Pochet N and Regev A. Accuracy assessment of fusion transcript detection via read-mapping and de novo fusion transcript assembly-based methods. Genome Biology. 2019;20 1:213.<br>2.Tembe W, Pond S, Legendre C, Chuang H, Liang WS, Kim N, et al. Open-access synthetic spike-in mRNA-seq data for cancer gene fusions. BMC Genomics. 2014;15 1:824-.
**Additional Information:**	
**Question**	**Response**
Are you submitting this manuscript to a special series or article collection?	No
**Experimental design and statistics**<br><br>Full details of the experimental design and statistical methods used should be given in the Methods section, as detailed in our Minimum Standards Reporting Checklist. Information essential to interpreting the data presented should be made available in the figure legends.<br><br>Have you included all the information requested in your manuscript?	Yes
**Resources**	Yes

A description of all resources used, including antibodies, cell lines, animals and software tools, with enough information to allow them to be uniquely identified, should be included in the Methods section. Authors are strongly encouraged to cite Research Resource Identifiers (RRIDs) for antibodies, model organisms and tools, where possible.<br><br>Have you included the information requested as detailed in our Minimum Standards Reporting Checklist?	
**Availability of data and materials**<br><br>All datasets and code on which the conclusions of the paper rely must be either included in your submission or deposited in publicly available repositories (where available and ethically appropriate), referencing such data using a unique identifier in the references and in the "Availability of Data and Materials" section of your manuscript.<br><br>Have you have met the above requirement as detailed in our Minimum Standards Reporting Checklist?	Yes

Powered by Editorial Manager® and ProduXion Manager® from Aries Systems Corporation

Manuscript
Click here to access/download;Manuscript;main_final_200301.docx

Fcirc: A Comprehensive Pipeline for Exploration of Fusion Linear and Circular RNAs

Zhaoqing Cai[1,#], Hongzhang Xue[2,1#], Yue Xu[1], Xiaojie Cheng[1], Yao Dai[1], Jie Zheng[1], Haiyun Wang[1,*]

[1]*School of Life Sciences and Technology, Tongji University, Shanghai 200092, China*

[2]*School of Life Sciences and Biotechnology, Shanghai Jiao Tong University, Shanghai 200240, China*

E-mail: 1731473@tongji.edu.cn (Cai Z), xuehzh95@foxmail.com(Xue H), 1731490@tongji.edu.cn(Xu Y), siyecaodelvlanzi@163.com(Cheng X), daiyao0808@sina.com(Dai Y), 1931524@tongji.edu.cn (Zheng J), wanghaiyun@tongji.edu.cn(Wang H)

[#] Equal contribution.

[*] Corresponding author.

Abstract

Background

Fusion transcripts, as chimeric ribonucleic acids (RNAs) encoded by fusion genes, play an important role in cancer onset and progression, with a molecular therapeutic target for specific cancers. Differing from linear-fusion transcripts, fusion circular RNAs (f-circRNAs) are special circular RNAs produced by fusion genes, which have recently been reported to have an oncogenic role in cancers. RNA sequencing (RNA-Seq) technologies along with existing bioinformatics approaches have enabled researchers to systematically identify fusion transcripts. However, finding f-circRNA in cells introduces a significant challenge for researchers due to the rare occurrence of f-circRNAs. Computational methods to specially identify f-circRNAs have not yet been fully explored.

Results

Here we have developed a python workflow, Fcirc, a newly comprehensive solution to effectively identify fusion transcripts and f-circRNAs from RNA-Seq data. Fcirc was applied in 3 types of RNA-Seq data sets, including synthetic spike-in real RNA-Seq data, simulated RNA-Seq data and real RNA-Seq data. Fcirc exhibited a significant advantage compared to existing methods in both accuracy and computing performance, empowering us to detect and understand the transcripts and circRNAs of fusion genes in cancers.

Conclusion

Fcirc is a newly comprehensive solution to effectively identify fusion transcripts and

f-circRNAs from RNA-Seq data, and helps to unravel the new f-circRNAs for the further investigation.

Keywords: Fcirc; Fusion linear RNA; Fusion circRNA; Performance benchmarks

Background

In the cancer genome, the presence of a large number of harmful gene mutations, gene rearrangements and chromosomal fragile sites leads to the occurrence of fusion genes [1-6]. Fusion circular RNAs (f-circRNAs) are special circular RNAs (circRNAs) produced by fusion genes that help cellular transformation, promote cell viability, confer therapeutic resistance to cells, and have tumour-promoting properties [7]. In addition, f-circRNAs also have the potential to be novel liquid biopsy biomarkers [8]. Advances in high-throughput RNA-Seq data enable the detection of many fusions [9-14], as well as circRNAs [15-17]. However, novel fusion detection tools yield a high false discovery rate [18], and current bioinformatics methods cannot be used to fully identify f-circRNAs [19].

Here we present Fcirc (Fig 1, see methods), a comprehensive workflow that explores linear RNAs and circRNAs of fusions. Differing from recently published fusion detection methods such as Arriba [20], ChimeraScan [13], JAFFA [14], FusionCatcher [11], STAR-Fusion [12] and STAR-SEQR [21], Fcirc preferentially detects RNAs coming from known fusion events, resulting in competing computing speed and reduced false positives. Moreover, Fcirc is able to detect the circular

transcripts of fusion genes. Therefore, Fcirc is a no-cost solution to effectively utilize public RNA-Seq data to quickly identify fusion transcripts and f-circRNAs.

Materials and methods

The pipeline of Fcirc

The pipeline of Fcirc executes analysis by following five major steps (Fig 1). As input, Fcirc requires single-end or paired-end RNA-Seq data in FASTQ format. Either raw data or clean data (e.g. cutting the adapter and trimming poor-quality) is acceptable.

1) Dropping the aligned reads

Reads were aligned to a reference transcriptome with HISAT2 [22] using the default parameters. After the first alignment was completed, the aligned reads were dropped by samtools [23] and the unaligned reads were kept for further analysis. For single-end RNA-Seq data, reads with a FLAG value of 4 in the Sequence Alignment / Map format (SAM) file were selected (-f 4) and converted into files in FASTQ format. For paired-end RNA-Seq data, reads without a FLAG value of 2 in the SAM file were selected (-F 2), meaning that either segment of a read was unaligned. Then, they were converted into a file in FASTQ format.

2) Building a bipartite graph of gene pairs of known fusions

Gene pairs of known fusions were manually curated from multiple databases, including COSMIC [24], ChimerDB [25], TicDB [26], FARE-CAFE [27] and FusionCancer [28]. A bipartite graph (or bigraph) is a graph whose vertices can be divided into two disjoint and independent sets **U** and **V** such that every edge connects

a vertex in **U** to one in **V**. Genes were vertices and fusion events were edges. The genes involved in the fusion event did not form a ring of odd vertices, so a bipartite graph of known fusion gene-pairs could be built. To reduce the computational and time complexity of searching reads that span multiple genes, genes involved in the fusion event were divided into two independent sets according to the bipartite graph. For example, *EML4-ALK* and *NPM1-ALK* have been reported to be fusion genes so that *EML4* and *NPM1* were in the same set while *ALK* was in the rest of the data set. The gene sequences of known fusions were downloaded from the database ensemble [29].

3) Selecting fusion-related reads

The unaligned reads in the first step were re-aligned to two sets of fusion gene sequences with low penalty, independently. We decreased the maximum and minimum penalty for soft-clipping (--sp 1, 1) and minimum alignment score (--score-min L, 0, -0.8). Other scoring parameters were set as the default. After this re-alignment, reads with part of sequences aligned to fusion genes were selected. For single-end RNA-Seq data, reads without a FLAG value of 4 in the SAM file were selected (-F 4). For paired-end RNA-Seq data, reads with a FLAG value of 4, not 8, or 8, not 4 or 12 in the SAM file were selected (-f 4 -F 8 or -F 4 -f 8 or -f 12), which means that at least one segment of a read were aligned. The reads with paired chiastic clipping (PCC) signal were defined as fusion-related reads. For instance, if a segment of a read was aligned to *EML4* with the same FLAG and CIGAR 40S60M values while it was aligned to *ALK* with FLAG 4 and CIGAR 40M60S, it suggested that one

part of a segment was from *EML4* and the rest was from *ALK* in the same strand.

4) Reconstructing and verifying the fusion genes

Next, the fusion-related reads determined the fusion breakpoint. On the assumption that fusion-related reads were more likely to cover the fusion breakpoint, we preferred the junction covered by the most fusion-related reads as the fusion breakpoint, which is simply inferred by the majority of junction-supported reads. Then, the sequences of the fusion gene were reconstructed by this predicted fusion breakpoint.

After the reconstruction of the fusion gene, the alignment of reads was recalibrated by re-aligning reads to the reconstructed fusion gene with a low penalty. We assumed that the fusion-related reads uniformly covered the fusion breakpoint. To evaluate if the fusion-related reads uniformly covered the fusion breakpoint, the fusion-related reads were split into two fragments: the left fragments and right fragments around the breakpoint. The Wilcoxon Sign Rank Test was used to evaluate the distribution of reads, by comparing the length of the left fragments with one of the right fragments. Only a breakpoint with the same distribution of read length distribution at its flanking region was determined as the true breakpoint for the fusion genes.

5) Transforming back-spliced reads

The circular RNAs of fusion genes were detected by searching for back-spliced reads. To improve alignment between back-spliced reads with the reconstructed fusion gene, we changed the order of aligned and unaligned segments of some back-spliced reads to transform back-spliced reads to forward-spliced reads. The transformed reads were

re-aligned to the reconstructed fusion gene to evaluate whether they were truly back-spliced. Those reads covering a back-spliced junction indicated that they were from f-circRNA.

As output, Fcirc provides tables of fusion transcripts and f-circRNAs. To help visualize the reads with their distribution on fusion, Fcirc also provides the fusion-related reads in SAM format.

Performance benchmarks and evaluation criteria

Fcirc and six currently published fusion detection methods (Supplemental_Tab_S1), including Arriba v1.1.0 [20], ChimeraScan v0.4.5 [13], FusionCatcher v1.00 [11], JAFFA v1.09 [14], STAR-Fusion v1.8.1 [12] and STAR-SEQR v0.6.7 [21], were applied to the synthetic spike-in real RNA-Seq data, simulated RNA-Seq data and real RNA-Seq data. To accurately evaluate these tools, we defined that 1) the number of fusion-supporting reads must be at least 3, and 2) read-through transcripts (two genes located on the same chromosome less than 100,000 bp apart) are removed. The computational efficiency of the tools was evaluated by using several criteria. The primary benchmark was precision, recall and F-measure.

$$precision = TP/(TP + FP)$$

$$recall = TP/(TP + FN)$$

$$F - measure = precision * recall * 2/(precison + recall)$$

Where TP, FP and FN represent the true positives, false positives and false negatives, respectively. The F-measure simultaneously considers the effect of precision and recall.

We also evaluated the number of supporting reads identified by the different tools, that reflected the ability to robustly detect the fusion. The last benchmark was the computing time of the different tools.

Datasets

Synthetic spike-in real RNA-Seq data

We applied the synthetic spike-in RNA-Seq data from the study of Tembe W et al. [30] to evaluate the performance of tools. This study consisted of 9 synthetic poly-adenylated RNA transcripts that corresponded to reported cancer fusion genes, including *EWSR1-ATF1*, *TMPRSS2-ETV1*, *EWSR1-FLI1*, *NTRK3-ETV6*, *CD74-ROS1*, *HOOK3-RET*, *EML4-ALK*, *AKAP9-BRAF*, and *BRD4-NUTM1*. Tembe W et al. generated the publicly available gene-fusion RNA-Seq data, with an aim to comparative assessment and collaborative development of novel gene fusions detection algorithms. In this study, equimolar amounts of all 9 synthetic gene fusion RNAs were pooled together and this pool was titrated into total RNA from the melanoma cell line COLO-829 at 10 different abundances, with 2 replicates for each sample. Libraries were prepared for sequencing using the Illumina TruSeq Stranded mRNA LT Sample Preparation Kit and sequenced on an Illumina HiSeq 2500 (2 × 101 cycles). The data is available in FASTQ format from the Short Read Archive under accession number SRP043081.

Simulated RNA-Seq data

The simulator art_illumina function in ART [31] was applied to generate simulated RNA-Seq data. Using the RNA-Seq reads in normal pulmonary microvascular

endothelial cells from the National Center for Biotechnology Information (NCBI) Sequence Read Archive (SRA) database SRR349695 [32] as the background, we plugged the simulated fusion reads into the background reads. Two types of fusion reads were designed, with the first one from the linear transcripts and the second one from the linear and circular pooled transcripts. A total of 47 fusions (Supplemental_Tab_S2), with high, median and low occurrence frequency in cancers, were randomly selected from the Catalogue Of Somatic Mutations In Cancer (COSMIC) database [24]. And the linear fusion reads were artificially generated based on the breakpoint information by joining the upstream transcript fragment and downstream transcript fragment. Eight fusion circRNAs (Supplemental_Fig_S1, Supplemental_Tab_S3) were generated according to previous reports of f-circRNAs [7, 33].

To simulate more linear fusion transcripts than circular transcripts at a gene locus, the amount of linear fusion reads plugged into the background reads was 2.5 times as many as that of circular fusion reads. Different sequencing coverage including 20X, 50X, 100X, each with two read lengths of 50 bp and 100 bp were designed in the simulated data.

Real data

Real data for f-circRNA identification were obtained from the BioProject with accession numbers PRJNA350335 and PRJNA315254. The data set PRJNA350335 includes 9 cell lines of H3122 harbouring the *EML4-ALK* fusion gene [34]. The data set PRJNA315254 includes 9 acute leukaemia samples with 3 NB4 cell lines, 1 THP1

cell lines and 5 primary patients harbouring the *PML-RARα* fusion gene [7].

Results

Evaluation of gene fusions in synthetic spike-in real and simulated RNA-Seq data

In synthetic RNA-Seq data, 9 synthetic cancer-associated fusion genes, including *EWSR1-ATF1*, *TMPRSS2-ETV1*, *EWSR1-FLI1*, *NTRK3-ETV6*, *CD74-ROS1*, *HOOK3-RET*, *EML4-ALK*, *AKAP9-BRAF*, and *BRD4-NUTM1*, were spiked-in (see methods). Compared with the other tools, Fcirc (Fig 2A, Supplemental_Tab_S4-1) achieved the highest precision (87.50%), followed by STAR-SEQR (81.90%) and Arriba (78.00%). ChimeraScan (6.10%) and FusionCatcher (13.4%) obtained low precision values, suggesting a high risk of false positives for their predictions. In addition, Fcirc (86.68%), ChimeraScan (80.57%), STAR-Fusion (78.90%) and Arriba (76.68%) achieved higher recall than FusionCatcher (76.14%), JAFFA (73.92%) and STAR-SEQR (58.35%) (Fig 2B, Supplemental_Tab_S4-2). Fcirc had greater F-measure values (0.86), indicating its better performance for balancing precision and recall (Fig 2C, Supplemental_Tab_S5). In addition, STAR-SEQR and Fcirc required less computing time among these tools, followed by Arriba (Fig 2D, Supplemental_Tab_S6). The computational environment was based on Ubuntu Linux with Intel Xeon E5-2620 v4 CPU@ 2.10GHz, and four CPU cores were used for each tool. The running parameters for each tool were included in Supplemental_Tab_S7.

There were 10 different concentrations for 9 types of synthetic gene fusion RNA,

with 2 replicates for each RNA-Seq dataset. We calculated the number of fusion-supporting reads, the junction reads for identifying gene fusions, for the different tools. The results (Fig 3) showed that Fcirc (red squares) identified the highest number of supporting reads under different concentrations, at which the synthetic gene fusion RNA constructs were spiked into total RNA. Moreover, with increasing concentrations, the number of identified supporting reads of gene fusions grew rapidly.

We also evaluated the performance of 7 tools in the simulated paired-end data (see methods). In these data, Fcirc (Fig 4A-B, Supplemental_Tab_S8) achieved higher precision (98.02%) than the other tools, and high recall (85.64%), which is a little lower than Arriba (86.36%). The highest F-measures (Fig 4C, Supplemental_Tab_S9) were generated by Fcirc (0.91) in all of simulated data, followed by Arriba (0.86). Arriba and Fcirc exhibited advantages of computing time in simulated paired-end data (Fig 4D, Supplemental_Tab_S10). Among these tools, ChimeraScan and JAFFA required the longer computing time for nearly all the data. As for computing time in single-end data, the computing time of Fcirc was in less than 5 minutes (Supplemental_Fig_S2).

Evaluation of f-circRNAs in simulated RNA-Seq data

To evaluate the ability of the tools to identify f-circRNA, we designed reads of 8 fusion circRNAs according to previous reports and plugged them into RNA-Seq data from normal pulmonary microvascular endothelial cells (see methods). We designed two types of RNA-Seq data, the first one was a control, containing only the linear

fusion transcripts, and the second one contained the linear/circular fusion transcripts. Different sequencing coverage and read lengths were considered in the simulated data. In simulated paired-end samples (Fig 5A), Fcirc successfully detected 8 types of f-circRNAs from RNA-Seq data containing linear/circular fusion transcripts. As expected, no f-circRNAs were detected from the RNA-Seq data only containing linear fusion transcripts. Fcirc also worked well in simulated single-end samples (Fig 5B). Moreover, all f-circRNAs were identified when the read length was 100 bp, both in paired-end samples and single-end samples. More f-circRNAs were identified in the paired-end samples than in single-end samples when the read length was 50 bp under the same coverage. Eight types of f-circRNAs from 4 fusion genes (*EML4-ALK*, *EWSR1-FLI1*, *KMT2A-MLLT3*, *PML-RARα*) identified in the paired-end sample with 100X coverage and read length of 100 bp were visualized (Fig 6).

Identification of f-circRNAs in real data

Next, we identified f-circRNAs based on real data obtained from the BioProject with accession numbers PRJNA350335 and PRJNA315254. PRJNA350335 includes 9 cell lines of H3122 harbouring the *EML4-ALK* fusion gene, and PRJNA315254 includes 9 acute leukaemia samples with 3 NB4 cell lines, 1 THP1 cell lines and 5 primary patients harbouring the *PML-RARα* fusion gene.

In the data set PRJNA350335, we applied Fcirc to detect the linear and circular fusion transcripts. Fcirc successfully identified *EML4-ALK* fusion at a specific fusion breakpoint in 9 H3122 cell lines with the amount of supporting reads (Supplemental_Tab_S11), which is consistent with the previous report [34]. In

addition to the capability of identifying fusion linear transcripts such as many fusion detection tools, Fcirc is newly designed to detect f-circRNA as well by detecting the back-spliced reads in a fusion gene. As we expected, we successfully identified the previously reported f-circRNA *EML4-ALK* [8, 35] (Supplemental_Tab_S12).

In the meantime, we also evaluated the performance of 7 tools in the data set PRJNA350335. PRJNA350335 does not provide which fusions are truly present in cell lines. In an alternative way, we defined fusions that were detected by at least 4 tools with supporting reads more than 10 as true positives, and then compared the performance of 7 tools. Fcirc (100%) achieved the highest precision than other tools (Supplemental_Fig_S3A, Supplemental_Tab_S13-1). ChimeraScan (100%), STAR-Fusion (100%) and STAR-SEQR (100%) achieved higher recall than other tools (Supplemental_Fig_S3B, Supplemental_Tab_S13-2). STAR-SEQR (0.889) and Fcirc (0.815) had higher F-measures than other tools (Supplemental_Fig_S3C, Supplemental_Tab_S13-3). The results showed that Fcirc had great precision and F-measure values, with a bit decreased recall due to unknown fusions.

In the data set PRJNA315254, we detected the *KMT2A-MLLT3* (*MLL-AF9*) fusion and *PML-RARα* fusion with an amount of supporting reads (Supplemental_Tab_S14), which was consistent with a previous study [7]. Interestingly, the different types of f-circRNA isoforms for the *PML-RARα* fusion were detected in this dataset (Supplemental_Tab_S12). When the cutoff of f-circRNA-supporting reads counts were 1 or 2, 18 or 8 f-circRNA isoforms from the PML-RARα fusion were respectively detected in the RNA-Seq data of the NB4 cell line SRR3239817.

PML-RARα f-circRNAs have been reported in the study of Guarnerio et al [7]. All fusion genes identified in the data sets PRJNA350335 and PRJNA315254 are shown in Supplemental_Tab_S14.

Discussion

Fusion linear and circular transcripts can be involved in a variety of cancerous transformations, indicating their potential diagnostic and therapeutic implications. Currently, a large amount of RNA-Seq data is widely available, making it feasible to efficiently take advantage of these data for f-circRNA predictions. It is a significant challenge to find f-circRNA in cells due to its rare occurrence. Here, we have developed a python workflow, Fcirc, a newly comprehensive solution to effectively identify fusion transcripts and f-circRNAs from public RNA-Seq data. In this study, we compared the performance of Fcirc with six fusion detection tools in synthetic spike-in real, simulated data sets and real data sets. Fcirc achieved advantages in precision, recall, fusion-supporting reads number and computing time. Our results provide an insightful comparison of different fusion detection tools and indicate that Fcirc is a reliable tool for fusion detection. Moreover, Fcirc successfully detected all f-circRNAs in simulated data and the f-circRNAs (*EML4-ALK*, *PML-RARα*) reported by previous studies[7, 8, 35] in real data. In the human promyelocytic leukaemia cell line NB4, approximately 10 f-circRNA transcripts were identified, which is worth further investigation.

Our pipeline exhibits significant advantages compared to existing methods.

RNA-Seq data suffers from heavy background noise leading to unexpected false positive detection. For example, in the synthetic data set, only 6.10% of fusion transcripts predicted by ChimeraScan and 13.4% of fusion transcripts predicted by FusionCatcher are true positives. Regarding this issue, Fcirc uses known fusions as a reference to build a bipartite graph of gene pairs, dramatically decreasing false positives. Moreover, this also greatly reduces the computing time, and Fcirc computed in the simulated RNA-Seq data in minutes, significantly expediting performance speed and outperforming the current tools.

Though at the cost of losing the ability to identify new fusion genes not reported by fusion gene databases, our method updates known fusion genes collected from the multiple databases in a timely manner, and the user can add their own fusion genes by using optional input. In conclusion, Fcirc will help us better detect and understand the transcripts and circRNAs of fusion genes in cancers.

Availability of Supporting Source Code and Requirements

Project name: Fcirc: A Comprehensive Pipeline for Exploration of Fusion, Linear and Circular RNAs

Project home page: https://github.com/WangHYLab/fcirc

Operating system(s): Ubuntu 16.04/18.04, MacOS

Programming language: Python

Other requirements: hisat2, samtools, numpy, scipy, pysam

License: MIT

Bio.tools id: biotools:Fcirc

Project RRID: SCR_018089

Availability of Supporting Data and Materials

Synthetic spike-in real RNA-Seq data was obtained from the Short Read Archive under accession number SRP043081. Real data was obtained from the BioProject with accession numbers PRJNA350335 and PRJNA315254. Simulated RNA-Seq data was obtained by the method in Method section, and reference information of fusion transcripts and f-circRNAs were showed in Additional files section.

Abbreviations

RNA: ribonucleic acid; RNA-Seq: ribonucleic acid sequencing; f-circRNA: fusion-circular ribonucleic acid; circRNA: circular ribonucleic acid; SAM: Sequence Alignment/Map format; PCC: paired chiastic clipping; TP: true positive; FP: false positive; FN: false negative; SRA: Sequence Read Archive; NCBI: National Center for Biotechnology Information; COSMIC: Catalogue Of Somatic Mutations In Cancer

Competing interests

The authors declare no competing interests.

Authors' contributions

HW conceived the hypothesis. ZC, HX, XC, YD, and JZ designed and performed the pipeline and analysis. HW, HX and ZC wrote the manuscript.

Acknowledgements

This work was supported by grants from the National Natural Science Foundation of China (31771469 and 31571363 to HW), and a grant from the National Key Research and Development Program (2017YFC0908500 to HW).

References

1. Lebeau MM and Rowley JD. Cancer biology: Heritable fragile sites in cancer. Nature. 1984;308 5960:607-8.
2. Stratton MR, Campbell PJ and Futreal PA. The cancer genome. Nature. 2009;458 7239:719-24.
3. Huebner K. Molecular biology: DNA fragility put into context. Nature. 2011;470 7332:46-7.
4. Coquelle A, Toledo F, Stern S, Bieth A and Debatisse M. A New Role for Hypoxia in Tumor Progression: Induction of Fragile Site Triggering Genomic Rearrangements and Formation of Complex DMs and HSRs. Molecular Cell. 1998;2 2:259-65.
5. Novo FJ and Vizmanos JL. Chromosome translocations in cancer: computational evidence for the random generation of double-strand breaks. Trends in Genetics. 2006;22 4:193-6.
6. Imielinski M and Ladanyi M. Fusion oncogenes—genetic musical chairs. Science. 2018;361 6405:848-9.
7. Guarnerio J, Bezzi M, Jeong JC, Paffenholz SV, Berry K, Naldini MM, et al. Oncogenic Role of Fusion-circRNAs Derived from Cancer-Associated Chromosomal Translocations. Cell. 2016;165 2:289-302. doi:10.1016/j.cell.2016.03.020.
8. Tan S, Gou Q, Pu W, Guo C, Yang Y, Wu K, et al. Circular RNA F-circEA produced from EML4-ALK fusion gene as a novel liquid biopsy biomarker for non-small cell lung cancer. Cell research. 2018;28 6:693-5. doi:10.1038/s41422-018-0033-7.
9. Wang K, Singh D, Zeng Z, Coleman SJ, Huang Y, Savich GL, et al. MapSplice: accurate mapping

of RNA-seq reads for splice junction discovery. Nucleic Acids Res. 2010;38 18:e178. doi:10.1093/nar/gkq622.

10. Kim D and Salzberg SL. TopHat-Fusion: an algorithm for discovery of novel fusion transcripts. Genome Biol. 2011;12 8:R72. doi:10.1186/gb-2011-12-8-r72.

11. Nicorici D, Şatalan M, Edgren H, Kangaspeska S, Murumägi A, Kallioniemi O, et al. FusionCatcher – a tool for finding somatic fusion genes in paired-end RNA-sequencing data. bioRxiv. 2014:011650. doi:10.1101/011650.

12. Haas BJ, Dobin A, Stransky N, Li B, Yang X, Tickle T, et al. STAR-Fusion: Fast and Accurate Fusion Transcript Detection from RNA-Seq. bioRxiv. 2017:120295. doi:10.1101/120295.

13. Iyer MK, Chinnaiyan AM and Maher CA. ChimeraScan: a tool for identifying chimeric transcription in sequencing data. Bioinformatics. 2011;27 20:2903-4. doi:10.1093/bioinformatics/btr467.

14. Davidson NM, Majewski IJ and Oshlack A. JAFFA: High sensitivity transcriptome-focused fusion gene detection. Genome Med. 2015;7 1:43. doi:10.1186/s13073-015-0167-x.

15. Gao Y, Wang J and Zhao F. CIRI: an efficient and unbiased algorithm for de novo circular RNA identification. Genome Biol. 2015;16:4. doi:10.1186/s13059-014-0571-3.

16. Szabo L, Morey R, Palpant NJ, Wang PL, Afari N, Jiang C, et al. Statistically based splicing detection reveals neural enrichment and tissue-specific induction of circular RNA during human fetal development. Genome Biol. 2015;16:126. doi:10.1186/s13059-015-0690-5.

17. Song X, Zhang N, Han P, Moon BS, Lai RK, Wang K, et al. Circular RNA profile in gliomas revealed by identification tool UROBORUS. Nucleic Acids Res. 2016;44 9:e87. doi:10.1093/nar/gkw075.

18. Kumar S, Vo AD, Qin F and Li H. Comparative assessment of methods for the fusion transcripts detection from RNA-Seq data. Sci Rep. 2016;6:21597. doi:10.1038/srep21597.

19. Zeng X, Lin W, Guo M and Zou Q. A comprehensive overview and evaluation of circular RNA detection tools. PLOS Computational Biology. 2017;13 6:e1005420. doi:10.1371/journal.pcbi.1005420.

20. Uhrig S, Fröhlich M, Hutter B and Brors B. PO-400 Arriba–fast and accurate gene fusion detection from RNA-seq data. BMJ Publishing Group Limited, 2018.

21. Jasper J, Powers JG and Weigman VJ. STAR-SEQR: Accurate fusion detection and support for fusion neoantigen applications. AACR, 2018.

22. Kim D, Langmead B and Salzberg SL. HISAT: a fast spliced aligner with low memory requirements. Nature Methods. 2015;12:357. doi:10.1038/nmeth.3317.

23. Li H, Handsaker B, Wysoker A, Fennell T, Ruan J, Homer N, et al. The Sequence Alignment/Map format and SAMtools. Bioinformatics. 2009;25 16:2078-9. doi:10.1093/bioinformatics/btp352.

24. Forbes SA, Beare D, Boutselakis H, Bamford S, Bindal N, Tate J, et al. COSMIC: somatic cancer genetics at high-resolution. Nucleic Acids Res. 2017;45 D1:D777-d83. doi:10.1093/nar/gkw1121.

25. Lee M, Lee K, Yu N, Jang I, Choi I, Kim P, et al. ChimerDB 3.0: an enhanced database for fusion genes from cancer transcriptome and literature data mining. Nucleic Acids Research. 2017;45 Database issue:D784-D9.

26. Novo FJ, Mendíbil IOD and Vizmanos JL. TICdb: a collection of gene-mapped translocation

breakpoints in cancer. Bmc Genomics. 2007;8 1:33.
27. Korla PK, Cheng J, Huang CH, Tsai JJ, Liu YH, Kurubanjerdjit N, et al. FARE-CAFE: a database of functional and regulatory elements of cancer-associated fusion events. Database (Oxford). 2015;2015 doi:10.1093/database/bav086.
28. Wang Y, Wu N, Liu J, Wu Z and Dong D. FusionCancer: a database of cancer fusion genes derived from RNA-seq data. Diagnostic Pathology. 2015;10 1:131.
29. Zerbino DR, Achuthan P, Akanni W, Amode MR, Barrell D, Bhai J, et al. Ensembl 2018. Nucleic Acids Research. 2018;46 Database issue:D754.
30. Tembe WD, Pond SJ, Legendre C, Chuang HY, Liang WS, Kim NE, et al. Open-access synthetic spike-in mRNA-seq data for cancer gene fusions. BMC Genomics. 2014;15:824. doi:10.1186/1471-2164-15-824.
31. Huang W, Li L, Myers JR and Marth GT. ART: a next-generation sequencing read simulator. Bioinformatics. 2012;28 4:593-4. doi:10.1093/bioinformatics/btr708.
32. Zhang LQ, Cheranova D, Gibson M, Ding S, Heruth DP, Fang D, et al. RNA-seq reveals novel transcriptome of genes and their isoforms in human pulmonary microvascular endothelial cells treated with thrombin. PloS one. 2012;7 2:e31229. doi:10.1371/journal.pone.0031229.
33. Tan S, Gou Q, Pu W, Guo C, Yang Y, Wu K, et al. Circular RNA F-circEA produced from EML4-ALK fusion gene as a novel liquid biopsy biomarker for non-small cell lung cancer. Cell Research. 2018;28 6:693-5. doi:10.1038/s41422-018-0033-7.
34. Rusan M, Li K, Li Y, Christensen CL, Abraham BJ, Kwiatkowski N, et al. Suppression of Adaptive Responses to Targeted Cancer Therapy by Transcriptional Repression. Cancer Discov. 2018;8 1:59-73. doi:10.1158/2159-8290.cd-17-0461.
35. Tan S, Sun D, Pu W, Gou Q, Guo C, Gong Y, et al. Circular RNA F-circEA-2a derived from EML4-ALK fusion gene promotes cell migration and invasion in non-small cell lung cancer. Mol Cancer. 2018;17 1:138. doi:10.1186/s12943-018-0887-9.

Figure legends

Figure 1. Fcirc pipeline for exploring linear and circular RNAs of known fusions

Five main steps of Fcirc, including dropping aligned reads, building a bipartite graph of gene pairs of known fusions, selecting fusion-related reads, reconstructing and verifying the fusion genes and transforming back-spliced reads.

Figure 2. Performance comparison of the different gene fusion detection tools in synthetic spike-in real RNA-Seq data

Comparison of precision (**A**), recall (**B**), F-measure (**C**) and computing time (**D**) across seven fusion detection tools, including Arriba, ChimeraScan, FusionCatcher, JAFFA, STAR-Fusion, STAR-SEQR and Fcirc.

Figure 3. Fusion-supporting reads identified by the different gene fusion detection tools in synthetic spike-in real RNA-Seq data

The amount of fusion-supporting reads in 9 fusions (*AKAP9-BRAF*, *BRD4-NUTM1*, *CD74-ROS1*, *EML4-ALK*, *EWSR1-ATF1*, *EWSR1-FLI1*, *HOOK3-RET*, *NTRK3-ETV6*, and *TMPRSS2-ETV1*) identified by Arriba, ChimeraScan, FusionCatcher, JAFFA, STAR-Fusion, STAR-SEQR and Fcirc in synthetic spike-in real RNA-Seq data. Each fusion has two replicates.

Figure 4. Performance comparison of the different gene fusion detection tools in simulated RNA-Seq data

Comparison of precision (**A**), recall (**B**), F-measure(**C**), and computing time (**D**) across seven fusion detection tools, including Arriba, ChimeraScan, FusionCatcher, JAFFA, STAR-Fusion, STAR-SEQR and Fcirc.

Figure 5. The identification of f-circRNAs in paired-end (A) and single-end (B) simulated RNA-Seq data

Eight types of f-circRNAs from 4 fusion genes (*EML4-ALK*, *EWSR1-FLI1*, *KMT2A-MLLT3*, and *PML-RARα*) were included in simulated RNA-Seq data. Two types of RNA-Seq data, with the first one as a control only containing the linear fusion transcripts and the second one containing the linear/circular fusion transcripts,

were designed. Different sequencing coverage including 20X, 50X, 100X, each with two read length of 50 bp and 100 bp, were designed.

Figure 6. Visualization of f-circRNAs

f-circ*EWSR1-FLI1*(**A**), f-circ*EML4-ALK*(**B**), f-circ*PML-RARα*(**C**), f-circ*KMT2A-MLLT3* (**D**), identified from simulated RNA-Seq data. These f-circRNAs were identified in the paired-end samples with 100X coverage and read length of 100 bp. For each fusion gene, there are 2 f-circRNAs were detected. The distribution of fusion-supporting reads and f-circRNA-supporting reads are shown on the fusion region and back-spliced region respectively.

Additional files

Supplemental_Fig_S1. Eight types of f-circRNAs from 4 fusion genes (*EML4-ALK*, *EWSR1-FLI1*, *KMT2A-MLLT3*, and *PML-RARα*) designed in the simulated RNA-Seq data.

Supplemental_Fig_S2. Computing time of Fcirc in simulated single-end RNA-Seq data.

Supplemental_Fig_S3. Performance comparison of the different gene fusion detection tools in real data PRJNA350335.

Supplemental_Tab_S1. Fusion transcripts detection tools.

Supplemental_Tab_S2. Artificially designed fusion transcripts in simulated data (genome version: hg38).

Supplemental_Tab_S3. Artificially designed f-circRNAs in simulated data (genome version: hg38).

Supplemental_Tab_S4. Precision and recall for synthetic RNA-Seq data.

Supplemental_Tab_S5. F-measure for synthetic RNA-Seq data.

Supplemental_Tab_S6. Computing time for synthetic data.

Supplemental_Tab_S7. The running Parameters of 7 tools.

Supplemental_Tab_S8. Precision and recall for simulated RNA-Seq data.

Supplemental_Tab_S9. F-measure for simulated RNA-Seq data.

Supplemental_Tab_S10. Computing time for paired-end simulated RNA-Seq data.

Supplemental_Tab_S11. *EML4-ALK* fusions identified in real data PRJNA350335.

Supplemental_Tab_S12. F-circRNAs identified by Fcirc in real data.

Supplemental_Tab_S13. Precision, recall and F-measure for real data PRJNA350335.

Supplemental_Tab_S14. Fusion genes identified in real data PRJNA350335 and PRJNA315254.

Figure 1

Click here to access/download;Figure;Fig 1. Fcirc pipeline.pdf

Fig. 1

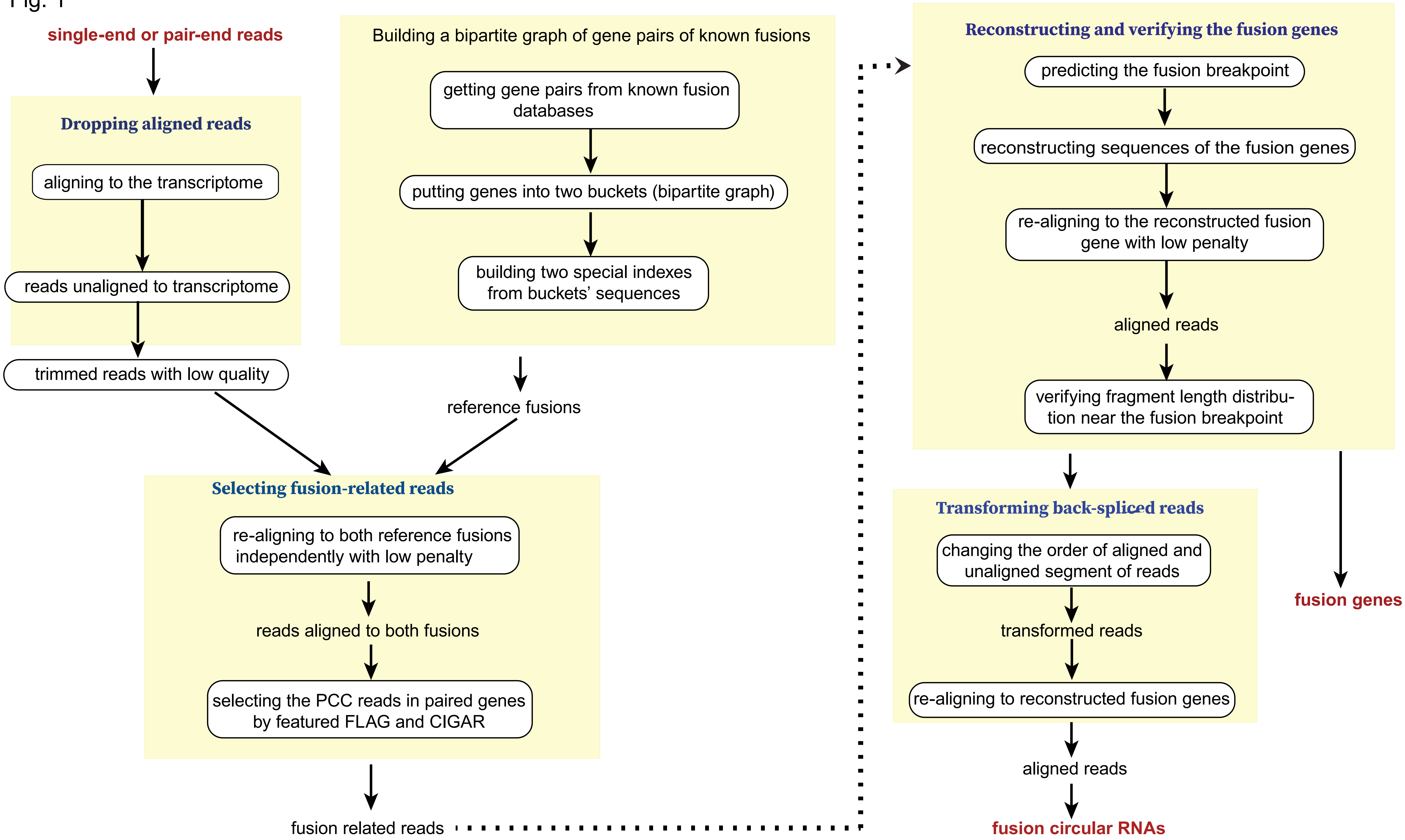

Click here to access/download;Figure;Fig 2. Evaluation in Synthetic RNA-Seq data.pdf

Fig. 2

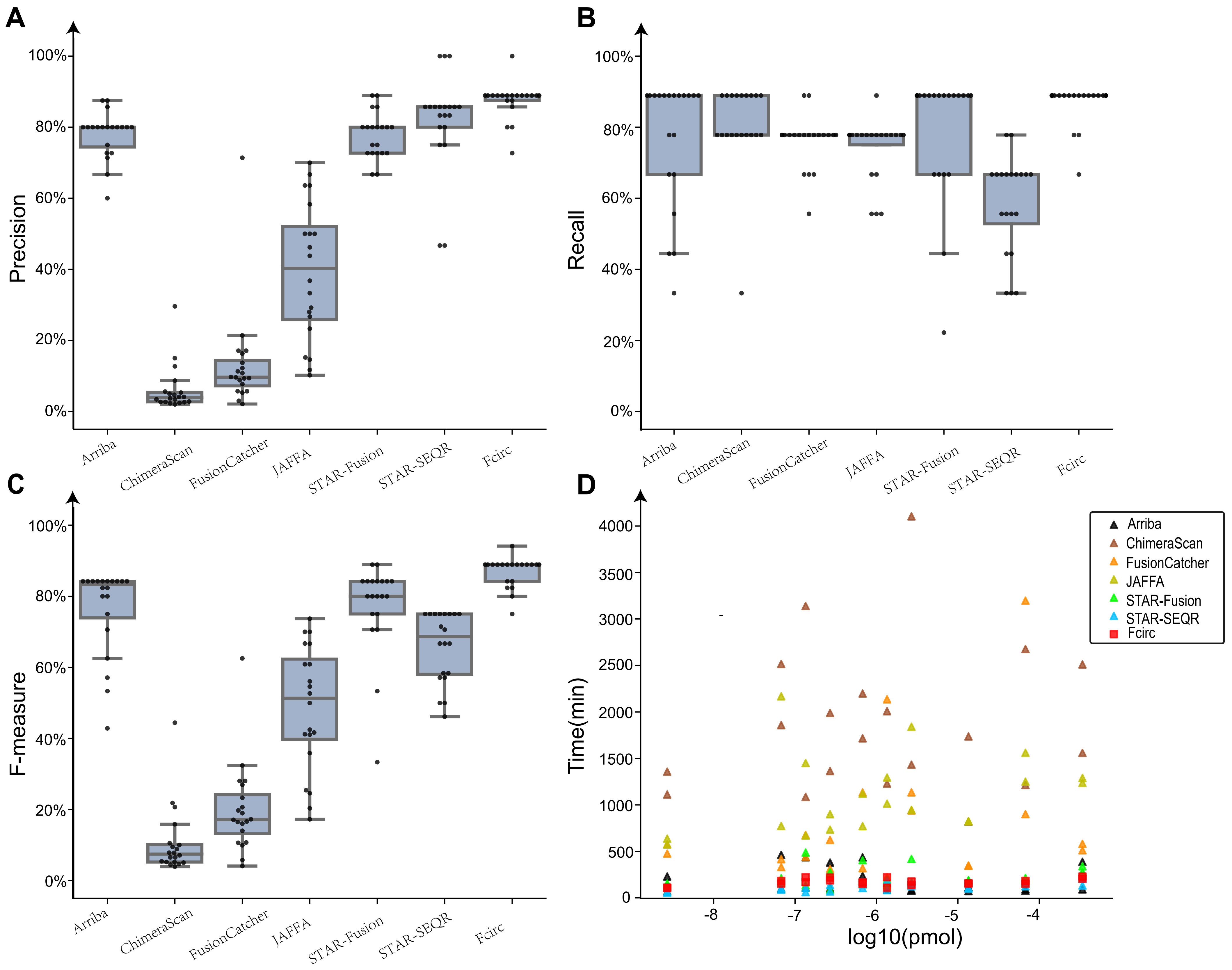

Figure 3

Click here to access/download;Figure;Fig 3. Supporting read identification in real data.pdf

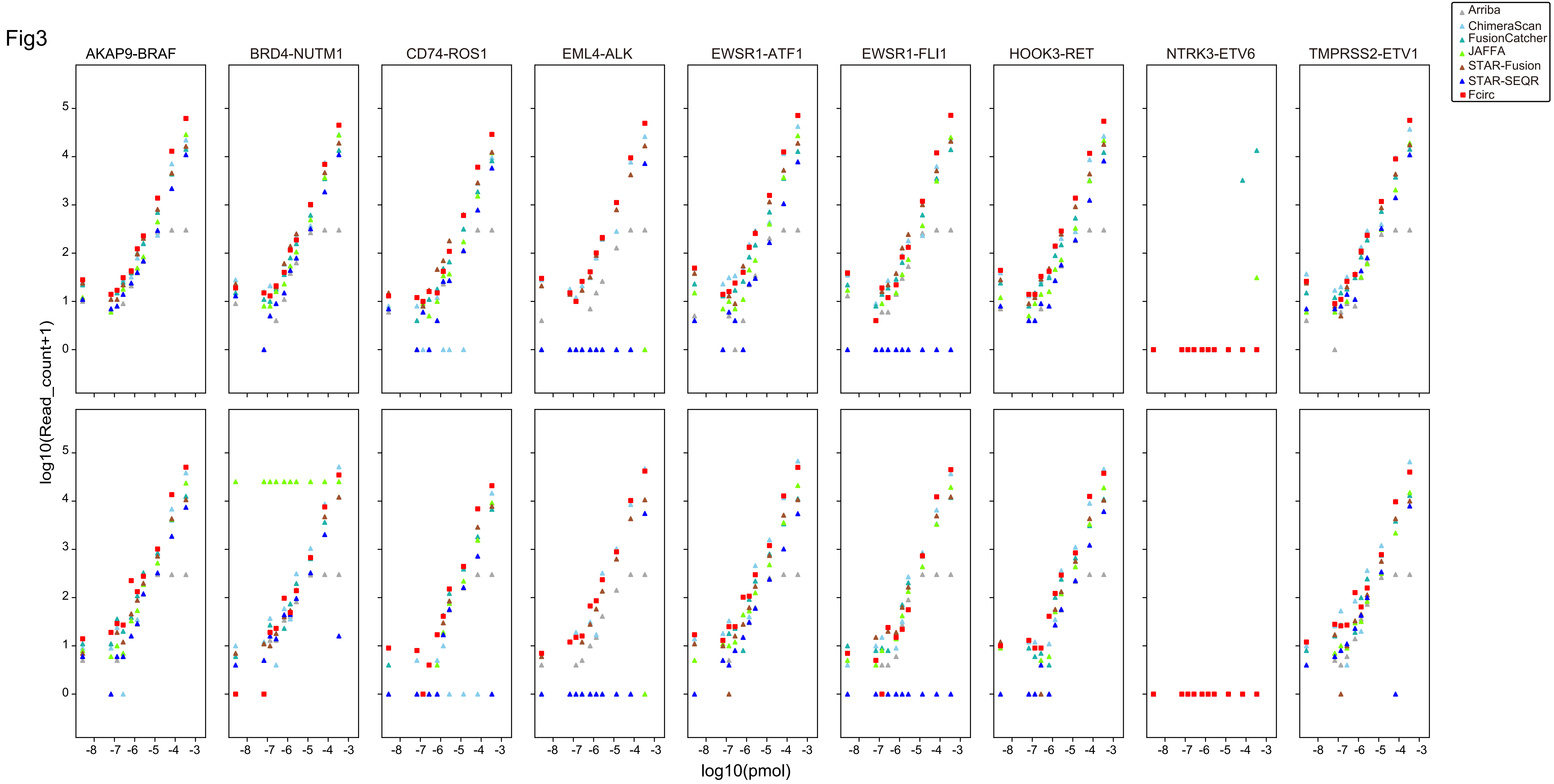

Click here to access/download;Figure;Fig 4. Evaluation in simulated RNA-Seq data.pdf

Fig.4

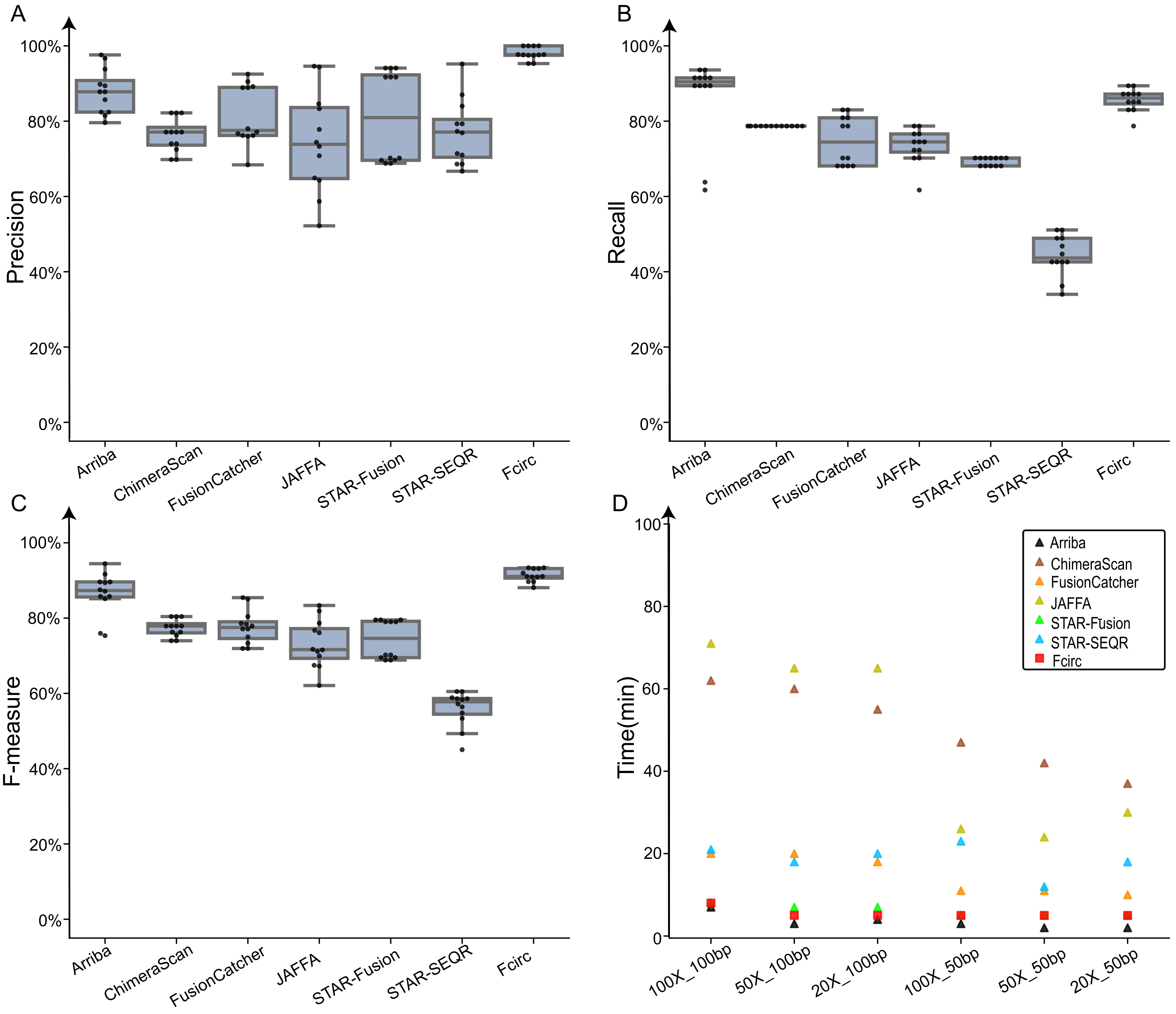

Figure 5 Fig. 5

Click here to access/download;Figure;Fig 5. Heatmap of detected f-circRNAs in simulated data.pdf

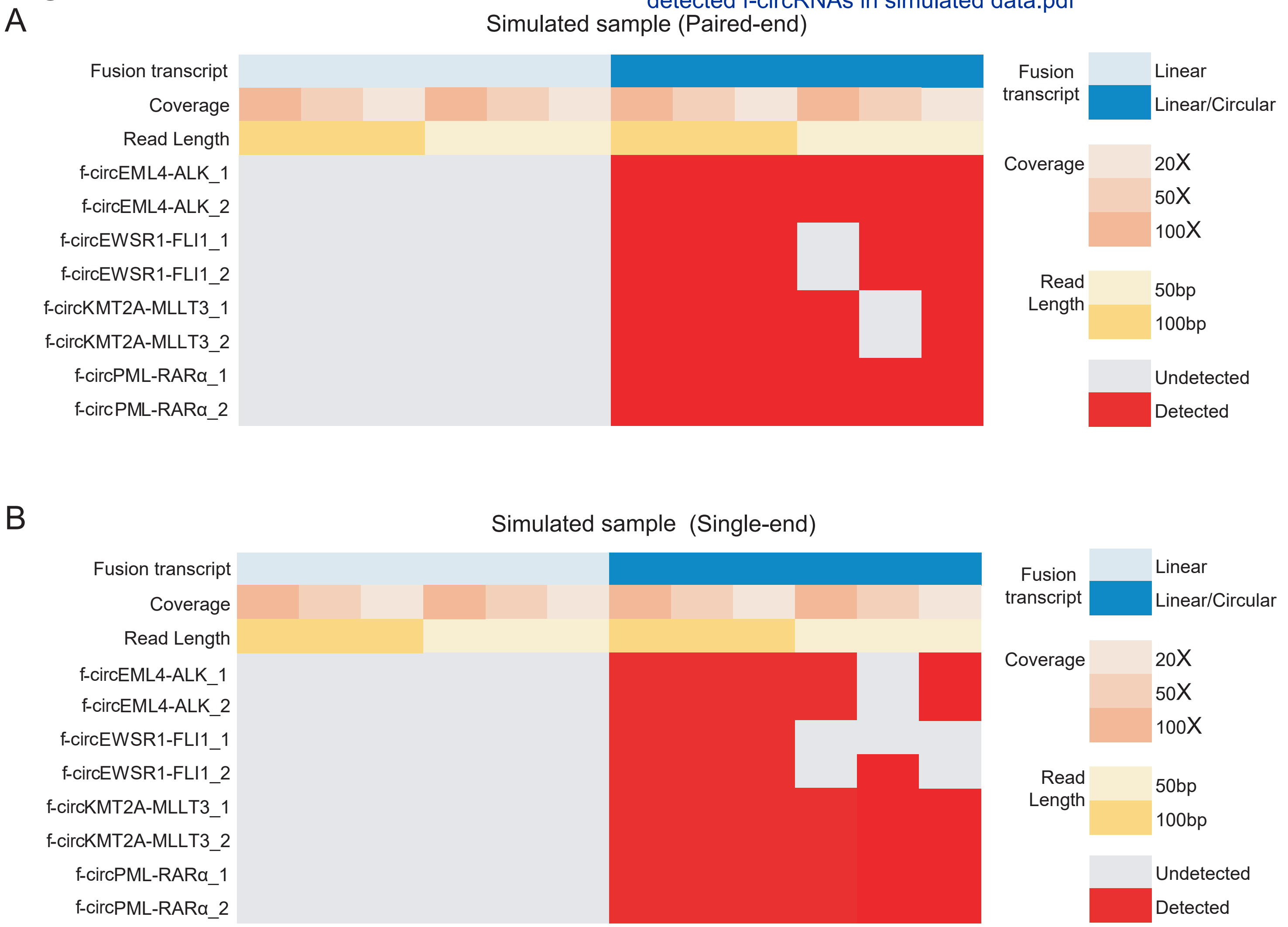

Figure 6

Click here to access/download;Figure;Fig 6. Simulated Dataset Four f-circRNAs.pdf

Fig. 6

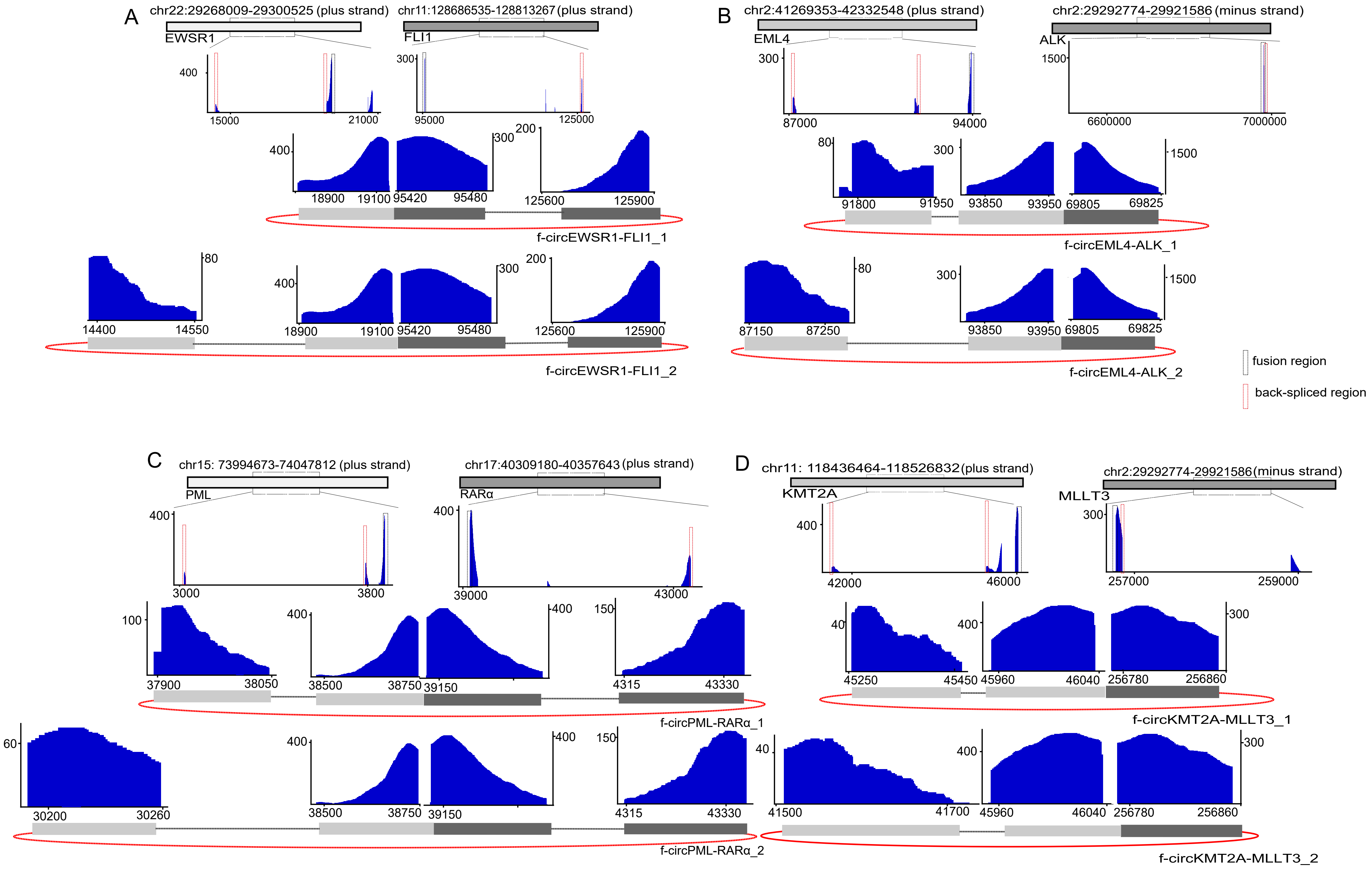

Click here to access/download
Supplementary Material
Supplemental_Fig_S1.pdf

Click here to access/download
Supplementary Material
Supplemental_Fig_S2.pdf

Click here to access/download
Supplementary Material
Supplemental_Fig_S3.pdf

Click here to access/download
Supplementary Material
Supplemental_Tab_S1.xlsx

Click here to access/download
Supplementary Material
Supplemental_Tab_S2.xlsx

Click here to access/download
Supplementary Material
Supplemental_Tab_S3.xlsx

Click here to access/download
Supplementary Material
Supplemental_Tab_S4.xlsx

Click here to access/download
Supplementary Material
Supplemental_Tab_S5.xlsx

Click here to access/download
Supplementary Material
Supplemental_Tab_S6.xlsx

Click here to access/download
Supplementary Material
Supplemental_Tab_S7.xlsx

Click here to access/download
Supplementary Material
Supplemental_Tab_S8.xlsx

Click here to access/download
Supplementary Material
Supplemental_Tab_S9.xlsx

Click here to access/download
Supplementary Material
Supplemental_Tab_S10.xlsx

Click here to access/download
Supplementary Material
Supplemental_Tab_S11.xlsx

Click here to access/download
Supplementary Material
Supplemental_Tab_S12.xlsx

Click here to access/download
Supplementary Material
Supplemental_Tab_S13.xlsx

Click here to access/download
Supplementary Material
Supplemental_Tab_S14.xlsx

Click here to access/download
Supplementary Material
Supplemental_Tab_S15.pdf
