# Supplementary material for: Fcirc: A comprehensive pipeline for the exploration of fusion linear and circular RNAs
Source: Gigascience. 2020 May 29;9(6):giaa054. doi: 10.1093/gigascience/giaa054 (PMC7259471; doi:10.1093/gigascience/giaa054)
Supplement: giaa054_GIGA-D-19-00383_Revision_2 [file giaa054_giga-d-19-00383_revision_2.pdf]

GigaScience

Fcirc: A Comprehensive Pipeline for Exploration of Fusion Linear and Circular RNAs

--Manuscript Draft--

**Manuscript Number:**	GIGA-D-19-00383R2
**Full Title:**	Fcirc: A Comprehensive Pipeline for Exploration of Fusion Linear and Circular RNAs
**Article Type:**	Research
**Funding Information:**	a Mildred-Scheel postdoctoral fellowship from the German Cancer Aid Foundation (70111755) — Dr. Jens Köhler<br>National Natural Science Foundation of China (31771469, 31571363) — Dr. Haiyun Wang<br>National Key Research and Development Program (2017YFC0908500) — Dr. Haiyun Wang
**Abstract:**	Background<br>In cancer cells, fusion genes can produce linear- and chimeric fusion-circular ribonucleic acids (f-circRNAs) which are functional in gene expression regulation and implicated in malignant transformation, cancer progression and therapeutic resistance. For specific cancers, proteins encoded by fusion transcripts have been identified as innovative therapeutic targets (e.g. EML4-ALK). Even though RNA sequencing (RNA-Seq) technologies combined with existing bioinformatics approaches have enabled researchers to systematically identify fusion transcripts, specifically detecting f-circRNAs in cells remains challenging due to their general sparsity, low abundance in cancer cells but also due to imperfect computational methods.<br>Methods and Results<br>We developed the python-based workflow “Fcirc” to identify fusion linear and f-circRNAs from RNA-Seq data with high specificity. We applied Fcirc to three different types of RNA-Seq data scenarios: 1) actual synthetic spike-in RNA-Seq data, 2) simulated RNA-Seq data and 3) actual cancer cell-derived RNA-Seq data. Fcirc showed significant advantages over existing methods regarding both detection accuracy (i.e. precision, recall, F-measure) and computing performance (i.e. lower runtimes).<br>Conclusion<br>Fcirc is a powerful and comprehensive python-based pipeline to identify linear and circular RNA transcripts from known fusion events in RNA-Seq datasets with higher accuracy and shorter computing times compared to previously published algorithms. Fcirc empowers the research community to study the biology of fusion RNAs in cancer more effectively.
**Corresponding Author:**	Haiyun Wang, Ph.D<br>Tongji University<br>Shanghai, Shanghai CHINA
**Corresponding Author Secondary Information:**	
**Corresponding Author's Institution:**	Tongji University
**Corresponding Author's Secondary Institution:**	
**First Author:**	Zhaoqing Cai
**First Author Secondary Information:**	
**Order of Authors:**	Zhaoqing Cai<br>Hongzhang Xue<br>Yue Xu<br>Jens Köhler

Powered by Editorial Manager® and ProduXion Manager® from Aries Systems Corporation

	Xiaojie Cheng
	Yao Dai
	Jie Zheng
	Haiyun Wang, Ph.D
**Order of Authors Secondary Information:**	
**Response to Reviewers:**	GIGA-D-19-00383R2<br>Title: Fcirc: A Comprehensive Pipeline for Exploration of Fusion Linear and Circular RNAs<br><br>EDITORS’ COMMENTS TO THE AUTHOR<br>Your manuscript "Fcirc: A Comprehensive Pipeline for Exploration of Fusion Linear and Circular RNAs" (GIGA-D-19-00383R1) has been assessed by our reviewers. Based on these reports, and my own assessment as Editor, I am pleased to inform you that it is potentially acceptable for publication in GigaScience, once you have carried out some essential revisions suggested by our reviewers.<br>Reply: Thanks so much for your consideration of potential acceptance of our study. We have made the correspond revisions suggested by the reviewers.<br><br>Their reports, together with any other comments, are below. Please also take a moment to check our website at https://www.editorialmanager.com/giga/ for any additional comments that were saved as attachments.<br>In addition, please register any new software application in the bio.tools and SciCrunch.org databases to receive RRID (Research Resource Identification Initiative ID) and biotoolsID identifiers, and include these in your manuscript. This will facilitate tracking, reproducibility and re-use of your tool.<br>Reply: We have registered the IDs in the bio.tools (id: biotools:Fcirc) and SciCruch.org databases (SCR_018090), which are included in the manuscript.<br><br>Once you have made the necessary corrections, please submit a revised manuscript online at:<br>https://www.editorialmanager.com/giga/<br>If you have forgotten your username or password please use the "Send Login Details" link to get your login information. For security reasons, your password will be reset.<br><br>Please include a point-by-point within the 'Response to Reviewers' box in the submission system. Please ensure you describe additional experiments that were carried out and include a detailed rebuttal of any criticisms or requested revisions that you disagreed with. Please also ensure that your revised manuscript conforms to the journal style, which can be found in the Instructions for Authors on the journal homepage. If the data and code has been modified in the revision process please be sure to update the public versions of this too.<br>Reply: Regarding the comments of Reviewer 1, we have discussed the fact that our pipeline only detects known fusions with higher specificity and lower false positive (blue contents in the discussion section). In addition, regarding another point that the reviewer is concerned on, Fcirc allows users to add own fusion gene pairs of interest at their convenience. This functionality of Fcirc is described in the discussion section.<br><br>In this revised manuscript, we also invited a collaborator of our lab, Jens Köhler, to revise our manuscript. The revisions just work on the writing, making the manuscript more logistic and clear, and not change the results and conclusions. To address his contribution on this study, Jens Köhler was added to the co-authors in the revised manuscript. We’ve highlighted this change in the authors list. In addition, we have also improved the quality of the figures.<br><br><br>REVIEWERS' COMMENTS TO THE AUTHOR<br>Reviewer #1: The authors partially answered my comments. I believe it is important that the authors highlight in the discussion the fact that their pipeline will only detect known fusions, which gives it increased specificity.<br>Reply: We appreciate the concerns of Reviewers 1 and have the corresponding discussion regarding this point (blue contents in the discussion section). In our revised

	manuscript, we've discussed that our method differs from other published fusion detection tools by the fact that it requires information on already known gene fusions as reference to build the bipartite graph of gene pairs. Hence, the Fcirc algorithm detects RNAs from known fusion events with increased specifity and lower false positive rate. Accounting for the limitation of depending on known fusions with the cost of losing the ability to identify new fusion genes, Fcirc regularly updates information on newly emerging fusion genes from multiple databases (COSMIC, ChimerDB, TicDB, FARE-CAFÉ, FusionCancer). Moreover, users have the option to add own fusion gene pairs of interest at their convenience.<br><br>Of note some of the software used can also take list of genes involved in fusion as input (Arriba for instance). Have the authors provided the software with the list of the expected genes in the synthetic RNA-Seq experiment, simulated set, and real RNA-Seq? If not this must be performed as this functionality of the other software was omitted.<br>Reply: Thanks so much for this nice comment! Fcirc allows users to add the expected fusion genes as an input. We make this option accounting for the limitation of Fcirc, which depends on known fusions, and also considering a scenario where some researchers may have some specific fusions they are interested in. So users have the option to add own fusion gene pairs of interest at their convenience. But in the performance evaluation using the synthetic RNA-Seq, simulated set, and actual RNA-Seq data, we do not provide Fcirc with the list of the expected genes to avoid the bias of the evaluation.
**Additional Information:**	
**Question**	**Response**
Are you submitting this manuscript to a special series or article collection?	No
**Experimental design and statistics**<br><br>Full details of the experimental design and statistical methods used should be given in the Methods section, as detailed in our Minimum Standards Reporting Checklist. Information essential to interpreting the data presented should be made available in the figure legends.<br><br>Have you included all the information requested in your manuscript?	Yes
**Resources**<br><br>A description of all resources used, including antibodies, cell lines, animals and software tools, with enough information to allow them to be uniquely identified, should be included in the Methods section. Authors are strongly encouraged to cite Research Resource Identifiers (RRIDs) for antibodies, model organisms and tools, where possible.	Yes

Have you included the information requested as detailed in our Minimum Standards Reporting Checklist?	
**Availability of data and materials**<br><br>All datasets and code on which the conclusions of the paper rely must be either included in your submission or deposited in publicly available repositories (where available and ethically appropriate), referencing such data using a unique identifier in the references and in the “Availability of Data and Materials” section of your manuscript.<br><br>Have you have met the above requirement as detailed in our Minimum Standards Reporting Checklist?	Yes

Powered by Editorial Manager® and ProduXion Manager® from Aries Systems Corporation

Manuscript with DOI

Click here to access/download;Manuscript;main_final_200409 with DOI.docx

Fcirc: A Comprehensive Pipeline for the Exploration of Fusion Linear and Circular RNAs

Zhaoqing Cai[1,#], Hongzhang Xue[2,1#], Yue Xu[1], Jens Köhler[3], Xiaojie Cheng[1], Yao Dai[1], Jie Zheng[1], Haiyun Wang[1,*]

[1]*School of Life Sciences and Technology, Tongji University, Shanghai 200092, China*

[2]*School of Life Sciences and Biotechnology, Shanghai Jiao Tong University, Shanghai 200240, China*

[3]*Department of Medical Oncology, Dana-Farber Cancer Institute, Boston, MA, 02215, USA*

E-mail: 1731473@tongji.edu.cn (Cai Z), xuehzh95@foxmail.com (Xue H), 1731490@tongji.edu.cn (Xu Y), jens_kohler@dfci.harvard.edu (Köhler J), siyecaodelvlanzi@163.com (Cheng X), daiyao0808@sina.com (Dai Y), 1931524@tongji.edu.cn (Zheng J), wanghaiyun@tongji.edu.cn (Wang H)

[#] Equal contribution.

[*] Corresponding author.

Abstract

Background

In cancer cells, fusion genes can produce linear- and chimeric fusion-circular ribonucleic acids (f-circRNAs) which are functional in gene expression regulation and implicated in malignant transformation, cancer progression and therapeutic resistance. For specific cancers, proteins encoded by fusion transcripts have been identified as innovative therapeutic targets (e.g. *EML4-ALK*). Even though RNA sequencing (RNA-Seq) technologies combined with existing bioinformatics approaches have enabled researchers to systematically identify fusion transcripts, specifically detecting f-circRNAs in cells remains challenging due to their general sparsity, low abundance in cancer cells but also due to imperfect computational methods.

Methods and Results

We developed the python-based workflow "Fcirc" to identify fusion linear and f-circRNAs from RNA-Seq data with high specificity. We applied Fcirc to three different types of RNA-Seq data scenarios: 1) actual synthetic spike-in RNA-Seq data, 2) simulated RNA-Seq data and 3) actual cancer cell-derived RNA-Seq data. Fcirc showed significant advantages over existing methods regarding both detection accuracy (i.e. precision, recall, F-measure) and computing performance (i.e. lower runtimes).

Conclusion

Fcirc is a powerful and comprehensive python-based pipeline to identify linear and circular RNA transcripts from known fusion events in RNA-Seq datasets with higher accuracy and

shorter computing times compared to previously published algorithms. Fcirc empowers the research community to study the biology of fusion RNAs in cancer more effectively.

Keywords: Fcirc; Fusion linear RNA; Fusion circRNA; Performance benchmarks

Background

Various events like gene mutations, gene rearrangements and chromosomal fragile sites are able to induce the formation of fusion genes in the genome of cancer cells [1-6]. These fusion genes can generate linear or fusion circular ribonucleic acids (f-circRNAs) - the latter via back-splicing of exons. F-circRNAs are functional in gene expression regulation and are implicated in malignant transformation, cancer cell survival and therapeutic resistance [7]. Apart from their relevance for cancer cell biology, f-circRNAs also serve as promising biomarker candidates in liquid biopsies due to their increased stability relative to linear transcripts [8]. Furthermore, proteins encoded by fusion genes represent innovative therapeutic targets in some cancers thus indicating that the still relatively young field of fusion RNA biology harbors a great potential for drug development. Crizotinib, for example, a tyrosine kinase inhibitor, was FDA-approved in 2013 for the treatment of patients with non-small cell lung cancer (NSCLC) harbouring *EML4-ALK* rearrangements[9]. Therefore, accurate profiling of fusion linear and circular RNAs is of high scientific interest and provides the basis for functional studies in cancer. Although recent advances in high-throughput RNA-Seq data acquisition have enabled researchers to detect fusion transcripts [10-15] and circRNAs [16-18], the currently available tools for fusion detection still yield a high false discovery rate [19], and current bioinformatics methods cannot be used to identify the whole spectrum of f-circRNAs arising from a specific fusion gene [20].

Therefore, in the present study, we developed "Fcirc", a comprehensive, accurate and free of charge pipeline to analyze RNA-Seq data for linear and circular RNAs transcribed from fusion genes.

Materials and Methods

Datasets used in this study

1) Synthetic spike-in actual RNA-Seq data

To evaluate the performance of different tools for fusion RNA analysis, we took advantage of RNA-Seq data from a study performed by Tembe et al. [21]. In this study, equimolar amounts of nine synthetic poly-adenylated gene fusion RNA transcripts were pooled and titrated into total RNA of COLO-829 melanoma cells at ten different concentrations with two replicates for each sample: *EWSR1-ATF1*, *TMPRSS2-ETV1*, *EWSR1-FLI1*, *NTRK3-ETV6*, *CD74-ROS1*, *HOOK3-RET*, *EML4-ALK*, *AKAP9-BRAF*, and *BRD4-NUTM1*. The sequencing data (Illumina HiSeq 2500 system) was made available in FASTQ format in the Short Read Archive under accession number SRP043081 and allows researchers to validate novel algorithms for gene fusion detection in a comparative manner.

2) Simulated RNA-Seq data

The simulator art_illumina function in ART [22] was applied to simulate RNA-Seq data. We used the RNA-Seq reads from normal pulmonary microvascular endothelial cells in the National Center for Biotechnology Information (NCBI) Sequence Read Archive (SRA) database SRR349695 [23] as background and plugged simulated fusion reads into the background. Two types of fusion reads were designed: 1) those derived from linear transcripts and 2) those derived from pooled linear and circular transcripts. A total of 47 fusions (Suppl. Table 1) with high, median and low frequency in cancers were randomly selected from the Catalogue Of Somatic Mutations In Cancer (COSMIC) database [24]. The linear fusion reads were artificially generated based on the breakpoint information by joining the upstream and downstream transcript fragments. Eight fusion circRNAs (Suppl. Fig. 1, Suppl. Table 2) were

generated according to previous reports [7, 25]. In order to simulate more linear than circular fusion transcripts at a gene locus, we plugged 2.5 times as many linear fusion reads into the background as circular fusion reads. Different sequencing coverages (20, 50 and 100x) were simulated each with two read lengths of 50 and 100 bp.

3) Actual cancer-cell derived RNA-Seq data

Actual cancer-cell derived RNA-Seq data for the identification of f-circRNA was obtained from the BioProject database (accession IDs PRJNA350335 and PRJNA315254). Whereas PRJNA350335 includes sequencing information on nine lung cancer samples of H3122 cell line harbouring the *EML4-ALK* fusion gene [26], PRJNA315254 includes a total of nine acute leukemia samples among them NB4 (n=3), THP1 (n=1) and primary patient-derived (n=5) cell lines harbouring the *PML-RARα* fusion gene [7].

The Fcirc pipeline workflow

The "Fcirc" analysis pipeline includes five major steps (Fig. 1) and the baseline data input are single-end or paired-end RNA-Seq datasets in FASTQ format. Both, raw and cleaned data is acceptable e.g. after adapter cutting or poor-quality trimming.

Step 1) Dropping aligned reads

Reads were aligned to a reference transcriptome with HISAT2 [27] using default parameters. After the first alignment, the aligned reads were dropped by samtools [28] and unaligned reads were kept for further analysis. For single-end RNA-Seq data, reads with a FLAG value of 4 in the Sequence Alignment/Map format (SAM) file were selected (-f 4) and converted into files in FASTQ format. For paired-end RNA-Seq data, reads without a FLAG value of 2 in the

SAM file were selected (-F 2), and that either segment of a read was unaligned and then converted into a file in FASTQ format.

Step 2) Building of a bipartite graph of gene pairs of known fusions

Gene pairs of known fusion genes were manually curated from multiple databases, including COSMIC [24], ChimerDB [29], TicDB [30], FARE-CAFE [31] and FusionCancer [32] and the gene sequences of known gene fusions were downloaded from the Ensembl Genome Browser [33]. With this information, we built a "bipartite graph" (also called a "bigraph") of known fusion gene-pairs. In a bipartite graph vertices (representing individual genes in our study) can be divided into two disjoint and independent sets **U** and **V** in a way that every edge (representing fusion events between individual genes in our study) connects a vertex in **U** to one in **V**. In our case a bipartite graph was possible to be generated because the genes involved in the fusion events did not form a ring of odd vertices. To reduce the computational complexity and time required to search for multiple gene-spanning reads, genes involved in the fusion event were divided into two independent sets according to the bipartite graph theory. For example, in the case of the fusion genes *EML4-ALK* and *NPM1-ALK*, *EML4* and *NPM1* were included in the same set (U) while *ALK* was included in the independent set (V).

Step 3) Selecting fusion-related reads

In the next step, the unaligned reads were independently re-aligned to two sets of fusion gene sequences with low penalty. We decreased the maximum and minimum penalty for soft-clipping (--sp 1, 1) and minimum alignment score (--score-min L, 0, -0.8). Other scoring parameters were set as default. After this re-alignment, reads with partial sequence alignment to fusion genes were selected. For single-end RNA-Seq data, reads without a FLAG value of 4

(-F 4) and for paired-end RNA-Seq data, reads with a FLAG value of 4, not 8, or 8, not 4 or 12 in the SAM file were selected (-f 4 -F 8 or -F 4 -f 8 or -f 12), respectively, ensuring that at least one read segment was aligned. Reads with paired chiastic clipping (PCC) signal were defined as fusion-related reads. For instance, if a segment of a read was simultaneously aligned to *EML4* with the same FLAG and CIGAR 40S60M values and to *ALK* with FLAG 4 and CIGAR 40M60S, this suggested that one segment of *EML4* and the rest from *ALK* were on the same strand.

Step 4) Reconstructing and verifying the fusion genes

In the next step, the fusion breakpoint was determined. Therefore, we assumed that fusion-related reads were more likely to cover the respective fusion breakpoint and inferred the exact location from the majority of junction-supported reads. Subsequently, the fusion gene sequence was reconstructed around this predicted fusion breakpoint and the alignment of reads was recalibrated by re-aligning reads to the reconstructed fusion gene with low penalty. To evaluate our assumption, that fusion-related reads uniformly covered the fusion breakpoint, they were split into two groups (left and right fragments) in relation to the respective breakpoint. Then, the Wilcoxon Sign Rank Test was used to evaluate the read distribution by comparing the length of the left and right fragments.

Step 5) Transforming back-spliced reads

Circular RNAs transcribed from fusion genes were detected by searching for back-spliced reads. To improve the alignment of back-spliced reads with the reconstructed fusion gene, we changed the order of aligned and unaligned segments of some back-spliced reads to transform back-spliced reads to forward-spliced reads. The transformed reads were then re-aligned to

the reconstructed fusion gene to evaluate whether they were truly back-spliced. Those reads covering a back-spliced junction indicated that they were attributable to f-circRNA.

Fcirc data output format

The resulting output format of Fcirc are tables of fusion linear transcripts and of f-circRNAs as well as SAM files for easier visualization of read distribution on the respective fusions.

Performance benchmarking and evaluation criteria

Fcirc and six previously published fusion detection methods (Suppl. Table 3), including Arriba v1.1.0 [34], ChimeraScan v0.4.5 [14], FusionCatcher v1.00 [12], JAFFA v1.09 [15], STAR-Fusion v1.8.1 [13] and STAR-SEQR v0.6.7 [35], were applied to the synthetic spike-in actual RNA-Seq data, simulated data and actual cancer cell-derived data. To accurately evaluate and compare these tools, we required 1) the number of fusion-supporting reads to be at least 3, and 2) read-through transcripts to be removed i.e. two genes located on the same chromosome less than 100,000 bp apart. The computational efficiency of each tool was evaluated by several benchmarking criteria including precision, recall and F-measure which were defined as follows:

$$precision = TP/(TP + FP)$$

$$recall = TP/(TP + FN)$$

$$F - measure = precision * recall * 2/(precison + recall)$$

TP, FP and FN represent the true positives, false positives and false negatives, respectively. The F-measure simultaneously considers the effect of precision and recall. We also evaluated the number of supporting reads that were identified by the different methods and which reflect the ability to robustly detect the gene fusion. The final benchmark was the required computing time assuming a computational environment based on Ubuntu Linux with Intel Xeon E5-2620

v4 CPU@ 2.10GHz. Four CPU cores were used for each tool and the running parameters for each tool are shown in Suppl. Table 4.

Results

Evaluation of gene fusions in actual and simulated RNA-Seq datasets from synthetic spike-in experiments

To compare the performance parameters (i.e. precision, recall and F-measure) of Fcirc with those of other methods, we took advantage of RNA-Seq data from spike-in experiments which included nine synthetic cancer-associated fusion genes (*EWSR1-ATF1*, *TMPRSS2-ETV1*, *EWSR1-FLI1*, *NTRK3-ETV6*, *CD74-ROS1*, *HOOK3-RET*, *EML4-ALK*, *AKAP9-BRAF*, and *BRD4-NUTM1)* [21]. Fcirc achieved not only the highest but also more consistent (small standard deviation (0.05)) precision (87.50%) compared to STAR-SEQR (81.90%) and Arriba (78.00%) (Fig 2A, Suppl. Table 5-1). ChimeraScan (6.10%) and FusionCatcher (13.4%) exhibited low precision values thus indicating a high rate of false positive results for predictions with these methods. In addition, Fcirc achieved higher recall values (86.68%) than all other methods (ChimeraScan=80.57%, STAR-Fusion=78.90%, Arriba=76.68%, FusionCatcher=76.14%, JAFFA=73.92% and STAR-SEQR=58.35%) (Fig. 2B, Suppl. Table 5-2) and greater F-measures (0.86), indicating a better performance for balancing precision and recall **(**Fig. 2C, Suppl. Table 6). Finally, Fcirc required less computing time than most of the other methods (Fig. 2D, Suppl. Table 7).

In the next step, we calculated the number of fusion-supporting junctional reads for the different methods with respect to ten different spike-in concentrations of the pooled synthetic gene fusion RNAs (n=9, two replicates each). Fcirc (red squares) not only identified the highest number of supporting reads but also had a very high accuracy for different spike-in

concentrations indicated by the increasing number of identified supporting reads of a given gene fusion (Fig. 3). The NTRK3-ETV6 fusion RNA construct was basically undetectable by all applied methods.

We also evaluated the performance of all seven algorithms for the simulated paired-end data including both, linear and pooled linear/circular transcripts. Again, Fcirc achieved higher and more consistent precision (98.02%) than the other methods, with a high recall (85.64%) that was only second to Arriba (86.36%) (Fig. 4A and B, Suppl. Table 8). Fcirc also generated the highest and most consistent F-measures (0.91) in all of the simulated data, followed by Arriba (0.86) (Fig. 4C, Suppl. Table 9) and both methods, Fcirc and Arriba required less computing time compared to the other methods (Fig. 4D). For the single-end (Suppl. Fig. 2) and paired-end data analysis (Suppl. Table 10), Fcirc required computing times around or below 5 minutes depending on the RNA-Seq data settings.

Evaluation of Fcirc performance to detect f-circRNAs in simulated RNA-Seq data

To evaluate the ability of Fcirc to identify f-circRNA, we designed reads of eight fusion circRNAs according to previous reports and plugged them into RNA-Seq data from normal pulmonary microvascular endothelial cells. We designed two types of RNA-Seq data: 1) a control dataset which contained only linear fusion transcripts, and 2) a dataset which contained pooled linear/circular fusion transcripts. Furthermore, single-end and paired-end RNA-Seq data, as well as different sequencing coverages (20, 50, 100x) and read lengths (50 and 100 bp) were simulated. In paired-end samples, Fcirc successfully detected all eight types of f-circRNAs from RNA plugged with pooled linear/circular fusion transcripts, whereas - as expected - no f-circRNAs were detected when only the linear fusion transcripts were present (Fig. 5A). The Fcirc alghorithm also showed high accuracy in simulated single-end samples

(Fig 5B). Overall, all f-circRNAs were identified in paired-end and single-end samples with a read length of 100 bp whereas more f-circRNAs were identified in the paired-end versus single-end samples with a read length of 50 bp and the same coverage. Eight f-circRNAs transcribed from four fusion genes which were identified in the paired-end sample dataset (100x coverage, 100 bp read length) are visualized in Fig. 6: a) *EWSR1-FLI1*, b) *EML4-ALK*, c) *PML-RARα*, d) *KMT2A-MLLT3*.

Identification of f-circRNAs in actual RNA-Seq data

In the next step, we sought to identify f-circRNAs based on actual BioProject RNA-Seq data at the example of H3122 cells, a non-small cell lung cancer cell line harboring the *EML4-ALK* fusion (BioProject ID PRJNA350335) and of various acute leukemia samples with *PML-RARα* fusion gene (BioProject ID PRJNA315254). We applied the Fcirc algorithm to the PRJNA350335 dataset in order to detect linear and circular fusion transcripts. Fcirc successfully identified *EML4-ALK* fusions in all nine H3122 samples at the specific previously reported fusion breakpoint (Suppl. Table 11, the number of supporting reads per cell line sample is indicated) [26]. We also successfully identified the previously reported *EML4-ALK* fusion-derived f-circRNA (Suppl. Table 12) [8, 36].

Next, we compared the performance of all seven algorithms based on the PRJNA350335 dataset which does not provide any information on the truly present fusion genes itself. Therefore, we defined fusions as true positives if they were detected by at least four tools with more than ten supporting reads each, and then compared the performance of each method in detecting the presumably true positive fusions. Comparable to the spike-in experiments, Fcirc (100%) achieved the highest precision compared to other methods (Suppl. Fig. 3A, Suppl. Table 13-1), whereas it lacked behind ChimeraScan (100%), STAR-Fusion (100%) and

STAR-SEQR (100%) regarding the recall rate (Suppl. Fig. 3B, Suppl. Table 13-2). Fcirc also had higher F-measures (0.815) than most other tools being only second to the STAR-SEQR algorithm (0.889) (Suppl. Fig. 3C, Suppl. Tab 13-3). We detected the known *KMT2A-MLLT3* (*MLL-AF9*) and *PML-RARα* fusions in the BioProject dataset PRJNA315254 [7]. Supplemental Table 14 summarizes all fusion genes identified in datasets PRJNA350335 and PRJNA315254 and indicates the number of supporting reads for each individual fusion gene. Interestingly, different f-circRNA isoforms were detected for the *PML-RARα* fusion (Suppl. Table 12) with the total numbers of isoforms being dependent on the computational assumptions - e.g. 18 and 8 isoforms were detected in NB4 cells when the cutoff of read count numbers supporting f-circRNAs was changed to one or two (RNA-Seq data sample ID SRR3239817).

Discussion

Fusion linear and circular transcripts (f-circRNA) are RNAs which are derived from rearranged genome translocations [1-6]. Even though the precise role of many of these RNAs remains elusive, it becomes increasingly evident that some of them are functional in gene expression regulation and therefore implicated in malignant transformation, cancer cell survival and therapeutic resistance [7]. The example of crizotinib, an FDA-approved tyrosine kinase inhibitor for the treatment of *EML4-ALK* rearranged NSCLC, shows, that proteins encoded by fusion transcripts can also be harnessed as innovative drug targets[9]. This emphasizes the need for methods to accurately determine linear and f-circRNA profiles within cancer cells. Currently, numerous RNA-Seq datasets are publicly available which can be used to predict linear and f-circRNAs. However, it remains a significant challenge to detect

specifically f-circRNA transcripts due to their low frequency and low expression abundance within cancer cells. Furthermore, RNA-Seq data in general suffers from heavy background noise thus increasing the rate of false positive results.

Therefore, here, we developed the python-based pipeline "Fcirc" to overcome these drawbacks and to enable researchers to accurately identify and quantify linear and circular (f-circRNAs) fusion transcripts from RNA-Seq data. Fcirc differs from other published fusion detection tools such as Arriba [34], ChimeraScan [14], JAFFA [15], FusionCatcher [12], STAR-Fusion [13] and STAR-SEQR [35] by the fact that it requires information on already known gene fusions as reference to build the bipartite graph of gene pairs (Step 2 of the algorithm). Hence, the Fcirc algorithm - despite coming at the cost of losing the ability to identify new fusion genes - detects RNAs from known fusion events with higher specificity and lower false positive rate. Fcirc accounts for the limitation of depending on known fusions by regularly updating information on newly emerging fusion genes from multiple databases (COSMIC, ChimerDB, TicDB, FARE-CAFÉ, FusionCancer). Users furthermore have the option to add own fusion gene pairs of interest at their convenience.

In a benchmarking effort, we compared the performance of Fcirc with the six above mentioned fusion detection tools (tool characteristics are summarized in Suppl. Table 3) on the basis of three different RNA-Seq data scenarios: 1) actual RNA-Seq data from synthetic spike-in experiments [21], 2) simulated RNA-Seq data and 3) actual cancer-cell derived RNA-Seq data. The analyses in these three scenarios showed that Fcirc offers higher precision compared to all other algorithms (Fig. 2A and 4A, Suppl. Fig. 3A), very high recall qualities (Fig. 2B and 4B, Suppl. Fig. 3B) and F-measures (Fig. 2C and 4C, Suppl. Fig. 3C), but also high numbers of fusion-supporting reads (Fig. 3) as well as reduced computing times (Fig. 2D

and 4D, Suppl. Fig. 2). Especially for the actual RNA-Seq dataset with synthetic fusion RNA spike-in (RNA-Seq scenario 1), the problem of high false positive rates became evident for some of the other tools, as only 6.1% and 13.4% of fusion transcripts predicted by ChimeraScan and FusionCatcher were true positives, respectively, whereas Fcirc achieved a true positive rate of 87.5% (Fig. 2A, Suppl. Table 5). Contrariwise, the recall rate was slightly lower for Fcirc compared to the other tools for the actual cancer cell-derived RNA-Seq datasets (RNA-Seq scenario 3), which is likely due to presence of unknown fusions.

Fcirc furthermore detected f-circRNAs with high reliability and accuracy in simulated datasets with paired-end (Fig. 5A) and single-end samples (Fig. 5B) under different coverage and read length conditions as well as in actual cancer-cell derived RNA-Seq datasets (e.g. *EML4-ALK* and *PML-RARα*) (Suppl. Fig. 1). These results confirm previous reports on gene fusion events in multiple NSCLC and acute leukemia cell lines that were used for our analysis [7, 8, 36]. Interestingly, Fcirc identified about ten different f-circRNA transcripts for the *PML-RARα* fusion gene in NB4 leukemia cells (depending on the computational assumptions), which warrants further investigation and biological characterization (Suppl. Table 12).

In conclusion, our study provides an insightful comparison of different fusion detection tools and suggests Fcirc as a powerful tool to detect linear and circular RNA transcripts of known fusion genes with high specificity in RNA-Seq datasets. Fcirc's reduced computing time will expedite the analysis of very large data sets and therefore improve our future understanding of the impact of gene fusion-related transcripts on cancer biology.

Availability of Supporting Source Code and Requirements

- Project name: Fcirc: A Comprehensive Pipeline for the Exploration of Fusion, Linear and Circular RNAs
- Project home page: https://github.com/WangHYLab/fcirc
- Operating system(s): Ubuntu 16.04/18.04, MacOS
- Programming language: Python
- Other program requirements: hisat2, samtools, numpy, scipy, pysam
- License: Massachusetts Institute of Technology (MIT, Cambridge, USA)
- Bio.tools id: biotools:Fcirc
- Project RRID: SCR_018090

Availability of Supporting Data and Materials

Synthetic spike-in real RNA-Seq data was obtained from the Short Read Archive under the accession number SRP043081 [21]. Actual RNA-Seq data was obtained from the BioProject with accession IDs PRJNA350335 and PRJNA315254. Simulated RNA-Seq data was generated as described in the Methods section, and reference information of fusion transcripts and of f-circRNAs are shown in the Additional files section (Suppl. Table 2 and 3). Other data further supporting this work is openly available in the *GigaScience* repository, GigaDB [37].

Abbreviations

RNA: ribonucleic acid; RNA-Seq: ribonucleic acid sequencing; f-circRNA: fusion-circular ribonucleic acid; circRNA: circular ribonucleic acid; SAM: Sequence Alignment/Map format; PCC: paired chiastic clipping; TP: true positive; FP: false positive; FN: false negative;

SRA: Sequence Read Archive; NCBI: National Center for Biotechnology Information; COSMIC: Catalogue Of Somatic Mutations In Cancer

Competing interests

The authors declare no competing interests.

Authors' contributions

HW conceived the hypothesis. ZC, HX, XC, YD, and JZ designed and performed the pipeline workflow and the analyses. HW, JK, HX and ZC interpreted the results and wrote the manuscript.

Funding

This work was supported by grants from the National Natural Science Foundation of China (31771469 and 31571363 to HW), a grant from the National Key Research and Development Program (2017YFC0908500 to HW) and a Mildred-Scheel postdoctoral fellowship from the German Cancer Aid Foundation (70111755 to JK).

References

1. Lebeau MM and Rowley JD. Cancer biology: Heritable fragile sites in cancer. Nature. 1984;308 5960:607-8.
2. Stratton MR, Campbell PJ and Futreal PA. The cancer genome. Nature. 2009;458 7239:719-24.
3. Huebner K. Molecular biology: DNA fragility put into context. Nature. 2011;470 7332:46-7.
4. Coquelle A, Toledo F, Stern S, Bieth A and Debatisse M. A New Role for Hypoxia in Tumor Progression: Induction of Fragile Site Triggering Genomic Rearrangements and Formation of Complex DMs and HSRs. Molecular Cell. 1998;2 2:259-65.
5. Novo FJ and Vizmanos JL. Chromosome translocations in cancer: computational evidence for the random generation of double-strand breaks. Trends in Genetics. 2006;22 4:193-6.
6. Imielinski M and Ladanyi M. Fusion oncogenes—genetic musical chairs. Science. 2018;361 6405:848-9.
7. Guarnerio J, Bezzi M, Jeong JC, Paffenholz SV, Berry K, Naldini MM, et al. Oncogenic Role of Fusion-circRNAs Derived from Cancer-Associated Chromosomal Translocations. Cell. 2016;165 2:289-302. doi:10.1016/j.cell.2016.03.020.
8. Tan S, Gou Q, Pu W, Guo C, Yang Y, Wu K, et al. Circular RNA F-circEA produced from EML4-ALK fusion gene as a novel liquid biopsy biomarker for non-small cell lung cancer. Cell research. 2018;28 6:693-5. doi:10.1038/s41422-018-0033-7.
9. Shaw AT, Kim DW, Nakagawa K, Seto T, Crino L, Ahn MJ, et al. Crizotinib versus chemotherapy in advanced ALK-positive lung cancer. N Engl J Med. 2013;368 25:2385-94. doi:10.1056/NEJMoa1214886.
10. Wang K, Singh D, Zeng Z, Coleman SJ, Huang Y, Savich GL, et al. MapSplice: accurate mapping of RNA-seq reads for splice junction discovery. Nucleic Acids Res. 2010;38 18:e178. doi:10.1093/nar/gkq622.
11. Kim D and Salzberg SL. TopHat-Fusion: an algorithm for discovery of novel fusion transcripts. Genome Biol. 2011;12 8:R72. doi:10.1186/gb-2011-12-8-r72.
12. Nicorici D, Şatalan M, Edgren H, Kangaspeska S, Murumägi A, Kallioniemi O, et al. FusionCatcher – a tool for finding somatic fusion genes in paired-end RNA-sequencing data. bioRxiv. 2014:011650. doi:10.1101/011650.
13. Haas BJ, Dobin A, Stransky N, Li B, Yang X, Tickle T, et al. STAR-Fusion: Fast and Accurate Fusion Transcript Detection from RNA-Seq. bioRxiv. 2017:120295. doi:10.1101/120295.
14. Iyer MK, Chinnaiyan AM and Maher CA. ChimeraScan: a tool for identifying chimeric transcription in sequencing data. Bioinformatics. 2011;27 20:2903-4. doi:10.1093/bioinformatics/btr467.
15. Davidson NM, Majewski IJ and Oshlack A. JAFFA: High sensitivity transcriptome-focused fusion gene detection. Genome Med. 2015;7 1:43. doi:10.1186/s13073-015-0167-x.
16. Gao Y, Wang J and Zhao F. CIRI: an efficient and unbiased algorithm for de novo circular RNA identification. Genome Biol. 2015;16:4. doi:10.1186/s13059-014-0571-3.
17. Szabo L, Morey R, Palpant NJ, Wang PL, Afari N, Jiang C, et al. Statistically based splicing detection reveals neural enrichment and tissue-specific induction of circular RNA during human fetal development. Genome Biol. 2015;16:126. doi:10.1186/s13059-015-0690-5.
18. Song X, Zhang N, Han P, Moon BS, Lai RK, Wang K, et al. Circular RNA profile in gliomas revealed by identification tool UROBORUS. Nucleic Acids Res. 2016;44 9:e87. doi:10.1093/nar/gkw075.
19. Kumar S, Vo AD, Qin F and Li H. Comparative assessment of methods for the fusion transcripts detection from RNA-Seq data. Sci Rep. 2016;6:21597. doi:10.1038/srep21597.

20. Zeng X, Lin W, Guo M and Zou Q. A comprehensive overview and evaluation of circular RNA detection tools. PLOS Computational Biology. 2017;13 6:e1005420. doi:10.1371/journal.pcbi.1005420.

21. Tembe WD, Pond SJ, Legendre C, Chuang HY, Liang WS, Kim NE, et al. Open-access synthetic spike-in mRNA-seq data for cancer gene fusions. BMC Genomics. 2014;15:824. doi:10.1186/1471-2164-15-824.

22. Huang W, Li L, Myers JR and Marth GT. ART: a next-generation sequencing read simulator. Bioinformatics. 2012;28 4:593-4. doi:10.1093/bioinformatics/btr708.

23. Zhang LQ, Cheranova D, Gibson M, Ding S, Heruth DP, Fang D, et al. RNA-seq reveals novel transcriptome of genes and their isoforms in human pulmonary microvascular endothelial cells treated with thrombin. PloS one. 2012;7 2:e31229. doi:10.1371/journal.pone.0031229.

24. Forbes SA, Beare D, Boutselakis H, Bamford S, Bindal N, Tate J, et al. COSMIC: somatic cancer genetics at high-resolution. Nucleic Acids Res. 2017;45 D1:D777-d83. doi:10.1093/nar/gkw1121.

25. Tan S, Gou Q, Pu W, Guo C, Yang Y, Wu K, et al. Circular RNA F-circEA produced from EML4-ALK fusion gene as a novel liquid biopsy biomarker for non-small cell lung cancer. Cell Research. 2018;28 6:693-5. doi:10.1038/s41422-018-0033-7.

26. Rusan M, Li K, Li Y, Christensen CL, Abraham BJ, Kwiatkowski N, et al. Suppression of Adaptive Responses to Targeted Cancer Therapy by Transcriptional Repression. Cancer Discov. 2018;8 1:59-73. doi:10.1158/2159-8290.cd-17-0461.

27. Kim D, Langmead B and Salzberg SL. HISAT: a fast spliced aligner with low memory requirements. Nature Methods. 2015;12:357. doi:10.1038/nmeth.3317.

28. Li H, Handsaker B, Wysoker A, Fennell T, Ruan J, Homer N, et al. The Sequence Alignment/Map format and SAMtools. Bioinformatics. 2009;25 16:2078-9. doi:10.1093/bioinformatics/btp352.

29. Lee M, Lee K, Yu N, Jang I, Choi I, Kim P, et al. ChimerDB 3.0: an enhanced database for fusion genes from cancer transcriptome and literature data mining. Nucleic Acids Research. 2017;45 Database issue:D784-D9.

30. Novo FJ, Mendíbil IOD and Vizmanos JL. TICdb: a collection of gene-mapped translocation breakpoints in cancer. Bmc Genomics. 2007;8 1:33.

31. Korla PK, Cheng J, Huang CH, Tsai JJ, Liu YH, Kurubanjerdjit N, et al. FARE-CAFE: a database of functional and regulatory elements of cancer-associated fusion events. Database (Oxford). 2015;2015 doi:10.1093/database/bav086.

32. Wang Y, Wu N, Liu J, Wu Z and Dong D. FusionCancer: a database of cancer fusion genes derived from RNA-seq data. Diagnostic Pathology. 2015;10 1:131.

33. Zerbino DR, Achuthan P, Akanni W, Amode MR, Barrell D, Bhai J, et al. Ensembl 2018. Nucleic Acids Research. 2018;46 Database issue:D754.

34. Uhrig S, Fröhlich M, Hutter B and Brors B. PO-400 Arriba–fast and accurate gene fusion detection from RNA-seq data. BMJ Publishing Group Limited, 2018.

35. Jasper J, Powers JG and Weigman VJ. STAR-SEQR: Accurate fusion detection and support for fusion neoantigen applications. AACR, 2018.

36. Tan S, Sun D, Pu W, Gou Q, Guo C, Gong Y, et al. Circular RNA F-circEA-2a derived from EML4-ALK fusion gene promotes cell migration and invasion in non-small cell lung cancer. Mol Cancer. 2018;17 1:138. doi:10.1186/s12943-018-0887-9.

37. Cai Z; Xue H; Xu Y; Köhler J; Cheng X; Dai Y; Zheng J; Wang H: Supporting data for "Fcirc: A Comprehensive Pipeline for Exploration of Fusion Linear and Circular RNAs" GigaScience Database. 2020. http://dx.doi.org/10.5524/100734

Figure legends

Figure 1. Fcirc pipeline workflow for exploring linear and circular RNAs of known fusions

Schematic depiction of the five main steps of the Fcirc workflow, which includes the dropping of aligned reads (step 1), the building of a bipartite graph of known fusion gene pairs (step 2), the selection of fusion-related reads (step 3), as well as the reconstruction and verification of fusion genes for linear (step 4) and fusion circular RNAs (step 5 includes transformation of back-spliced reads).

Figure 2. Performance comparison of different gene fusion detection tools in synthetic spike-in actual RNA-Seq data

Comparison of precision (**A**), recall (**B**), F-measure (**C**) and computing time (**D**) across seven fusion detection tools, including Arriba, ChimeraScan, FusionCatcher, JAFFA, STAR-Fusion, STAR-SEQR and Fcirc (red box).

Figure 3. Identification of fusion-supporting reads with different gene fusion detection tools in synthetic spike-in actual RNA-Seq data

The abundance of fusion-supporting reads of nine spiked-in synthetic fusion RNAs was determined by Arriba, ChimeraScan, FusionCatcher, JAFFA, STAR-Fusion, STAR-SEQR and Fcirc in total RNA of the melanoma cell line COLO-829 (n=2 replicates for each fusion gene).

Figure 4. Performance comparison of different gene fusion detection tools in simulated RNA-Seq data

Comparison of precision (**A**), recall (**B**), F-measure (**C**), and computing time (**D**) across seven fusion detection tools, including Arriba, ChimeraScan, FusionCatcher, JAFFA, STAR-Fusion, STAR-SEQR and Fcirc (red box).

Figure 5. Identification of f-circRNAs in paired-end (A) and single-end (B) simulated RNA-Seq data

Fcirc was applied to detect f-circRNAs from four different fusion genes (*EML4-ALK*, *EWSR1-FLI1*, *KMT2A-MLLT3*, and *PML-RARα*) in simulated RNA-Seq datasets. Whereas the control dataset contains only linear fusion transcripts, the investigative dataset included pooled linear/circular fusion transcripts. Different sequencing coverages (20, 50, and 100x) and two read length (50 and 100 bp) were simulated.

Figure 6. Visualization of f-circRNAs

The structure of fusion circular RNAs (n=2 for each fusion gene) which were identified in the paired-end sample analysis with 100x coverage and a read length of 100 bp (Fig. 5A) is visualized for f-circ*EWSR1-FLI1* (**A**), f-circ*EML4-ALK* (**B**), f-circ*PML-RARα* (**C**) and f-circ*KMT2A-MLLT3* (**D**). The distribution of fusion-supporting reads on the fusion region (middle graph) and of f-circRNA-supporting reads on the back-spliced region (lower graph) are depicted.

Additional files

Supplemental_Fig_S1. Eight types of f-circRNAs from four fusion genes (*EML4-ALK*, *EWSR1-FLI1*, *KMT2A-MLLT3*, and *PML-RARα*) designed for simulated RNA-Seq datasets.

Supplemental_Fig_S2. Computing times of Fcirc in simulated single-end RNA-Seq data.

Supplemental_Fig_S3. Performance comparison of different gene fusion detection tools in actual RNA-Seq data (BioProject ID PRJNA350335).

Supplemental_Tab_S1. Artificially designed fusion transcripts in simulated data (genome version: hg38).

Supplemental_Tab_S2. Artificially designed f-circRNAs in simulated data (genome version: hg38).

Supplemental_Tab_S3. Characteristics of fusion transcript detection tools.

Supplemental_Tab_S4. Summary of running parameters of seven fusion detection tools.

Supplemental_Tab_S5. Precision and recall for synthetic RNA-Seq data.

Supplemental_Tab_S6. F-measure for synthetic RNA-Seq data.

Supplemental_Tab_S7. Computing time for synthetic data.

Supplemental_Tab_S8. Precision and recall for simulated RNA-Seq data.

Supplemental_Tab_S9. F-measure for simulated RNA-Seq data.

Supplemental_Tab_S10. Computing time for paired-end simulated RNA-Seq data.

Supplemental_Tab_S11. *EML4-ALK* fusions identified in actual cancer cell-derived RNA-Seq data (BioProject ID PRJNA350335).

Supplemental_Tab_S12. F-circRNAs identified by Fcirc in actual cancer cell-derived RNA-Seq data.

Supplemental_Tab_S13. Precision, recall and F-measure for actual cancer cell-derived RNAseq data (BioProject ID PRJNA350335).

Supplemental_Tab_S14. Fusion genes identified in actual cancer cell-derived RNA-Seq data (BioProject IDs PRJNA350335 and PRJNA315254).

Figure
Click here to access/download;Figure;Figure1.eps

Fig. 1

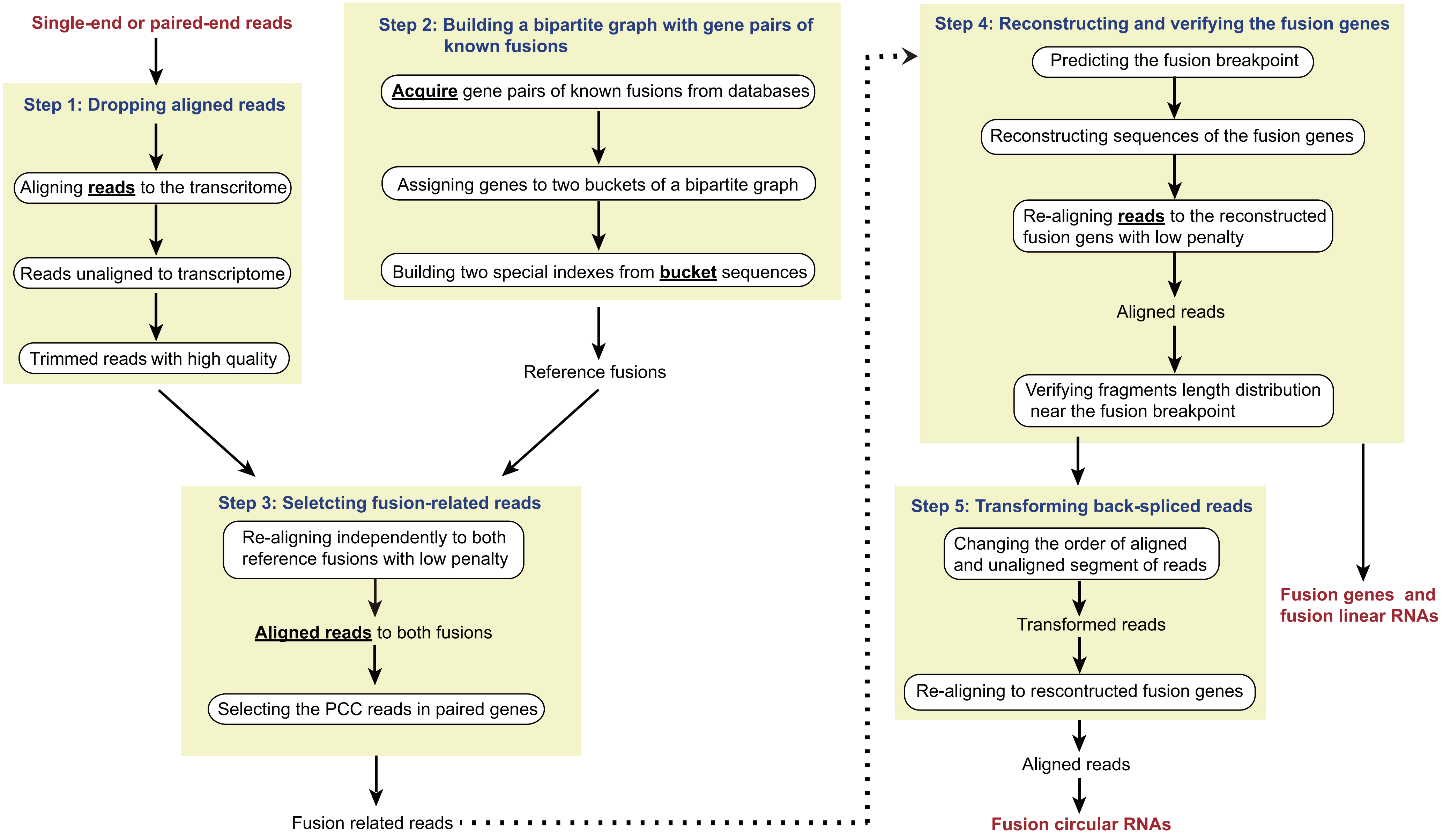

Figure

Click here to access/download;Figure;Figure2.eps

Fig. 2

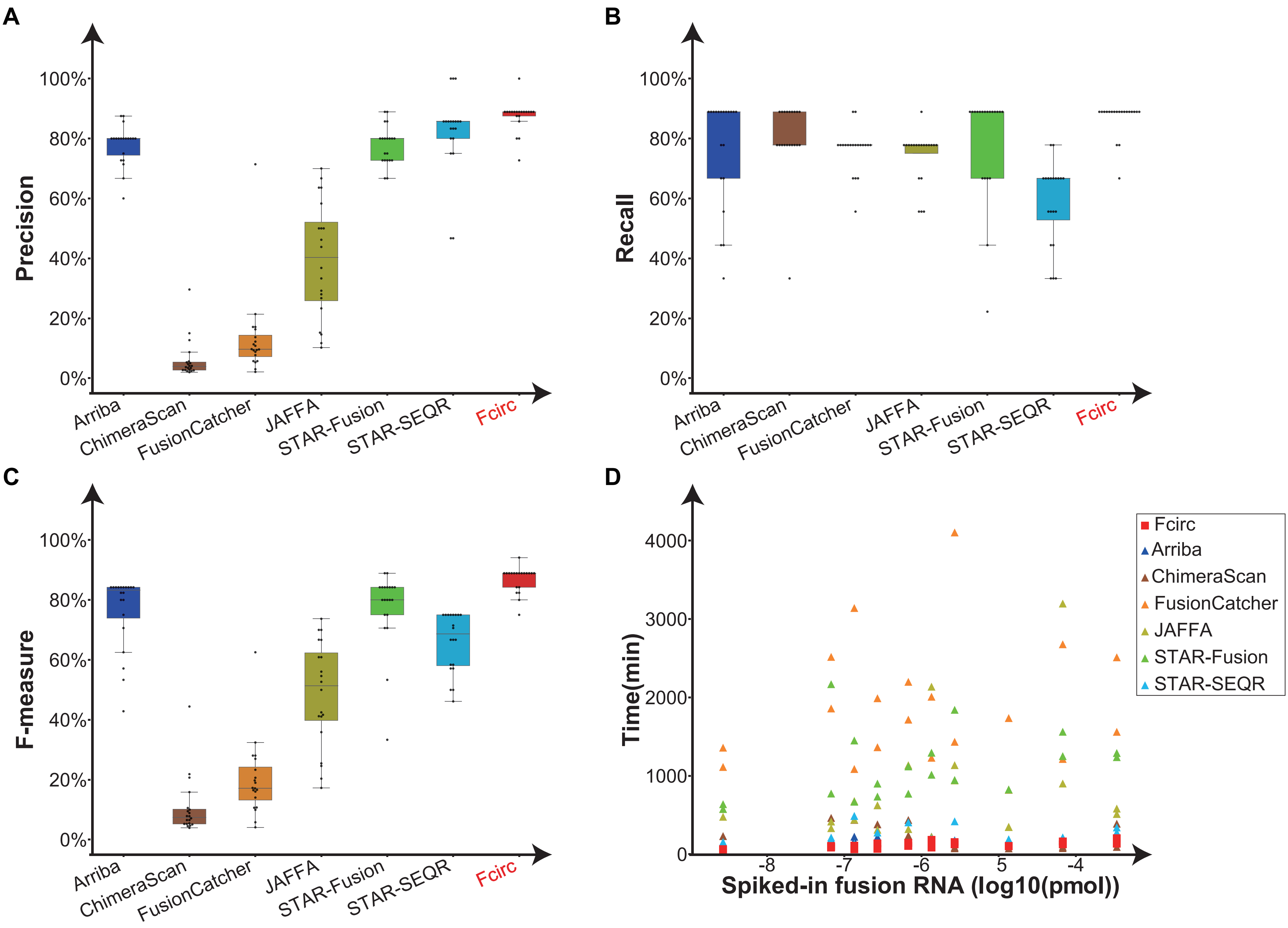

Figure

Click here to access/download;Figure;Figure3.eps

Fig. 3

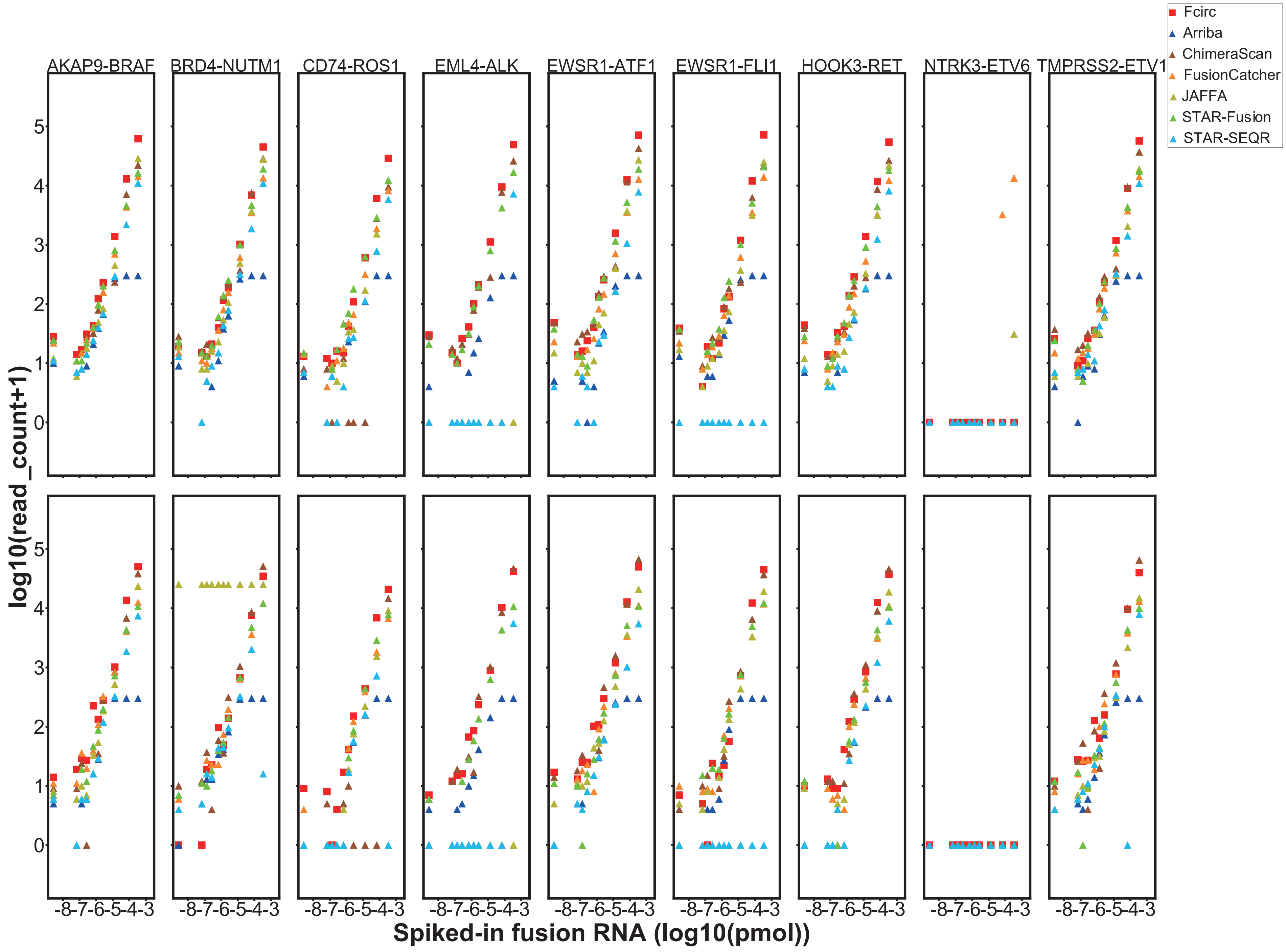

Figure

Click here to access/download;Figure;Figure4.eps

Fig. 4

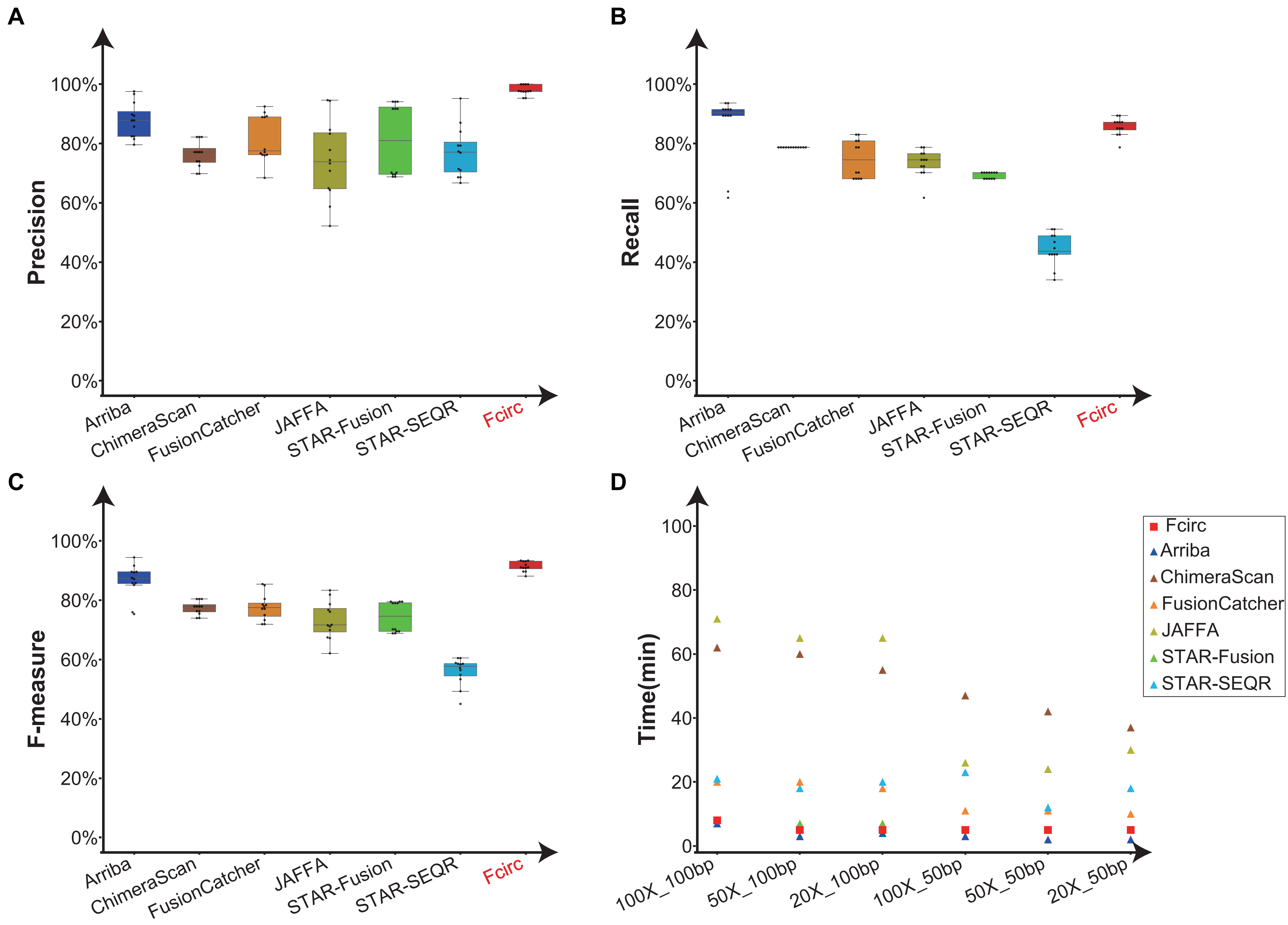

Figure
Click here to access/download;Figure;Figure5.eps

Fig. 5

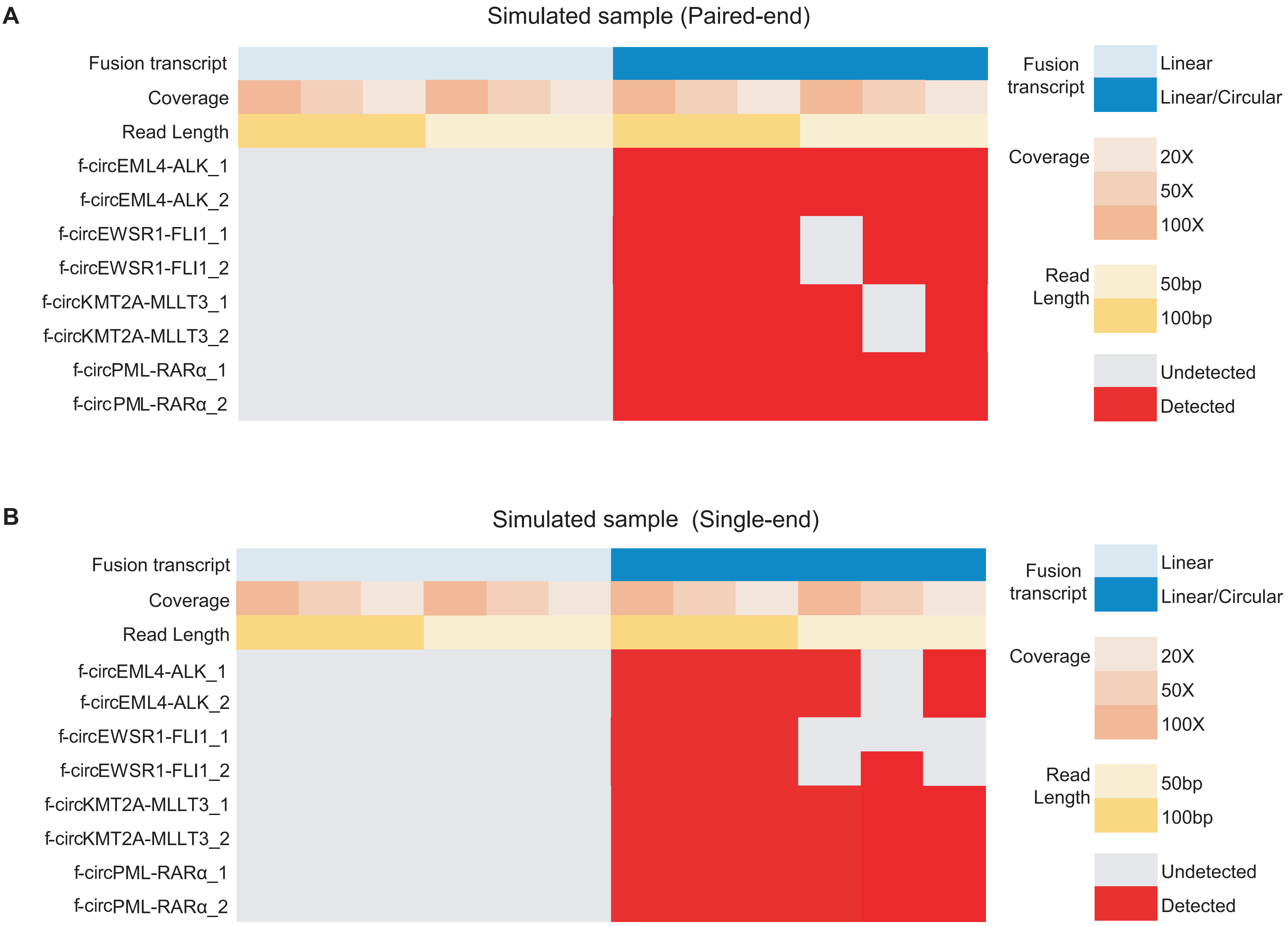

Figure
Click here to access/download;Figure;Figure6.eps

Fig. 6

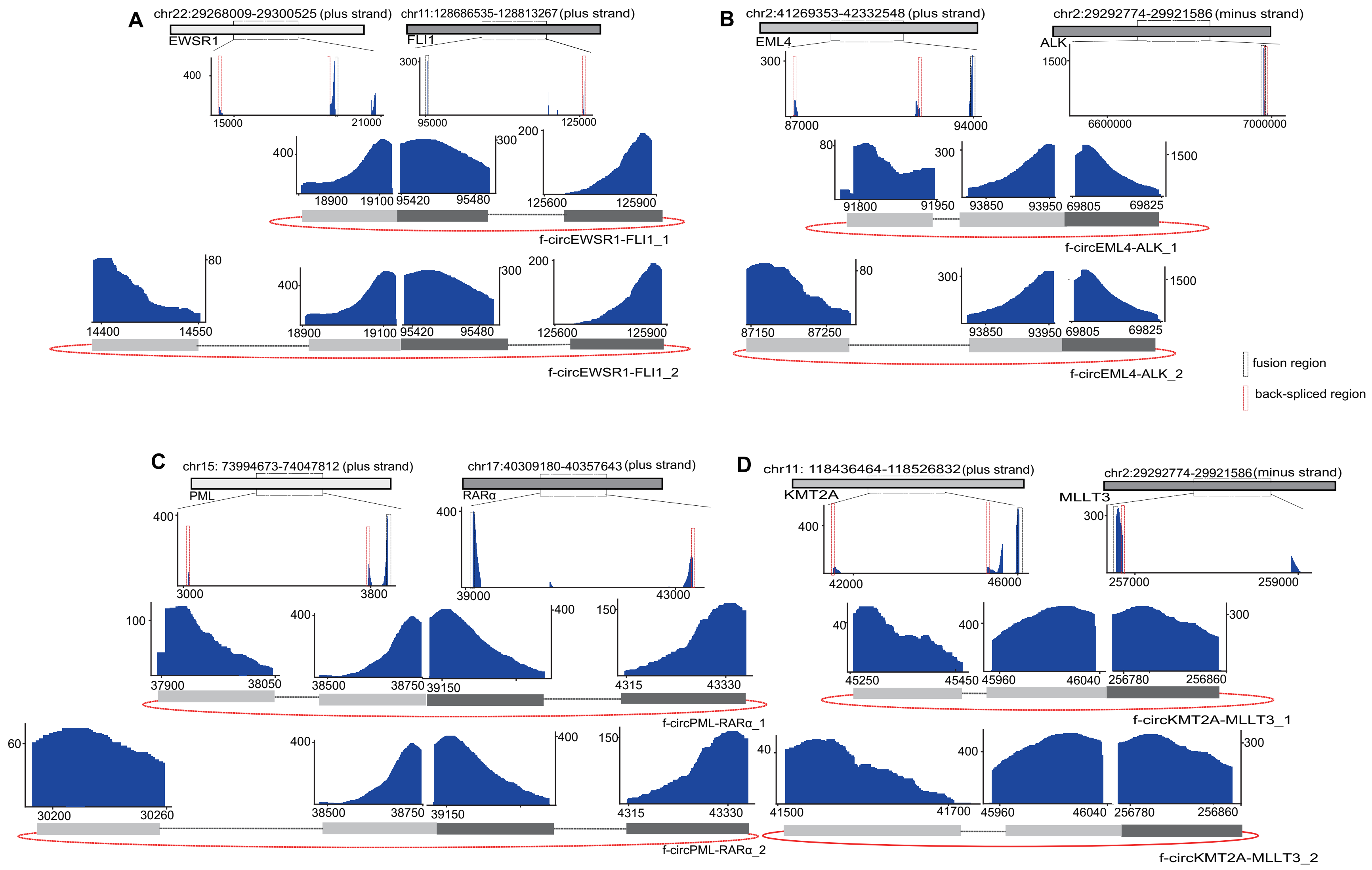

Click here to access/download
Supplementary Material
Supplemental_Figure_S1.tiff

Click here to access/download
Supplementary Material
Supplemental_Figure_S2.tiff

Click here to access/download
Supplementary Material
Supplemental_Figure_S3.tif

Click here to access/download
Supplementary Material
Supplemental_Tab_S1.csv

Click here to access/download
Supplementary Material
Supplemental_Tab_S2.csv

Click here to access/download
Supplementary Material
Supplemental_Tab_S3.csv

Click here to access/download
Supplementary Material
Supplemental_Tab_S4.csv

Click here to access/download
Supplementary Material
Supplemental_Tab_S5.csv

Click here to access/download
Supplementary Material
Supplemental_Tab_S6.csv

Click here to access/download
Supplementary Material
Supplemental_Tab_S7.csv

Click here to access/download
Supplementary Material
Supplemental_Tab_S8.csv

Click here to access/download
Supplementary Material
Supplemental_Tab_S9.csv

Click here to access/download
Supplementary Material
Supplemental_Tab_S10.csv

Click here to access/download
Supplementary Material
Supplemental_Tab_S11.csv

Click here to access/download
Supplementary Material
Supplemental_Tab_S12.csv

Click here to access/download
Supplementary Material
Supplemental_Tab_S13.csv

Click here to access/download
Supplementary Material
Supplemental_Tab_S14.csv
