# Supplementary figures and images for: Fcirc: A comprehensive pipeline for the exploration of fusion linear and circular RNAs
Source: Gigascience. 2020 May 29;9(6):giaa054. doi: 10.1093/gigascience/giaa054 (PMC7259471; doi:10.1093/gigascience/giaa054)

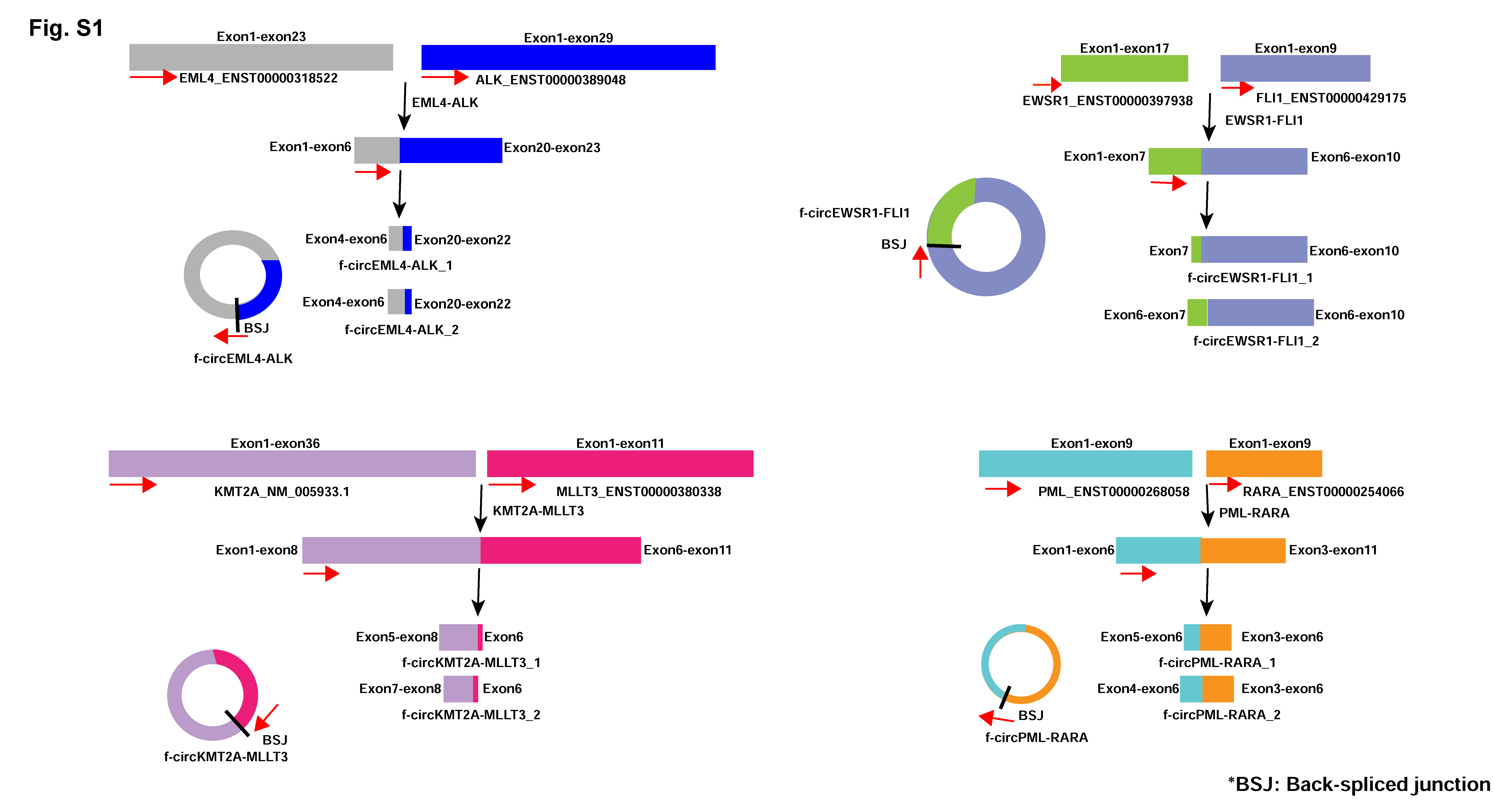

Supplement: giaa054_Supplemental_Files [file giaa054_supplemental_files.zip › Supplemental_Figure_S1.tiff]

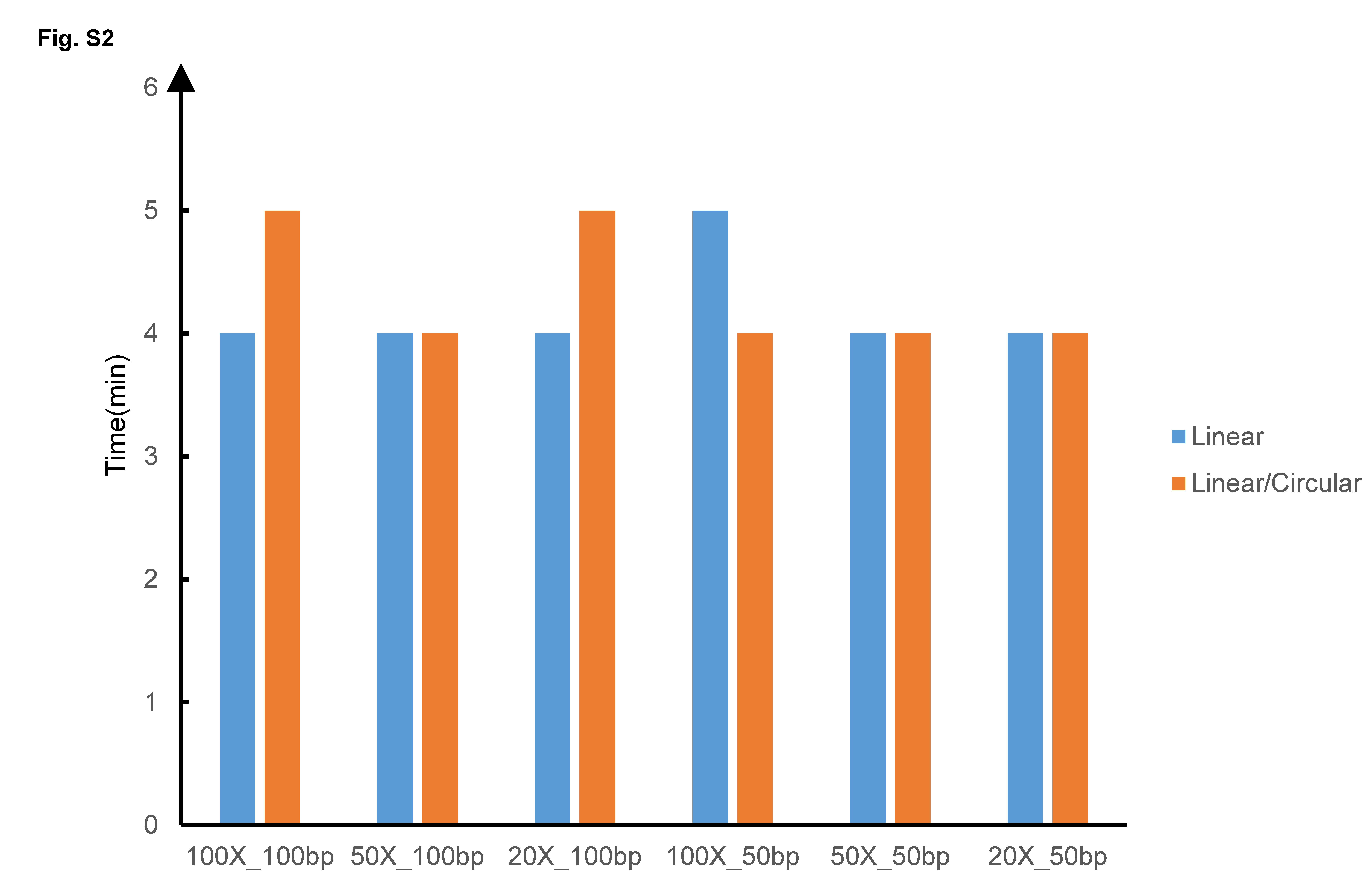

Supplement: giaa054_Supplemental_Files [file giaa054_supplemental_files.zip › Supplemental_Figure_S2.tiff]

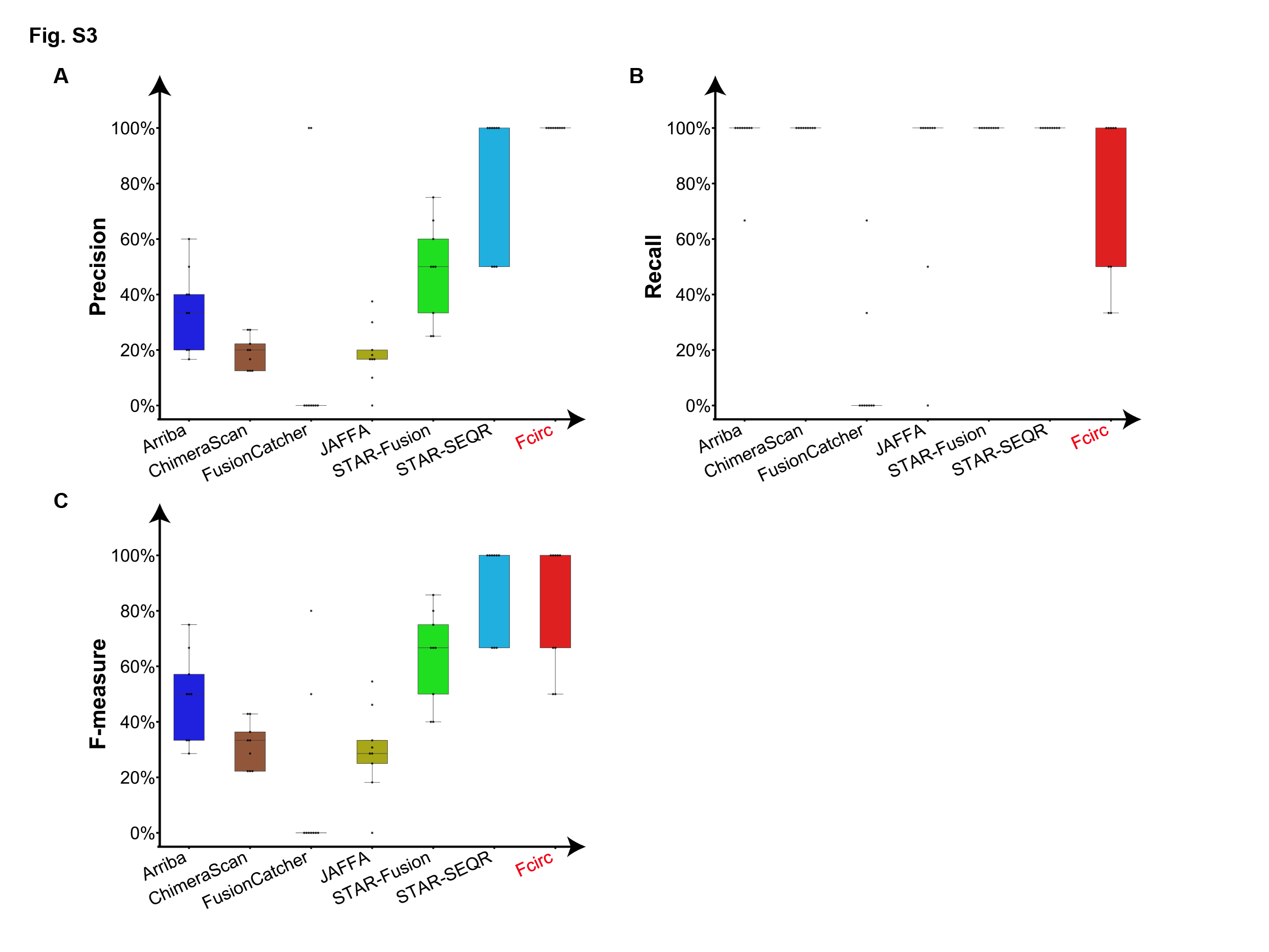

Supplement: giaa054_Supplemental_Files [file giaa054_supplemental_files.zip › Supplemental_Figure_S3.tif]
