# Supplementary material for: Fcirc: A comprehensive pipeline for the exploration of fusion linear and circular RNAs
Source: Gigascience. 2020 May 29;9(6):giaa054. doi: 10.1093/gigascience/giaa054 (PMC7259471; doi:10.1093/gigascience/giaa054)
Supplement: giaa054_Response_to_Reviewer_Comments_Revision_1 [file giaa054_response_to_reviewer_comments_revision_1.pdf]

Author's Response To Reviewer Comments

Close

GIGA-D-19-00383R2
Title: Fcirc: A Comprehensive Pipeline for Exploration of Fusion Linear and Circular RNAs

EDITORS' COMMENTS TO THE AUTHOR
Your manuscript "Fcirc: A Comprehensive Pipeline for Exploration of Fusion Linear and Circular RNAs" (GIGA-D-19-00383R1) has been assessed by our reviewers. Based on these reports, and my own assessment as Editor, I am pleased to inform you that it is potentially acceptable for publication in GigaScience, once you have carried out some essential revisions suggested by our reviewers.
Reply: Thanks so much for your consideration of potential acceptance of our study. We have made the correspond revisions suggested by the reviewers.

Their reports, together with any other comments, are below. Please also take a moment to check our website at https://www.editorialmanager.com/giga/ for any additional comments that were saved as attachments.
In addition, please register any new software application in the bio.tools and SciCrunch.org databases to receive RRID (Research Resource Identification Initiative ID) and biotoolsID identifiers, and include these in your manuscript. This will facilitate tracking, reproducibility and re-use of your tool.
Reply: We have registered the IDs in the bio.tools (id: biotools:Fcirc) and SciCruch.org databases (SCR_018090), which are included in the manuscript.

Once you have made the necessary corrections, please submit a revised manuscript online at:
https://www.editorialmanager.com/giga/
If you have forgotten your username or password please use the "Send Login Details" link to get your login information. For security reasons, your password will be reset.

Please include a point-by-point within the 'Response to Reviewers' box in the submission system. Please ensure you describe additional experiments that were carried out and include a detailed rebuttal of any criticisms or requested revisions that you disagreed with. Please also ensure that your revised manuscript conforms to the journal style, which can be found in the Instructions for Authors on the journal homepage. If the data and code has been modified in the revision process please be sure to update the public versions of this too.
Reply: Regarding the comments of Reviewer 1, we have discussed the fact that our pipeline only detects known fusions with higher specificity and lower false positive (blue contents in the discussion section). In addition, regarding another point that the reviewer is concerned on, Fcirc allows users to add own fusion gene pairs of interest at their convenience. This functionality of Fcirc is described in the discussion section.

In this revised manuscript, we also invited a collaborator of our lab, Jens Köhler, to revise our manuscript. The revisions just work on the writing, making the manuscript more logistic and clear, and not change the results and conclusions. To address his contribution on this study, Jens Köhler was added to the co-authors in the revised manuscript. We've highlighted this change in the authors list. In addition, we have also improved the quality of the figures.

REVIEWERS' COMMENTS TO THE AUTHOR
Reviewer #1: The authors partially answered my comments. I believe it is important that the authors highlight in the discussion the fact that their pipeline will only detect known fusions, which gives it increased specificity.
Reply: We appreciate the concerns of Reviewers 1 and have the corresponding discussion regarding this point (blue contents in the discussion section). In our revised manuscript, we've discussed that our method differs from other published fusion detection tools by the fact that it requires information on already known gene fusions as reference to build the bipartite graph of gene pairs. Hence, the Fcirc algorithm detects RNAs from known fusion events with increased specifity and lower false positive rate.

Accounting for the limitation of depending on known fusions with the cost of losing the ability to identify new fusion genes, Fcirc regularly updates information on newly emerging fusion genes from multiple databases (COSMIC, ChimerDB, TicDB, FARE-CAFÉ, FusionCancer). Moreover, users have the option to add own fusion gene pairs of interest at their convenience.

Of note some of the software used can also take list of genes involved in fusion as input (Arriba for instance). Have the authors provided the software with the list of the expected genes in the synthetic RNA-Seq experiment, simulated set, and real RNA-Seq? If not this must be performed as this functionality of the other software was omitted.
Reply: Thanks so much for this nice comment! Fcirc allows users to add the expected fusion genes as an input. We make this option accounting for the limitation of Fcirc, which depends on known fusions, and also considering a scenario where some researchers may have some specific fusions they are interested in. So users have the option to add own fusion gene pairs of interest at their convenience. But in the performance evaluation using the synthetic RNA-Seq, simulated set, and actual RNA-Seq data, we do not provide Fcirc with the list of the expected genes to avoid the bias of the evaluation.

Close
