# Supplementary material for: Fcirc: A comprehensive pipeline for the exploration of fusion linear and circular RNAs
Source: Gigascience. 2020 May 29;9(6):giaa054. doi: 10.1093/gigascience/giaa054 (PMC7259471; doi:10.1093/gigascience/giaa054)
Supplement: giaa054_Response_to_Reviewer_Comments_Original_Submission [file giaa054_response_to_reviewer_comments_original_submission.pdf]

Author's Response To Reviewer Comments

Close

GIGA-D-19-00383
Title: Fcirc: A Comprehensive Pipeline for Exploration of Fusion Linear and Circular RNAs

EDITORS' COMMENTS TO THE AUTHOR
Your manuscript "Fcirc: A Comprehensive Pipeline for Exploration of Fusion Linear and Circular RNAs" (GIGA-D-19-00383) has been assessed by our reviewers. Although it is of interest, we are unable to consider it for publication in its current form. The reviewers have raised a number of points which we believe would improve the manuscript and may allow a revised version to be published in GigaScience.

GigaScience has a strong focus on reproducibility and reuse, and a reviewer has noticed that some of the software used in the comparison are no longer supported - please acknowledge e some of the limitations (no identification of novel events), use of no longer supported software as well as older versions which might affect the outcomes of some of the comparisons. You must also provide better details, such as parameters to run the different pipelines. Furthermore, please also expand the bench marking to include other tools - to clearly demonstrate the advantage of using Fcirc. More clarification on scoring parameters and how the "synthetic spike-in real RNA-Seq data" was generated, must be provided.

Their reports, together with any other comments, are below. Please also take a moment to check our website at https://www.editorialmanager.com/giga/ for any additional comments that were saved as attachments.

In addition, please register any new software application in the bio.tools and SciCrunch.org databases to receive RRID (Research Resource Identification Initiative ID) and biotoolsID identifiers, and include these in your manuscript. This will facilitate tracking, reproducibility and re-use of your tool.

If you are able to fully address these points, we would encourage you to submit a revised manuscript to GigaScience. Once you have made the necessary corrections, please submit online at: https://www.editorialmanager.com/giga/

If you have forgotten your username or password please use the "Send Login Details" link to get your login information. For security reasons, your password will be reset.

Please include a point-by-point within the 'Response to Reviewers' box in the submission system. Please ensure you describe additional experiments that were carried out and include a detailed rebuttal of any criticisms or requested revisions that you disagreed with. Please also ensure that your revised manuscript conforms to the journal style, which can be found in the Instructions for Authors on the journal homepage. If the data and code has been modified in the revision process please be sure to update the public versions of this too.

The due date for submitting the revised version of your article is 02 Mar 2020.

I look forward to receiving your revised manuscript soon.

Best wishes,
Nicole Nogoy, Ph.D GigaScience www.gigasciencejournal.com

Reply: We sincerely appreciate the editor's comments. In our revised manuscript, we have used the latest version of STAR-Fusion, JAFFA, FusionCatcher to re-evaluate the performance of these tools. And two new tools, Arriba and STAR-SEQR, which were reported to have better performance compared with other 20 tools according to the study of Hass et al [1], were also applied for comparison. The detailed parameters used to run these tools are shown in Supple.Tab_S1, S7. In addition, regarding the impacts of scoring parameters on false positives as well as the detailed information of synthetic spike-in real

RNA-Seq data, we have made point-to-point reply to reviewers and made the corresponding revisions in the revised manuscript. In addition, we've registered our software in the bio.tools (biotools:Fcirc) and SciCrunch.org databases (SCR_018089) and included the information in our revised manuscript. Furthermore, we've expanded the benchmarking to real data and clearly demonstrated the advantage of using Fcirc. All of the change in our revised manuscript has been marked in red. We hope you will judge our paper of sufficient interest to merit consideration in GigaScience.

REVIEWERS' COMMENTS TO THE AUTHOR
Reviewer #1: The manuscript by Cai et al describes a new pipeline to annotate fusion transcripts and fusion circular RNAs. The manuscript is overall well written and the tool described of interest especially as several of the tools for gene fusion discovery currently available have relative high false positive rate.

One of the limitation of the application is that it relies on providing known fusions for the detection and does not offer the possibility to explore potential novel events, in doing so the authors limit the use of their pipeline.

Reply: We appreciate the comments and concerns of Reviewer 1. Regarding the limitation of our method, in our revised manuscript we've included two newly developed fusion detection tools, Arriba (v1.1.0) and STAR-SEQR (v0.6.7), which were reported to have better performance compared with other 20 tools according to the study of Hass et al [1], to evaluate the performance of Fcirc (Fig.2, Fig4, Supplemental_Fig_S3).

Some of the software used in the comparison are not maintained (Chimera-Scan - last commit in 2012). Additionally other software (STAR-Fusion, JAFFA) have more recent versions including potential performance improvements (STAR-FUSION), the authors should be careful in drawing conclusions in the comparisons, and acknowledge those potential issues. Additionally the authors should provide the parameters used to run the different pipelines.

Reply: In our revised manuscript, we used the latest version of STAR-Fusion (v1.8.1), JAFFA (v1.09), FusionCatcher (v1.00) to re-evaluate the performance of these tools. In addition, we added two newly developed fusion detection tools, Arriba (v1.1.0) and STAR-SEQR (v0.6.7). According to the study of Haas et al., by comparing 20 fusion detection tools they found that Arriba (v1.1.0) and STAR-SEQR (v0.6.7) gained better performance [1]. So we included these two tools in our revised manuscript. Overall, Fcirc exhibited an advantage compared to these 6 methods in prediction performance and computing time both for synthetic spike-in real RNA-Seq data (Fig.2) and simulated RNA-Seq data (Fig.4).

To make it clear for the readers to understand the parameters of the different fusion detection tool, we made a table to provide the detailed information (Supplemental_Tab_S1) of tools and parameters that were used to run these tools (Supplemental_Tab_S7).

The authors selected 47 fusions from COSMIC, the criteria for selection need to be provided.

Reply:We randomly selected 47 fusions, with high, median and low occurrence frequency in cancers, from the Catalogue Of Somatic Mutations In Cancer (COSMIC) database. We've added the selection criteria in Materials and Methods section of our revised manuscript.

Reviewer #2: In the present manuscript, Zhaoqing Cai and colleagues describe the development of Fcirc, a computational pipeline to detect fusion linear and circular RNAs. The concept of detection fusion transcripts in RNA sequencing data is not new, yet the approach the authors have taken by only looking at known fusion genes drastically increases the performance and decreases the number of false positives. In addition, the tool is also capable of finding fusion circular RNAs. As the authors have indicated, the approach does effectively limit the capability of the tool to find novel fusion events outside of annotated fusion genes.

Major comments
It is unclear how useful this tool will be given that it can only detect fusion events in known fusion genes and the limited benchmarking on real data is not really convincing. I would suggest expanding this part of the benchmarking and also include the other tools here to clearly show the advantage of Fcirc.

Reply: We understand the concern of Reviewer 2. In our revised manuscript, we've added two newly developed fusion detection tools, Arriba (v1.1.0) and STAR-SEQR (v0.6.7), that were reported to have

better performance compared with other 20 tools according to the study of Hass et al.[1], on real data and simulated data evaluation.

Furthermore, we expanded the benchmarking on real data. Differing from synthetic spike-in real data, which was generated for comparative assessment and collaborative development of novel gene fusions detection algorithms, this real data was mainly used to detect f-circRNAs and didn't provide which fusions were truly present in cell lines. To expand the benchmarking on real data, in an alternative way we defined fusions that were detected by at least 4 tools with supporting reads more than 10 as true positives, and then compared the performance of Fcirc with other 6 tools.

Regarding synthetic spike-in real data, we've made the reply in the section below and added the detailed description of this data to Materials and Methods section.

Overall, Fcirc exhibited an advantage compared to other 6 tools in prediction performance both for synthetic spike-in real RNA-Seq data (Fig.2) and simulated RNA-Seq data (Fig.4). As for real data, Fcirc had greater F-measure values, with an excellent precision and a bit decreased recall (Supplemental_Fig_S3).

Page 4, line 76 The authors claim that the "scoring parameters need to be strict to make reads with perfect matches and align few mismatches". But since the unmapped reads are used to find fusion genes, wouldn't making the parameters stricter results in more unmapped reads and thus higher change of false positives?

Reply: We thank the reviewer for this nice suggestion. To make sure how the parameters affect the results, we applied two scoring parameters: our previous stricter one and looser one (by default). When the stricter one was applied, indeed, more unmapped reads and fusion genes were called as shown in the table. That results in the decreased precision and F-measure values as what the reviewer concerned on(Supplemental_Tab _S15). So in our revised manuscript, we applied the default looser parameters to detect fusions.

Page 8 It is unclear how the "synthetic spike-in real RNA-Seq data" was generated. How are the synthetic RNA transcripts created (technology/company)? What RNA sample was used as a basis and what are the concentrations of the sample and the spike-in? What library prep. and sequencer were used? Ideally this data should be deposited in a public resource.

Reply: We sincerely appreciate the comments of Reviewer 2 in this point. In our revised manuscript, we've added the detailed description of this data to Materials and Methods section.

Synthetic spike-in real RNA-Seq data [2] are from a study by Tembe W et al. They generated this publicly available gene-fusion RNA-seq data, with an aim to comparative assessment and collaborative development of novel gene fusions detection algorithms. In this study, they have generated nine synthetic poly-adenylated RNA transcripts that correspond to previously reported oncogenic gene fusions, including EWSR1-ATF1, TMPRSS2-ETV1, EWSR1-FLI1, NTRK3-ETV6, CD74-ROS1, HOOK3-RET, EML4-ALK, AKAP9-BRAF, and BRD4-NUTM1. Equimolar amounts of all nine synthetic gene fusion RNAs were pooled together and this pool was titrated into total RNA from the melanoma cell line COLO-829 at ten different abundances. Libraries were prepared for sequencing using the Illumina TruSeq Stranded mRNA LT Sample Preparation Kit and sequenced on an Illumina HiSeq 2500 (2 × 101 cycles). The data is available in FASTQ format from the Short Read Archive under accession number SRP043081.

Minor comments and typos

Page 1, line 33 The computational methods … -> Computational methods
Reply: We've made the correction.

Page 4, line 81 change -> changed
Reply: We've made the correction.

Page 10, Figure 2D Why do the authors assume that the runtime would increase with the amount (pmol)

of spike-in? Would it not be a function of the number of reads generated in the sequencing? It would actually make more sense to see an effect of the spike-in amount on the precision and recall (figure 2A-C).
Reply: Here we don't assume that the runtime increases with the amount of spike in. Actually there is no obvious correlation between the runtime and the amount of spike-in (Fig.2D). I agree with the reviewer that it would make sense to see that prediction performance increases with the amount of spike-in. Because we use fusion-supporting reads to detect fusion genes, and the number of fusion-supporting reads apparently increases with the amount of spike-in (Fig.3).

Page 14, line 287 the fusion transcript -> fusion transcripts
Reply: We've made the correction.

Figure 2,3, elsewhere The correct abbreviation for picomoles is pmol.
Reply: We've made the correction.

Reference
1. Haas BJ, Dobin A, Li B, Stransky N, Pochet N and Regev A. Accuracy assessment of fusion transcript detection via read-mapping and de novo fusion transcript assembly-based methods. Genome Biology. 2019;20 1:213.
2. Tembe W, Pond S, Legendre C, Chuang H, Liang WS, Kim N, et al. Open-access synthetic spike-in mRNA-seq data for cancer gene fusions. BMC Genomics. 2014;15 1:824-.

Close
