# Supplementary material for: Fcirc: A comprehensive pipeline for the exploration of fusion linear and circular RNAs
Source: Gigascience. 2020 May 29;9(6):giaa054. doi: 10.1093/gigascience/giaa054 (PMC7259471; doi:10.1093/gigascience/giaa054)
Supplement: giaa054_Reviewer_2_Report_Original_Submission — Pieter-Jan Volders -- 12/3/2019 Reviewed [file giaa054_reviewer_2_report_original_submission.pdf]

Reviewer Report

Title: Fcirc: A Comprehensive Pipeline for Exploration of Fusion Linear and Circular RNAs

Version: Original Submission **Date:** 12/3/2019

Reviewer name: Pieter-Jan Volders

Reviewer Comments to Author:

In the present manuscript, Zhaoqing Cai and colleagues describe the development of Fcirc, a computational pipeline to detect fusion linear and circular RNAs.
The concept of detection fusion transcripts in RNA sequencing data is not new, yet the approach the authors have taken by only looking at known fusion genes drastically increases the performance and decreases the number of false positives. In addition, the tool is also capable of finding fusion circular RNAs. As the authors have indicated, the approach does effectively limit the capability of the tool to find novel fusion events outside of annotated fusion genes.
Major comments
It is unclear how useful this tool will be given that it can only detect fusion events in known fusion genes and the limited benchmarking on real data is not really convincing. I would suggest expanding this part of the benchmarking and also include the other tools here to clearly show the advantage of Fcirc.
Page 4, line 76
The authors claim that the "scoring parameters need to be strict to make reads with perfect matches and align few mismatches". But since the unmapped reads are used to find fusion genes, wouldn't making the parameters stricter results in more unmapped reads and thus higher change of false positives?
Page 8
It is unclear how the "synthetic spike-in real RNA-Seq data" was generated. How are the synthetic RNA transcripts created (technology/company)? What RNA sample was used as a basis and what are the concentrations of the sample and the spike-in? What library prep. and sequencer were used? Ideally this data should be deposited in a public resource.
Minor comments and typos
Page 1, line 33
The computational methods … -> Computational methods
Page 4, line 81
change -> changed
Page 10, Figure 2D
Why do the authors assume that the runtime would increase with the amount (pmol) of spike-in? Would it not be a function of the number of reads generated in the sequencing? It would actually make more sense to see an effect of the spike-in amount on the precision and recall (figure 2A-C).
Page 14, line 287
the fusion transcript -> fusion transcripts
Figure 2,3, elsewhere

The correct abbreviation for picomoles is pmol.

Methods

Are the methods appropriate to the aims of the study, are they well described, and are necessary controls included? Choose an item.

Conclusions

Are the conclusions adequately supported by the data shown? Choose an item.

Reporting Standards

Does the manuscript adhere to the journal's guidelines on minimum standards of reporting? Choose an item.

Choose an item.

Statistics

Are you able to assess all statistics in the manuscript, including the appropriateness of statistical tests used? Choose an item.

Quality of Written English

Please indicate the quality of language in the manuscript: Choose an item.

Declaration of Competing Interests

Please complete a declaration of competing interests, considering the following questions:

- Have you in the past five years received reimbursements, fees, funding, or salary from an organisation that may in any way gain or lose financially from the publication of this manuscript, either now or in the future?
- Do you hold any stocks or shares in an organisation that may in any way gain or lose financially from the publication of this manuscript, either now or in the future?
- Do you hold or are you currently applying for any patents relating to the content of the manuscript?
- Have you received reimbursements, fees, funding, or salary from an organization that holds or has applied for patents relating to the content of the manuscript?
- Do you have any other financial competing interests?
- Do you have any non-financial competing interests in relation to this paper?

If you can answer no to all of the above, write 'I declare that I have no competing interests' below. If your reply is yes to any, please give details below.

I declare that I have no competing interests

I agree to the open peer review policy of the journal. I understand that my name will be included on my report to the authors and, if the manuscript is accepted for publication, my named report including any attachments I upload will be posted on the website along with the authors' responses. I agree for my report to be made available under an Open Access Creative Commons CC-BY license (http://creativecommons.org/licenses/by/4.0/). I understand that any comments which I do not wish to be included in my named report can be included as confidential comments to the editors, which will not be published.

Choose an item.

To further support our reviewers, we have joined with Publons, where you can gain additional credit to further highlight your hard work (see: https://publons.com/journal/530/gigascience). On publication of this paper, your review will be automatically added to Publons, you can then choose whether or not to claim your Publons credit. I understand this statement.

Yes Choose an item.
